# The Impact of Ambient Ozone Exposure on Respiratory Health in the U.S. Populations: A Systematic Review

**DOI:** 10.1007/s40572-026-00568-0

**Published:** 2026-10-17

**Authors:** Yoojin Cho, Yu Yu, Xinyu Zhang, Michael Kleeman, Michael Jerrett, Ninez A. Ponce

**Affiliations:** 1 Department of Epidemiology, Fielding School of Public Health, University of California Los Angeles, Los Angeles, CA USA; 2 Center for Health Policy Research, University of California Los Angeles, Los Angeles, CA USA; 3 Department of Civil and Environmental Engineering, University of California Davis, Davis, CA USA; 4 Department of Environmental Health Sciences, Fielding School of Public Health, University of California Los Angeles, Los Angeles, CA USA; 5 Department of Health Policy and Management, Fielding School of Public Health, University of California Los Angeles, Los Angeles, CA USA

**Keywords:** Ozone, Pulmonary functions, Asthma-related symptoms/ED visits/hospitalizations, Respiratory-related mortality and morbidity

## Abstract

Ozone, a photochemically generated air pollutant, continues to pose a major concern for public health in the United States, with some 125 million people living in areas that exceed national ambient air quality standards set by the United States Environmental Protection Agency. To fully understand the potential impacts of ambient ozone exposure on respiratory health, we conducted a systematic review of United States epidemiologic and experimental studies published from 1980 to 2026, encompassing both acute and chronic exposure windows. This review focused on three key domains: (1) pulmonary function, (2) asthma-related symptoms and emergency department visits/hospitalizations, and (3) other respiratory-related morbidity and mortality. Findings across diverse study designs demonstrated adverse respiratory effects, with children, older adults, and individuals with pre-existing respiratory conditions being disproportionately affected. This review is among the first to comprehensively synthesize ozone-related respiratory health outcomes including pulmonary functions outcomes across both experimental and population-based observational studies—highlighting consistent decrements in lung function, even at concentrations below current regulatory standards. Additionally, this work incorporated ozone-associated school absenteeism, a sensitive yet underexamined indicator for pediatric respiratory health outcome. The observed associations across multiple outcomes and exposure durations underscored the importance of strengthening ambient air quality standards and prioritizing interventions toward high-risk populations. Continued increases in ambient ozone exposure even in the presence of regulatory action and increasing contributions from sources related to climate change such as wildfires emphasize the importance of sustained research and public health planning to safeguard respiratory health in vulnerable communities.

**Graphical Abstract:**

**Figure:**
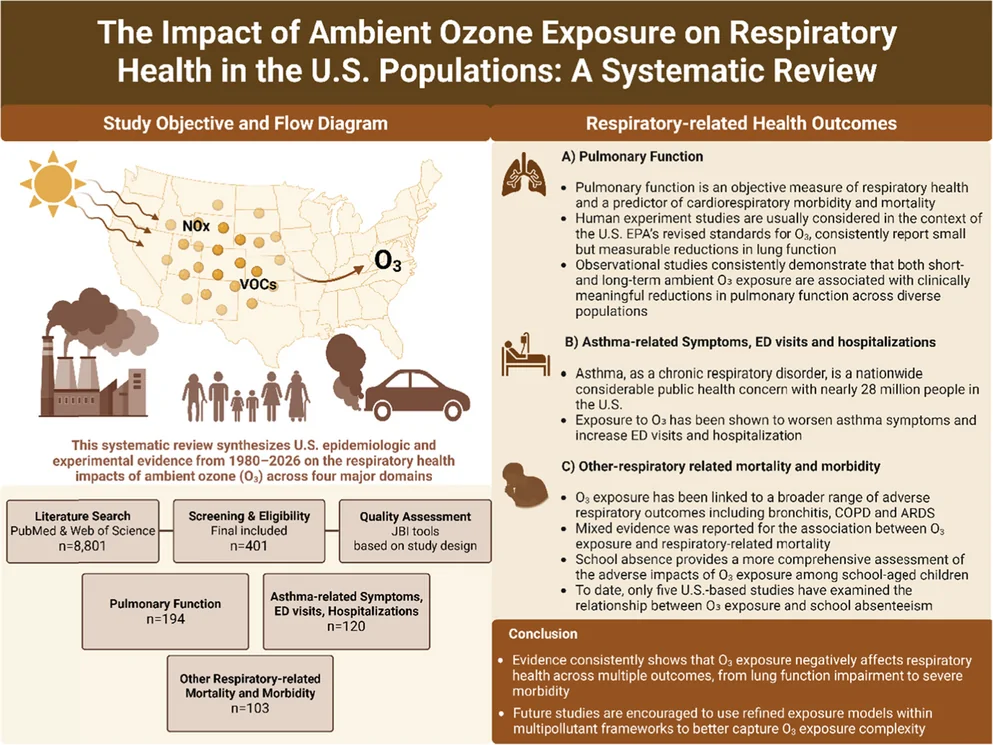

**Supplementary Information:**

The online version contains supplementary material available at https://doi.org/10.1007/s40572-026-00568-0.

## Introduction

Ground-level ozone (O_3_) is a secondary air pollutant produced predominantly by photochemical reactions involving nitrogen oxides and volatile organic compounds in the presence of sunlight [1]. Anthropogenic sources, including traffic and industrial emissions, contribute substantially to these precursors. Additionally, wildfires—which are increasingly fueled by human-related climate change—also generate large quantities of precursor pollutants [2, 3]. In the United States (U.S.), O_3_ concentrations frequently exceed health-protective standards, especially during the warm season. Numerous studies have reported associations between O_3_ exposure and respiratory health outcomes, including asthma exacerbation and increased emergency department (ED) visits/hospitalizations, and respiratory-related mortality [4–6]. Notably, these associations have been observed even at O_3_ levels below the current 8-hour National Ambient Air Quality Standard (NAAQS) of 70 parts per billion (ppb) [7–10]. Despite the expanding body of literature regarding the respiratory impacts of O_3_ exposure, no prior study has comprehensively synthesized findings across a broader spectrum including pulmonary function indicators such as forced expiratory volume in one second (FEV_1_), forced vital capacity (FVC), and peak expiratory flow rate (PEFR), asthma-related health events and extending to broader respiratory-related morbidity and mortality. Additionally, although school absenteeism is a valuable indicator of children’s overall health and can help assess the broader impacts of O_3_ exposure among school-aged children, it has been largely overlooked in previous reviews.

This systematic review examined existing studies on the respiratory-related health impacts of O_3_exposure in the U.S. population. We focused on three major outcome domains: (1) pulmonary function, (2) asthma-related symptoms, ED visits/hospitalizations, and (3) other respiratory-related morbidity and mortality.

## Study Method

**Fig. 1:**
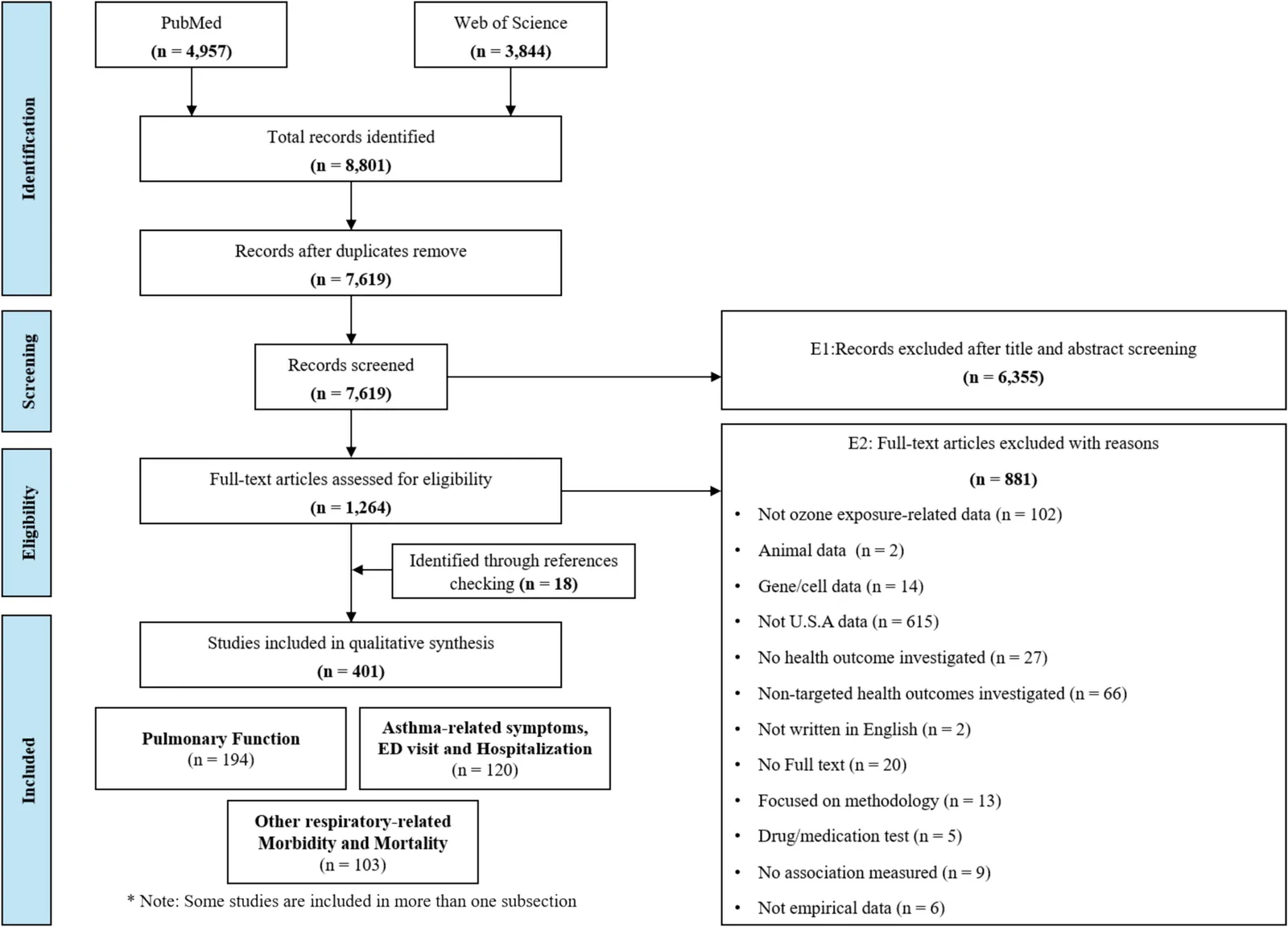
PRISMA flow diagram of the search, screening, and selection process of studies on ozone exposure and respiratory health published between 1980 and 2026

This systematic review was conducted in accordance with the Preferred Reporting Items for Systematic Reviews and Meta-Analyses (PRISMA 2020) guidelines to ensure transparency and methodological rigor throughout the review process. We started a literature search using PubMed (https://pubmed.ncbi.nlm.nih.gov/) and Web of Science (https://www.webofscience.com/) to identify studies examining the health impacts of O_3_ exposure, specifically in relation to respiratory outcomes including pulmonary functions, asthma-related symptoms, ED visits/hospitalizations, within the context of the U.S. Search terms combined multiple ozone-related keywords (e.g., “O_3_,” “ozone,” “trioxygen”) with a broad range of respiratory and clinical outcome terms (e.g., pulmonary function, asthma, respiratory symptoms, emergency department visits, and hospital admissions), and searches were restricted to U.S.-based epidemiological and experimental studies. (More details are provided in the Supplementary Materials). This review encompassed full-text articles published from January 1980 through August 2026. A total of 8,801 records were identified, including 4,957 from PubMed and 3,844 from Web of Science. After removing duplicates, we conducted a first-round screening based on titles and abstracts to exclude those irrelevant to the research question. Full texts of the remaining articles were assessed for eligibility based on the predefined inclusion criteria. Among several exclusion criteria, studies conducted outside the U.S. were excluded to ensure consistency with U.S.-specific O_3_ regulatory frameworks, exposure metrics, population characteristics, and healthcare contexts, thereby facilitating the synthesis of policy-relevant evidence on O_3_-related respiratory health impacts in the U.S. Additionally, 18 relevant articles were identified through reference checking of included studies that were not captured by the original search strategies. Following this process, a total of 401 articles were included in the final review (Fig. 1). Three authors conducted screening and eligibility assessments, independently classifying studies for inclusion or exclusion, and any discrepancies were resolved through discussion until consensus was reached to ensure methodological rigor and reproducibility.

To evaluate the methodological rigor of the studies included in this review, we performed a structured quality assessment using Joanna Briggs Institute (JBI) Critical Appraisal Tools―a validated tool tailored to each study design by three authors independently. Any differences in appraisal were resolved through collective discussion, ensuring consistency and methodological rigor in the assessment. Final scores were determined only after agreement was achieved between authors. According to JBI critical appraisal tools, overall study quality was generally rated as low to moderate risk of bias across most outcome domains, indicating that the majority of included studies were methodologically acceptable. Relatively higher risk-of bias quality ratings were more frequently observed among human experimental studies assessing pulmonary function. Because no JBI tool is specifically tailored to controlled experimental designs, these studies were appraised using a modified version of the JBI randomized controlled trial checklist, which may have contributed to this higher risk of bias rating in this domain. Further details can be found in Supplementary materials and figures (Figure S1–S3).

## Pulmonary Function

O_3_ exposure impacts on lung function have been well documented in human experiments and observational studies during the past several decades [11–20]. Pulmonary function indices, such as FEV₁, FVC and indicators of small-airway function which is particularly sensitive to O_3_ exposure―peak expiratory flow (PEF) and forced expiratory flow between the 25th and 75th percentile of FVC (FEF_25–75_), which is also known as maximum mid-expiratory flow (MMEF), usually offer sensitive and quantitative markers that reflect both early and clinically significant respiratory changes in response to O_3_ exposure. In addition to spirometric decrement, other effects such as increased airway reactivity and inflammation or injury including cell counts and cytokine levels such as tumor necrosis factor (TNF), interleukin (IL)-6 and neutrophil recruitment in bronchoalveolar lavage fluid (BALF) were also reported. These inflammatory markers serve as biologically plausible intermediates linking O_3_ exposure to clinical respiratory outcomes.

### Human Experimental Studies

The most commonly used format before 2000 to evaluate the short-term or acute O_3_ exposure on lung function was human experimental study (Table 1 and Table S1). Human tests were usually conducted in an exposure chamber (also called chamber study) and involved healthy adult volunteers who were exposed to controlled O_3_ concentrations ranging from environmentally relevant levels to those exceeding current ambient standards, sometimes in combination with exercise protocols to simulate real-world ventilation rates. Lung function indices such as FEV₁ and FVC are commonly assessed pre- and post-exposure, alongside inflammation markers and symptoms. Some studies involved square wave exposures where O_3_ concentration was maintained at a constant value throughout the exposure period [21]. Others conducted triangular wave exposures, where O_3_ concentration gradually increased to a peak level and then reduced similarly in a stepwise fashion. Other measurements besides the O_3_ concentration protocol that have been manipulated in chamber studies include exercise (with/without), exposure duration, and exposure method (facemask vs. whole-body exposure in a chamber) [22]. Human experimental studies usually investigated different types of exposure regimens and included a control scenario where participants were exposed to filtered air (FA) with no O_3_.

**Table 1:** Selected observational and experimental studies of ozone exposure and pulmonary function

Author (year)	Study Region/year	Study Population	Exposure Metrix	Health Outcome	Effect Estimates
Experimental Studies					
Kleinman et al. (1981) [31]	Southern California	Adults (*n* = 19)	clean air or a mixture of 0.37ppm O_3_, 0.37ppm SO_2_, 100ug/m^3^ H_2_SO_4_ /2hr/2 separate days	FVC, FEV_1_, TLC, RV, CC/TLC, Rt, symptoms	↓FVC, ↓FEV_1_, ↓FEV_2_, ↓FEV_3_, ↓MMFR, ↓Vmax_50_, ↓ Vmax_25_, ↑symptom, but no larger effects were seen in mixed exposure
Bedi et al. (1982) [34]	Santa Barbara	Male adults (*n* = 8)	0, 0.4ppm O_3_/0.4ppm SO_2_/2hr at 35–85%RH	FVC, ERV, FEV_1,2,3_, FEF_50%,_ FEF_75%,_ FEF_25−75%,_ FRC, TGV, Raw	↓FEV_1_, ↓ERV, ↓TLC, ↓FEV_25−75_, ↑TGV, but no synergistic effects with co-exposed to SO_2_
Drechsler-Parks et al. (1984) [32]	Santa Barbara	Male adults (*n* = 10)	(1) filtered air, (2) 0.30ppm PAN, (3) 0.45 ppm O_3_, (4) 0.30ppm PAN + 0.45 ppm O_3_ /2-hour	FRC, FVC, VE, VO_2_, Vt, fR, FEV_1,2,3,_ FEF_25−75_, IC, ERV, TLC	↓FVC, ↓FEV_10_, ↓FEV_20_, ↓FEV_30_, ↓ FEF_25−75%_, ↓IC, ↓ERV, ↓TLC; larger effects when co-exposed to PAN
Gibbons and Adams (1984) [33]	Davis	Female adults (*n* = 10)	0, 0.15 0.3ppm/24°C, 35 °C 1 h	FVC, FEV_1_, FEF_25−75_, TLC	↓FEV_1_, ↓FVC, with greater decrements with higher temperature
Aris et al. (1993) [29]	San Francisco	Adults (*n* = 10)	500ugg/m3 HNO_3_, 0.2ppm O_3_/4hr/2-week apart	FEV_1_, FVC, SRaw, BalF, symptom	↓FEV_1_, ↓FVC, ↑SRaw, ↑total cell, ↑neutrophils,↑symptom; no difference between exposures to HNO_3_/O_3_ and O_3_ alone
McDonnell et al. (1993) [12]	North Carolina	Male adults (*n* = 290)	0, 0.12, 0.18, 0.24, 0.30, or 0.40 ppm/2hr	FEV_1_, PEF, FVC	↓FEV_1_ with greater decrement in younger age group
Linn et al. (1994) [31]	Southern California	Adults (*n* = 45)	100ug/m3 H_2_S0_4_, 0.12ppm O_3_ /6.5 h/2days	FEV_1_, FVC, SRaw, PEFR	↓FEV_1_, ↓FVC, ↑SRaw, ↓PEFR with greater changes when exposed to combined H2SO4 /O_3_,
Weinmann et al. (1995) [13]	Baltimore	Male adults (*n* = 64)	0.35ppm/130–150 min	FVC, FEV_1_, FEF_25− 75_, symptoms	↓FVC, ↓FEV_1_, ↓FEV_1_/FVC, ↓FEF_25−75_; the decrement in FVC was not correlated with FEF_25−75_ changes, but weakly related to f and VT
Gong et al. (1997) [36]	Downey	Male adults (9 with COPD, 10 healthy)	0.24ppm/4hr/1w-apart	FVC, FEV_1_	% change: < COPD> FEV_1_=-19.2%, FVC_min=-23%; < Healthy> FEV_1_=-1.9%, FVC_min=-4%
Torres et al. (1997) [24]	Rochester	Adults (*n* = 90)	0.22 ppm/4 hr	FVC, FEV_1_, and FEF_25–75%,_ BalF	No significant decrements in lung function; ↑PMN (peaked at 18 h; *p* < 0.0001); ↑IL-6 (> 10-fold early; *p* < 0.0001); ↑IL-8 (early increase; *p* < 0.0001)
Samet et al. (2001) [99]	University of North Carolina at Chapel Hill	Adults (*n* = 31)	0, 0.4ppm/2hr; placebo or 250 mg vitC, 50 IU α -tocopherol, and 12 oz vegetable cocktail daily/2 week	FEV_1_, FVC, BAL	↓ FEV_1_ and FVC were 30% and 24% smaller among those with supplements compared to placebo group
Adams (2003) [22]	Davis	Adults (*n* = 30)	0.3ppm/2hr or 0.08ppm/6.6 h	FEV_1_, FVC	< 6.6 h 0.08 ppm > FEV_1_ −3.51%, FVC − 3.67%, FEV_1_/FVC + 0.16%, TSS 8.2; <2 h 0.30 ppm > FEV_1_ −12.36%, FVC − 11.53%, FEV_1_/FVC − 0.93%, TSS 30.9; <2 h 0.30 ppm (face mask)> FEV_1_ −11.37%, FVC − 10.49%, FEV_1_/FVC + 1.06%, TSS 30.0.
Adams (2006) [21]	Davis	Adults (*n* = 30)	0.08ppm square-wave and 0.08ppm triangle; 0.06ppm square-wave and 0.06ppm triangle/6.6 h	FEV_1_, FVC, total symptom severity (TSS)	< FEV_1_ (%Δ post-6.6 h) > FA + 1.35; 0.04 ppm (tri) + 1.17; 0.06 ppm (sq) − 1.51; 0.06 ppm (tri) − 1.43; 0.08 ppm (sq) − 4.72 (significant at 6.6 h); 0.08 ppm (tri) − 5.65 (significant from 4.6 h); < FVC (%Δ post-6.6 h) > FA − 0.44; 0.04 ppm (tri) − 0.74; 0.06 ppm (sq) − 0.89; 0.06 ppm (tri) − 1.72; 0.08 ppm (sq) − 4.46; 0.08 ppm (tri) − 4.78; < FEV_1_/FVC (%Δ post-6.6 h) > FA + 0.92; 0.04 ppm (tri) + 0.43; 0.06 ppm (sq) − 0.62; 0.06 ppm (tri) + 0.31; 0.08 ppm (sq) − 0.36; 0.08 ppm (tri) − 1.00; < Total symptom score (end-exposure) > FA 0.6; 0.04 ppm (tri) 2.0; 0.06 ppm (sq) 2.5; 0.06 ppm (tri) 3.9; 0.08 ppm (sq) 9.6; 0.08 ppm (tri) 8.1
Arjomandi et al. (2008) [17]	Berkeley	Adults (*n* = 15)	200ppb/4hr and life-time cumulative exposure	FVC, FEV_1_, FEF_25−75_, FEF_75_, symptoms, BalF, Clara cell protein (CC16)	< FEV_1_ (Δ across exposure) > − 0.4 L (− 0.3 to − 0.7); < FVC > − 0.5 L (− 0.2 to − 0.6), *p* < 0.001; < FEF_25–75_ > − 0.5 L/s (− 0.2 to − 1.2); < FEF_75_ > − 0.3 L/s (− 0.1 to − 0.4), *p* = 0.006; < Lower respiratory symptoms > + 1.15 (0.7–1.3); < Upper respiratory symptoms > + 0.25 (0.0–1.5); < Plasma CC16 > + 1.8 ng/mL at 18 h (− 0.2 to + 3.8); immediate post: +6.1 ng/mL
Kim et al. (2011) [27]	North Carolina	Adults (*n* = 59)	0, 0.06pcpm/6hr	FEV_1_, FVC, PMN, symptoms	< FEV_1_ (% change after 6.6 h) > CA 0.00 ± 0.46, O_3_ −1.71 ± 0.50; CA-adjusted Δ − 1.71 ± 0.64 (*p* = 0.008); by sex—M: −1.45 ± 0.95 (*p* = 0.14), F: −1.93 ± 0.88 (*p* = 0.02); by GSTM1—positive: −1.99 ± 0.90 (*p* < 0.05), null: −1.42 ± 0.92 (p = NS). < FVC (% change after 6.6 h) > CA − 1.13 ± 0.34, O_3_ − 2.32 ± 0.41; CA-adjusted Δ − 1.19 ± 0.51 (*p* = 0.02); by sex—M: −0.49 ± 0.74 (p = NS), F: −1.78 ± 0.68 (*p* < 0.05); by GSTM1—positive: −1.30 ± 0.73 (*p* = 0.07), null: −1.07 ± 0.71 (p = NS).
Kahle et al. (2015) [35]	University of North Carolina at Chapel Hill	Adults (*n* = 16)	0, 0.3ppm/22°C, 32.5 °C 2 h	FVC, FEV_1_, IL-1β, IL-6, IL-8, and TNF-α, CRP	FEV_1_ (T = 22 °C): -12.4% (95% CI: -17.0%- -6.1%); FEV_1_ (T = 32.5 °C): -7.5% (95% CI: -13.1%--2.3%); No effect modification of temperature on O_3_ on FVC and FEV_1_ observed; No significant changes were observed for IL-6, IL-8, and TNFα
Bennett et al. (2016) [37]	North Carolina	Adults (*n* = 40)	0, 0.4ppm/2hr/separate by 2w-6 m	FEV_1_, FVC, IC, Sgaw, inflammation	delta % fall: < FVC> Obese: 12.5 (7.5), Normal: 8.0 (5.8); < FEV_1_> Obese: 15.9 (8.6); Normal: 11.7 (7.1), < IC> Obese: 16.7 (14.2), Normal: 10.2 (13.2); < sGaw> Obese: 12.6 (26.2), Normal: 12.4 (21.0); Concentration (pg/mL): < IL-6 > Obese: 13.1 (3.0, 24.4), Normal: 15.9 (6.7, 33.3)
Frampton et al. (2017) [18]	Rochester, North Carolina, San Francisco	Adults (*n* = 87)	0, 70, 120ppb/3hr/15 mins exercise with 15 min of rest	FVC, FEV_1_, FEV_1_/FVC, FEF_25–75,_ inflammation factors in sputum and blood	↓FVC, ↓FEV_1_: +85 mL (64 mL, 106 mL), ↓FEV_1_/FVC, ↓FEF_25−75_, ↑club cell 16 (blood), ↑neutrophils (sputum), no difference across sex, age, or GSTM1 status groups
Chen et al. (2022) [100]	University of North Carolina at Chapel Hill	Adults (*n* = 43)	0, 0.3ppm/2 h; 3 g/day of fish oil, 3 g/day olive oil, or no supplementation/4week	FVC, FEV_1_, FEV_1_/FVC, sputum neutrophil percentage	↓ FEV_1_ and FEV_1_/FVC were 48% and 70% smaller among those with fish oil supplements compared to control group
Pennington et al. (2026) [28]	North Carolina	Adults (*n* = 38)	0.00, 0.07ppm/6.6 h	FEV_1_, FVC, Respiratory total symptom scores (TSS)	FEV_1_(mean ± SEM): −1.24 ± 0.92%; FVC (mean ± SEM): −1.22 ± 0.29%; TSS (mean ± SEM): 1.0 ± 0.3
Observational studies					
Spektor et al. (1988) [46]	New Jersey (Fairview Lake) /1988	Children (*n* = 46)	1 h and 24 h- average level before measurement (Not specified)	FEV_1_, FVC, PEFR, FEF_25−75_	< FVC > − 1.03 ± 0.24 ml/ppb; < FEV₁> −1.42 ± 0.17 ml/ppb; < PEFR > − 6.78 ± 0.73 ml/s/ppb; < FEF₂₅–₇₅> −2.48 ± 0.26 ml/s/ppb (all *p* < 0.05); 1-h O_3_ strongest predictor; predicted decrements at 120 ppb: 4.9%, 7.7%, 17%, 11% (FVC, FEV₁, PEFR, FEF_25−75_).
Schwartz (1989) [11]	U.S.A./1976–1980	NHANES II subjects(*n* = 20,322)	Annual average level (Not specified)	FVC, FEV_1_, PEF	FVC: β=-3.20 (*p* = 0.0001), FEV_1_: β=-3.19 (*p* = 0.0001), PEF: β=-4.15 (*p* = 0.0001)
Higgins et al. (1990) [47]	California (Bernardino mountains)/1987	Children (*n* = 43)	Daily average level (Not specified)	FEV_1_, FVC, PEFR	Morning (07:00–11:00): < FEV₁> 0.90 (± 0.62; R² = 0.97), < FVC > 0.35 (± 0.67; R² = 0.97), < PEFR > 4.10 (± 2.66; R² = 0.89). Midday (11:00–15:00): < FEV₁> −0.20 (± 0.23; R² = 0.96), < FVC > − 0.23 (± 0.29; R² = 0.95), < PEFR > 1.23 (± 1.22; R² = 0.85). Afternoon (15:00–19:00): < FEV₁> −0.73* (± 0.28; R² = 0.97), < FVC > − 0.91† (± 0.34; R² = 0.97), < PEFR > − 0.42 (± 1.03; R² = 0.92).
Kinney et al. (1996) [48]	New Jerey, Ontario, Southern California /1983–1988	Children (*n* = 616)	previous 1-hr average level (Not specified)	FEV_1_, PEFR	< FEV₁> slope − 0.50 ml/ppb (SE 0.07, *p* = 0.0001); < PEFR> slope + 0.17 ml/s/ppb (SE 0.33, *p* = 0.62).
Abbey et al. (1998) [38]	California/1977–1992	Adults (*n* = 1,391)	Yearly average level (IQR: 23 ppb)	PPFEV_1_, FEV_1_/VC, PEF lability	% predicted FEV_1_ in males=-6.3% (-10.8%, -1.8%) whose parents had asthma, bronchitis, emphysema, or hay fever; Others not specified
Korrick et al. (1998) [14]	Pinkham Notch (New Hampshire)/Not specified	Adults (*n* = 595)	hourly average (50 ppb)	FEV_1_, FVC, FEV_1_/FVC, FEF_25−75_, PEER	% change: FEV₁ −0.051 ± 0.022 (*p* = 0.02); FVC − 0.043 ± 0.014 (*p* = 0.003); PEFR − 0.018 ± 0.058 (*p* = 0.76); FEV₁/FVC − 0.009 ± 0.018 (*p* = 0.61); FEF₂₅–₇₅ −0.027 ± 0.070 (*p* = 0.70)
Gauderman et al. (2002) [15]	Southern California/1996–2000	Children (*n* = 1,678)	Annual average (8 h: 36.6 ppb, overall: 39.8 ppb)	FVC, FEV_1_, MMEF, MMEF/FVC, PEFR	10 a.m.–6 p.m.: FVC − 0.33% (− 0.90, 0.24); FEV₁ −0.55% (− 1.27, 0.16); MMEF − 0.80% (− 1.94, 0.36); MMEF/FVC − 0.44% (− 1.39, 0.52); PEFR − 1.21% (− 2.06, − 0.36)
Ross et al. (2002) [45]	Illinois/1994	Children and adults (*n* = 40)	Daily average 8 h maximum level with lag days (not specified)	PEFRs, respiratory symptoms, frequency of asthma attacks, and asthma medication use	Not specified; ↓PEFR, ↓asthmatic medication use, ↑morning symptoms
Gauderman et al. (2004) [41]	Southern California/1993–2001	Children (*n* = 1,759)	8-year average of 8-h (37.5 ppb) and 1-h max levels (46.0 ppb)	FEV_1_, FVC, MMEF	10 a.m.–6 p.m.: FVC − 50.6 mL (− 171.0, 69.7); FEV₁ −22.8 mL (− 122.3, 76.6); MMEF + 85.6 mL/s (− 130.0, 301.1) | 1-h max: FVC − 70.3 mL (− 183.3, 42.6); FEV₁ −44.5 mL (− 138.9, 50.0); MMEF + 45.7 mL/s (− 172.3, 263.6)
Goss et al. (2004) [43]	U.S.A./1999–2000	Children and adults with cystic fibrosis (*n* = 11,484)	Annual average level (10 ppb)	FEV_1_, CF-related pulmonary condition exacerbation	Pulmonary exacerbation: OR = 1.10 (1.03, 1.17), FEV1: no association was observed
Wang et al. (2019) [19]	6 U.S. metropolitan areas/2000–2002	Adults (*n* = 7,071)	10-year average exposure (3 ppb)	Progression of percent emphysema shown on CT imaging, FEV_1_, FVC, FEV_1_/FVC	FEV_1_: -18.15 mL (-34.71 mL, -1.59 mL), FVC: -40.19 mL (-62.49 mL, -17.88 mL), FEV_1_/FVC: No association, Change in % emphysema: Baseline concentration = 0.13 (0.03, 0.24), During follow-up exposures = 0.18 (0.08, 0.28)
Tong et al. (2022) [101]	University of North Carolina at Chapel Hill/2016–2019	Adults (*n* = 62)	daily maximum 8-hour (17ppb) / Polyunsaturated omega 3 fatty acids (n-3 FA) and n-6 FA	FEV_1_, FVC	Lag 0: FVC: ↑1.8% (95% CI: 0.5%-3.2%) with high n-3 FA vs. 0.0% [95% CI: -1.4%-1.5%) with low n-3 FA group, ↑1.7% (95% CI: 0.3%-3.0%) with high n-6 FA vs. 0.5% (95% CI: 20.9%-2.0%) with low n-6 FA; Lag 4: ↓1.9% [95% CI: -3.2%- -0.5%) with high n-3 FA vs.↑0.2% (95% CI: -1.2%- 1.5%) with low n-3 FA, ↓1.4% (95% CI: -2.8%-0.0%) with high n-6 FA vs. -0.5% [95% CI: -1.8%-0.9%) with low n-6 FA.
Hsu et al. (2023) [20]	Boston/2001–2008	Mother-child (*n* = 198 pairs)	Prenatal weekly-average level (SD: 2.84 ppb)	FVC, FEV_1_, FEF25-75%	FEV_1_/FVC was negatively associated with 18–22-week gestation ozone exposure
Lu et al. (2025) [50]	Fresno, California/2015–2019	Children (*n* = 229)	1-week, 1-month, 3-month, 6-month, and 12-month periods before each visit	FEV_1_, FVC, and FEV_1_/FVC	1-week: < FEV₁> -0.05 (95% CI: -0.30–0.20), < FVC> -0.25 (95% CI: -0.50–0.10),< FEV₁/FVC> -0.01 (95% CI: -0.40–0.25); 1-month: < FEV₁> +0.01 (95% CI: -0.30–0.30), < FVC> -0.04 (95% CI: -0.45–0.25), < FEV₁/FVC> -0.04 (95% CI: -0.50–0.40);3-month: < FEV₁> -0.13 (95% CI: -0.50–0.20), < FVC> -0.03 (95% CI: -0.40–0.30), < FEV₁/FVC> -0.20 (95% CI: -0.60–0.20); 6-month: < FEV₁> -0.25 (95% CI: -0.60–0.10),< FVC> -0.04 (95% CI: -0.40–0.20), < FEV₁/FVC> -0.16 (95% CI: -0.50–0.25);1-year: < FEV₁> -0.04 (95% CI: -0.13–0.05), < FVC> -0.01 (95% CI: -0.20–0.20),< FEV₁/FVC> -0.05 (95% CI: -0.25–0.20).
Zetlen et al. (2025) [45]	East Massachusetts/Not specified	Children (*n* = 2,128)	Average exposure during the first year of life, birth through the midchildhood visit, and birth through the early teen visit	FEV_1_, FVC, FeNO	Midchildhood: < FEV_1_> -2.5 (95% CI: -29.0, 24.1) and < FVC> -9.0 (95% CI: -37.7, 19.7) for O_3_ in first year of life; < FEV_1_>-4.6 (95% CI: -18.2, 9.0) and < FVC> -10.2 (95% CI: -24.9, 4.5) for O_3_ through midchildhood; Early Teen: < FEV_1_> -1.9 (95% CI: -42.5,38.7) and < FVC > + 1.2 (95% CI: -42.3, 44.7) and < FeNO > 3.8 (95% CI: -4.2-12.5) for O_3_ in first year of life; < FEV_1_ > + 1.7 (95% CI: -20.3, 23.7) and < FVC> +9.6 (95% CI: -13.7-33.0) and < FeNO > 2.2 (95% CI: -2.1-6.7) for O_3_ through midchildhood; < FEV_1_ > + 2.2(95% CI: -18.2-22.5) and < FVC> +4.8 (95% CI: -17.0-26.6) and < FeNO > 0.3 (95% CI: -3.6-4.3) for O_3_ through early teens; Midteen: < FEV_1_> -18.4 (95% CI: -77.4-40.6 ) and < FVC> -21.1 (95% CI: -87.8-45.5) and < FeNO > 4.4 (95% CI: -4.2-13.9) for O_3_ in first year of life; < FEV_1_ > 0.0 (95% CI: -33.7-33.6) and < FVC> +1.8 (95% CI: -36.3-40.0) and < FeNO > 2.4 (95% CI: -2.6-7.6) for O_3_ through midchildhood; < FEV_1_> +-4.2(95% CI: -34.4-26.0) and < FVC> -2.0 (95% CI: -36.4,32.3) and < FeNO > 1.5 (95% CI: -2.9-6.1) for O_3_ through early teens.

This table summarizes the subset of studies discussed in the main text. The remaining studies reviewed are available in supplementary Table S1

Adverse lung function decrements have been consistently observed with controlled exposures to O_3_ of at least 0.08-ppm (Table 1). In a 1986 University of California (UC) Davis study with 10 highly trained endurance athletes, the participants were randomly exposed to FA, and 0.12-ppm, 0.18-ppm or 0.24-ppm O_3_ while performing 1-hour competitive exercise [23]. The researchers observed that 0.18- and 0.24-ppm exposures during one hour of heavy exercise were associated with FEV₁ reductions of 5.8–10.5% and FVC reductions of 7.8–9.9%, respectively. Similarly, in another study at the UC Berkeley consisting of 15 healthy young adult students, it was reported that O_3_ exposure was associated with a 0.5 L, 0.4 L, 0.5 L/s and 0.3 L/s decrease in FVC, FEV_1,_ FEF_25−75,_ FEF_75,_ respectively [17]. In a study conducted at the University of Rochester, participants were exposed to 0.22-ppm O_3_ for 4 h, with exercise for 20 of every 30 min. Markers of airway inflammation, measured in bronchoalveolar lavage fluid (BALF), specifically IL-6 and IL-8 were observed to be increased by up to 10-fold and 2-fold, respectively [24]. Similarly, in a Michigan study which exposed 10 adults (5 asthmatics and 5 healthy) to 0.2-ppm O_3_ observed significant increases in IL-6, IL-8 and other airway inflammatory indicators measured in BALF, but no difference was seen between baseline and postexposure FEV_1_, FVC, FEV_1_/FVC and FEF_25−75_, suggesting that airway inflammatory responses can occur independent of pulmonary function deficits [25].

As the U.S. Environmental Protection Agency (EPA) continues to lower the primary O_3_ NAAQS, recent studies have focused on environmentally relevant concentrations to inform regulatory thresholds (Table 1). In a UC Davis study, researchers examined the lung function effects of time-dependent chamber exposures to O_3_ for 6.6 h in 30 healthy, nonsmoking young adults using six different experimental protocols: standard square-wave concentration profiles at 0-, 60- and 80-ppb O_3_ and triangular concentration profiles at 40, 60 and 80-ppb O_3_ [21]. Decrements in lung function with only 80-ppb exposure protocols were observed. Specifically, the average decrement in FEV_1_ was 6.1% and 7.0% relative to FA for the square-wave and triangular 80-ppb O_3_ exposures, respectively. A dose-dependent average decrement in FVC was also observed, with maximum FVC change of 4.9% for square-wave and 5.2% for triangular protocols at 80-ppb O_3_ relative to FA [26]. In another study, the researchers exposed 59 healthy young adults (19–35 years) to 0, and 0.06-ppm O_3_ for 6.6 h and found that the participants experienced a greater change in FEV_1_ and FVC immediately after exposure to 0.06-ppm O_3_ compared with FA (FEV_1_: -1.71 vs. -0.002; FVC: -2.32 vs. -1.13) [27]. Additionally, in a recent study conducted at the University of North Carolina, 38 healthy young adults aged 19–34 years without a history of smoking were exposed to clean air (CA) and 0.07-ppm O₃ for 6.6 h with intermittent moderate exercise. A decrease in FEV₁ was observed immediately after exposure to 0.07-ppm O₃ compared with CA (− 1.24% vs. 0.83%) [28].

Considering that human exposure studies with single O_3_ exposure may underestimate potential toxicity if significant synergy occurs between O_3_ and other coexisting pollutants, researchers also added several common pollutants to the experimental studies [29–31] (Table 1). In a study with 19 healthy volunteers, researchers from the University of Southern California reported that the participants exposed to O_3_ at 0.37-ppm in the presence of 100 µg/m^3^ of sulfate aerosol (H_2_SO_4_) and 0.37-ppm sulfur dioxide (SO_2_) exhibited 3.7% decrements in FEV_1_ [30]. This finding was corroborated with another study evaluating effects of “acid summer haze” on individuals who exercise extensively outdoors [31]. In the study, 45 adult volunteers were exposed to a mixture of 0.12-ppm O_3_ and approximately 100 µg/m^3^ of H_2_SO_4_. Similar as the previous study, exposure to O_3_ alone or O_3_ and H_2_SO_4_ mixture resulted in a FVC reduction, although the difference was not statistically significant. In another UC Santa Barbara study, 10 young adult men were exposed to 0.45-ppm O_3_ and 0.30-ppm peroxyacetyl nitrate (PAN) separately or combined. FEV_1_ was significantly decreased 20.8% after being exposed to O_3_ alone for 75 min, and the PAN/O_3_ exposure induced averaging approximately 10% greater pulmonary function decrements than that was observed during exposure to O_3_ alone, indicating an interaction effect between PAN and O_3_ [32].

Exacerbation of pulmonary function impairment induced by O_3_ exposure during exercise was also reported (Table 1). Exercise may increase minute ventilation, which may potentiate responses to O_3_ by increasing the absolute amount inhaled and promoting more uniform ventilation. In controlled exposure studies, exercise can also be used to increase the total delivered O_3_ dose to better approximate exposures over an 8-hour workday. and may help normalize the delivered dose across participants despite differences in resting ventilation and lung size. Additionally, warmer temperatures usually may accelerate the chemical reactions that generate O_3_ and stimulate increased expired ventilation (VE) during exercise [33]. In a UC Davis study, 10 aerobically trained young adult women were recruited and exercised continuously with exposure to FA, and 0.15-ppm and 0.30-ppm O_3_ in both moderate (24 °C) and hot (35 °C) ambient conditions [33]. A significant interaction of O_3_ and ambient heat was observed for FVC and FEV_1_, as well as for respiratory frequency and alveolar volume. Another study in Santa Barbara exposed 8 young healthy males to 0.4-ppm O_3_ and 0.4-ppm SO_2_ at 35 °C-85% relative humidity for 2 h and found that compared to 25 °C-50% relative humidity functional residual capacity (FRC) increased significantly and FEF_25−75_ exhibited larger decreases in higher temperature environment [33, 34]. These studies indicate that O_3_ exposure coinciding with ambient heat is likely to result in more severe physiological alterations in lung function. In contrast, another study in University of North Carolina, researchers found that O_3_-induced deduction in FEV_1_ were comparable between moderate (22 °C) and elevated (32.5 °C) temperature conditions (FEV_1_: − 12.4% vs. − 7.5%), while no significant temperature-related modification of the reductions in lung function was observed [35].

Individuals with pre-existing conditions such as chronic obstructive pulmonary disease (COPD) and obesity are considered more sensitive to O_3_ exposure compared to the clinically healthy group (Table 1). In a study consisting of 9 men who had severe COPD and 10 age-matched healthy men, participants were exposed to 0.24-ppm O_3_ and to FA for 4 h in a random order with 1-week apart. The average decrement in FEV_1_ among the COPD groups was 19% after exposure to O_3_, compared with 2% in the healthy group (*p* < 0.001) [36]. In another University of North Carolina study, the researchers recruited 20 obese and 20 normal weight females to examine whether obesity enhances O_3_-induced pulmonary dysfunction [37]. Although both groups showed significant decrements in FVC and FEV_1_ after O_3_ exposure, the decrement was significantly larger among the obese group (FVC: -12.5%; FEV_1_: -15.9%) compared to normal weigh group (FVC: -8.0%; FEV_1_: -11.7%).

Collectively, experimental studies have yielded relatively consistent results, demonstrating that O_3_ exposure―even at or near current regulatory levels―can lead to measurable lung dysfunction and increased inflammation. These impacts could be enhanced by co-exposure to other pollutants, ambient heat and humidity, physical activity, especially among vulnerable populations.

### Observational Studies

Besides experimental studies, a large body of observational studies including longitudinal and cross-sectional studies has been conducted to evaluate the effects of O_3_ exposure on pulmonary function (Table 1 and Table S1). These studies are critical for understanding real-world health impacts, as they assess individuals under ambient conditions, capture diverse exposure histories, and reflect population-level variation in susceptibility and behavior. While cross-sectional studies assess associations between current lung function and long-term or cumulative exposure, longitudinal studies track within-individual changes over time. These studies complement experimental and semi-controlled investigations by evaluating O_3_-health associations in broader, more representative populations under ambient exposure conditions. By capturing long-term or repeated exposures, and short-term fluctuations in air quality, observational studies offer valuable insights into the population-level burden of O_3_-related pulmonary impairment.

Cross-sectional studies were often used to assess O_3_-related respiratory functions, assuming that current air pollution exposure is highly correlated with previous exposure to make a plausible link with current health status [38]. In a study with participants obtained from the Adventist Health Study of Smog, lifetime O_3_ exposure was estimated from 20-year residential histories [39]. For each 23-ppb increase in annual average O_3_, FEV₁ was found to decrease by 6.3% in males whose parents had asthma, bronchitis, emphysema, or hay fever (Table 1). Another study using data from the Second National Health and Nutrition Examination Survey (NHANES) involving 4,300 individuals aged 6–24 years also reported negative associations between annual O_3_ concentrations with both FEV₁ and FVC [11]. In a study involving 10,251 children aged 8–12 years, the researchers found that higher 3-year average O_3_ concentrations were statistically significantly associated with reduced FEV₁, FVC, FEV₀.₇₅, FEF₂₅-₇₅, and PEFR (Table 1) [40].

While cross-sectional studies offer valuable insight into population-level effects, longitudinal studies allow for tracking pulmonary changes over time (Table 1). Using the data from Children’s Health Study consisting of 1,759 children aged 12–18 years in 12 Southern California communities, no statistically significant differences were found in growth rates for all the pulmonary function tests including FVC, FEV_1_, FEF_75_, or MMEF for each 38.6-ppb or 55-ppb increase in annual average 8-hour maximum and hourly O_3_ concentration [41, 42], while it was reported that each 36.6-ppb increase in 8-hour maximum O_3_ was associated with a − 1.21% reduction in PEF growth [15]. Although most studies consistently reported a negative association between long-term O₃ exposure and lung function, some studies reported inconsistent findings. A longitudinal study of cystic fibrosis patients, in which O_3_ exposure was assigned based on nearest air monitors, found no relationship between annual average O_3_ and lung function decline [43]. These discrepancies may reflect study-specific factors such as limited statistical power due to small sample size, reduced sensitivity of spirometric endpoints to detect small airway changes in cystic fibrosis patients, or exposure misclassification stemming from assigning ambient O_3_ levels based solely on residential proximity rather than personal exposure metrics. In addition to investigating the effects of long-term O_3_ exposure, longitudinal studies have also been employed to assess the impacts of short-term exposure. In a 10-year study of 904 older men from the Normative Aging Study, a 15-ppb increase in 48-hour average O_3_ was associated with declines in FEV_1_ (− 1.29%) and FVC (− 1.36%) [44]. In another study of 40 asthmatic adults living near East Moline, Illinois, same-day 8-hour maximum O_3_ exposure was associated with 2.29% and 2.58% reductions in morning and evening PEFR, respectively [45].

Although the bulk of the data derived from experimental studies have revealed a spectrum of O_3_ effects on the human respiratory system, relatively few data are available on children, the subgroup that may be at higher risk of O_3_-induced health impacts (Table 1 and Table S1). Unlike adults, children cannot be enrolled in experimental studies for O_3_, due to ethical and regulatory restrictions. As a result, real-world observational settings such as summer camps offer a rare and valuable alternative to assess ambient O_3_ exposure and respiratory outcomes in pediatric populations. These camp-based studies, also called camp studies, recruit school aged children and monitor their lung function, typically through repeated daily measurements of FEV₁, FVC, and PEFR, while simultaneously tracking ambient air pollutants and meteorological variables. In a study of 91 children aged 8–11 in New Jersey, investigators observed statistically significant decreases in FEV₁, FVC, and PEFR associated with 1-, 2-, and 4-hour O_3_-averages prior to spirometric measurements [46], and negative associations persisted when analyses were restricted to days with O_3_ levels below 60-ppb or 80-ppb, suggesting that exposures even lower than regulatory criteria may elicit adverse effects. Similarly, a study conducted in California’s San Bernardino Mountains found that each 1-ppb increase in daily average O_3_ was associated with a 0.39-ml reduction in FEV₁ and a 0.44-ml reduction in FVC [47]. These findings align with a pooled analysis by Columbia University researchers [48], which reported that each 1-ppb increase in previous-hour O_3_ concentration was associated with a 0.50-ml reduction in FEV₁, indicating that children exposed to O_3_ under natural conditions experience decreases in FEV_1_. A recent study recruited the children (*n* = 2,128) in the Boston-area from Project Viva measured FEV_1_ and FVC at mid-childhood, early-teens and mid-teens study visits, and F_e_NO at the early- and mid-teens study visits, and reported that O_3_ exposure during the first year of life was associated with slower lung function growth between early and mid-adolescence, while no consistent associations between long-term O_3_ exposure and FEV₁ or FVC from mid-childhood through adolescence were observed [49]. Another recent study in Fresno, California, followed 299 children at approximately 7 and 9 years of age and examined residential exposure to O_3_ over multiple exposure windows (1 week―12 months). O_3_ exposure was generally associated with lower FEV₁, FVC, and FEV₁/FVC ratio across all the exposure windows; however, all 95% confidence intervals included the null [50].

Overall, observational studies provide biologically plausible evidence that O_3_ exposures contribute to pulmonary function impairments across different populations, with relatively consistent findings for short-term exposures but more mixed evidence regarding the effects of long-term exposure. The convergence of findings across study designs strengthens the epidemiological basis for O_3_ regulation and reinforces experimental evidence on its respiratory effects.

## Asthma-Related Symptoms, ED Visits/Hospitalizations

Asthma, as a chronic respiratory disorder, is a nationwide public health concern affecting nearly 28 million people in the U.S [51]. O_3_ exposure has been implicated in the exacerbation of asthma, often resulting in ED visits/hospital admissions—key indicators of asthma severity and disease burden. In evaluating existing research, it is important to distinguish between short-term exposure—referring to transient spikes over several hours or days—and long-term exposure, which captures sustained ambient O_3_ levels over extended periods. The subsections present key collective studies that illustrate the magnitude and consistency of these associations across varying timescales and population groups (Table 2 and Table S2).

**Table 2:** Selected studies of ozone exposure and asthma-related symptoms, ED visits and hospitalizations

Author (year)	Study Region/year	Study Population	Exposure Metrix (per unit increase)	Health Outcome	Effect Estimate
Asthma-related Symptoms					
McDonnell et al. (1999) [52]	California/1973–1992	Adults (Seventh-day Adventist) (*n* = 3,091)	20-year average level (27 ppb)	Asthma onset	Male: OR = 2.09 (1.03, 4.16), Female: OR = 0.86 (0.58, 1.26)
McConnell et al. (2002) [9]	Southern California/1993–1998	Children (*n* = 3,535)	Number of sports played in low/high ozone polluted communities	New onset asthma risk	High ozone communities with three or more sports played: RR = 3.3 (1.9, 5.8), Low ozone communities with three or more sports played: RR = 0.8 (0.4, 1.6)
Meng et al. (2007) [55]	Los Angeles, San Diego/2000–2001	Adults with asthma (CHIS data) (*n* = 1,609)	Yearly average level (10ppb)	Poorly controlled asthma risk	All adults: OR = 1.08 (0.84, 1.39), Nonelderly adults: 1.00 (0.76, 1.31), Elderly: OR = 1.70 (0.91, 3.18), Men: OR = 1.76 (1.05, 2.94), Women: OR = 0.90 (0.67, 1.19)
Wilhelm et al. (2008) [54]	California/2000–2001	Children with asthma (CHIS data) (*n* = 597)	Yearly average level (10 ppb)	Asthma daily/weekly symptoms	OR = 2.09 (1.28, 3.41)
Akinbami et al. (2010) [56]	US metropolitans /2001–2004	Children (*n* = 34,073)	Yearly average level (5 ppb)	Current asthma, Asthma attacks	Current asthma: OR = 1.08 (1.02, 1.14), Asthma attack: OR = 1.07 (1.00, 1.13)
Meng et al. (2010) [53]	San Joaquin Valley/2000–2001	Residents with asthma (*n* = 1,487)	Yearly average level (10 ppb)	Asthma symptoms	OR = 1.23 (0.94, 1.60)
Lewis et al. (2013) [59]	Detroit/1999–2002	Children with asthma (*n* = 298)	Daily 1-hr maximum level (16 ppb)	Asthma symptoms (Wheezing)	OR = 1.118 (1.013, 1.233)
Fishe et al. (2022) [60]	Florida/2012–2017	OneFlorida patient’s database (*n* = 533,446)	Daily average level (Q1: ≤31.1 ppb, Q2: 31.1–36.4 ppb, Q3: 36.4–42.0 ppb, Q4: ≥42.0 ppb)	Asthma exacerbation	Q2: OR = 1.01 (1.00, 1.03), Q3: OR = 1.06 (1.05, 1.07), Q4: OR = 1.07 (1.05, 1.08)
Dearborn et al. (2023) [57]	6 U.S. cities/2006–2020	Children with bronchiolitis (*n* = 224)	2-year average level (2 ppb)	Current asthma, current wheeze	Current asthma: RR = 1.4 (1.1, 1.8), Current wheeze: RR = 1.3 (1.0, 1.7)
Su et al. (2024) [58]	California/2012–2019	People with asthma or COPD (*n* = 3,386)	Daily maximum 8-hr average level (1 ppb, 30 ppb)	Daily SABA use (related to asthma symptoms)	Single pollutant model_1ppb: IRR = 1.100 (0.999, 1.001), Single pollutant model_30ppb: IRR = 1.000 (0.970, 1.030), Multi pollutant model_1ppb: IRR = 1.005 (1.004, 1.007), Multipollutant model_30ppb: IRR = 1.161 (1.127, 1.233)
Asthma-related ED visits and Hospitalizations					
Nauenberg et al. (1999) [67]	Los Angeles/1991–1994	Hospital discharge data (*n* = 11,240)	24-hour average level, 8-day average level (50 ppb)	Asthma hospital admissions	Same day: RR = 1.01 (0.98, 1.05), 8-day average: RR = 1.02 (0.97, 1.07)
Babin et al. (2007) [63]	Washington DC/2001–2004	ED visits data (Not specified, Data of 1,096 days)	Daily maximum 8-hr average (0.01 ppm)	Asthma ED visits, Hospitalizations	ED visits: RR = 1.032 (1.4, 5.0), Admissions: 1.083 (2.6, 14.4)
Wilhelm et al. (2008) [54]	California/2000–2001	Children with asthma (CHIS data) (*n* = 607)	Yearly average level (10 ppb)	Asthma ED visits /hospitalizations	OR = 1.35 (0.85, 2.14)
Meng et al. (2010) [53]	San Joaquin Valley/2000–2001	Adults and children with asthma (*n* = 1,502)	Yearly average level (10 ppb)	ED visits/hospitalizations	OR = 1.49 (1.05, 2.11)
Gleason et al. (2014) [62]	New Jersey/2004–2007	Children with asthma (*n* = 21,854)	Daily maximum 8-hr level	Daily asthma ED visits	Same day: OR = 1.05 (1.04, 1.06), Lag 1: OR = 1.04 (1.02, 1.05), Lag 2: 1.00 (0.98, 1.01), 3-day average: OR = 1.04 (1.03, 1.06), 5-day average: OR = 1.01 (0.99, 1.02)
Alhanti et al. (2016) [64]	Atlanta, Dallas, St. Louis/1993–2009	General populations (*n* = 611,970 visits)	3-day moving average level (28 ppb)	Daily asthma ED visits	RR = 1.07 (1.04, 1.10)
Entwistle et al. (2019) [66]	Fresno/2005–2015	ED visits data (*n* = 4,463)	Daily maximum 8-hr level (lag 0–14, 5 ppb)	Asthma ED visits	OR = 1.336 (1.262, 1.414)
Strosnider et al. (2019) [65]	17 U.S. states/2000–2014	General populations (*n* = 39,975,411 visits)	Daily maximum 8-hr level (20 ppb)	Asthma ED visits	RR = 1.055 (1.048, 1.063)
Fishe et al. (2022) [60]	HER data from OneFlorida database /2012–2017	Asthma exacerbation patients (*n* = 351,365)	Daily average level (Q1: ≤31.1ppb, Q2: 31.1-36.4ppb, Q3: 36.4-42.0ppb, Q4: >42.0ppb)	Asthma ED visits (exacerbation)	Q2: OR = 1.01 (1.00, 1.03), Q3: OR = 1.06 (1.05, 1.07), Q4: OR = 1.07 (1.05, 1.08)
Stowell et al. (2024) [61]	U.S./2004–2015	Children’s hospital records (*n* = 405,320)	Daily maximum 8-hr level (10 ppb)	Asthma ED visits	All exposures: RR = 1.013 (1.008, 1.019), Restricting exposures < 70ppb: RR = 1.008 (1.002, 1.014)
Yazdi et al. (2026) [68]	Suffolk County/2014–2023	Stony Brook University Hospital ED visits and inpatient admissions (Not specified)	Not specified	Asthma encounters	Total encounters: RR ≈ 0.998, emergency visits: RR ≈ 0.997, inpatient admissions: RR ≈ 1.001 (approximated from figure; exact 95% CIs not reported)

This table summarizes the subset of studies discussed in the main text. The remaining studies reviewed are available in Supplementary Table S2.; Three papers (Wilhelm et al., [54]; Meng et al., [55], Fishe et al., [60]) report both asthma symptoms and emergency department visit outcomes and therefore appear in both subsections. One paper (Yazdi et al., [68]) reports both asthma-related emergency department visits and hospitalizations and other respiratory-related health outcomes and therefore appears in both subsections

### Asthma-Related Symptoms

Epidemiological studies have linked O_3_exposure to increased asthma risk across diverse populations (Table 2). In a California cohort study of approximately 3,000 non-smoking adults, researchers reported a statistically significant association between 20-year cumulative O_3_ exposure and asthma in men (OR = 2.09, 95% CI: 1.03–4.16) but no relationship in women (OR = 0.86, 95% CI: 0.58–1.26) [52]. The researchers also found that higher O_3_ exceedance frequencies above 60-ppb were statistically significantly associated with increases in asthma risk among men (OR = 1.90, 95% CI: 0.99–3.64) but not in women. In a cross-sectional study using the 2001 California Health Interview Survey (CHIS) with 1,487 adult respondents residing in San Joaquin Valley [53], researchers used the respondents’ self-reported daily and weekly asthma symptoms information and O_3_ concentration measurements from CARB or local air-quality management district monitoring station. It was found that the risk of daily/weekly asthma-related symptoms increased by 23% per 10-ppb increase in annual average O_3_ exposure in the previous year before interview date. In another study linking the 2001 CHIS data and O_3_ measurements from CARB monitoring stations and the California Department of Transportation, the researchers reported that for each 10-ppb increase in annual average O_3_, the odds ratio (OR) for daily weekly asthma-related symptoms was 2.09 (95% CI: 1.28–3.41), and the ORs were increased to 2.29 (95% CI: 1.01–5.23) and 3.51 (95% CI:1.45–8.46) after co-adjusting for PM_10_ and PM_2.5_, respectively [54]. In another study linking 2001 CHIS data in Los Angeles (LA) and San Diego counties with the annual average O_3_ concentration measured at the nearest station, researchers observed that for each 10-ppb increase in the annual average O_3_ exposure prior to the interview date, the risk of poorly controlled asthma increased, particularly among older adults (OR = 1.70, 95% CI: 0.91–3.18) and men (OR = 1.76, 95% CI: 1.05–2.94) [55].

The impact of O_3_ on asthma was also pronounced in children, who tend to spend longer time exercising outdoors (Table 2). In a study with 34,073 U.S.-wide children (ages 3–17), researchers found that for each 5-ppb increase in annual O_3_ exposure, the OR for current asthma was 1.08 (95% CI: 1.02–1.14) [56]. In another study consisting of 224 children with a history of bronchiolitis in infancy selected from the Environmental Influences on Child Health Outcomes (ECHO) PATHWAYS Consortium, the researchers estimated O_3_ exposure generated from a spatiotemporal model based on the geocoded residential address collected at age four. A 2-ppb increase in 2-year average O_3_ exposure had a relative risk (RR) for current wheeze and asthma of 1.3 (95% CI: 1.0–1.7) and 1.4 (95% CI: 1.1–1.8), respectively [57]. A cohort study followed 3,535 children (ages ~ 9–16) across in Southern California over five years and found that the risk of developing asthma in children playing three or more sports in communities with high O_3_ concentrations increased by 30%, compared with children playing no sports, but no association was observed in lower-O_3_ regions [9].

Evidence regarding the association between short-term peaks (over hours to several days) in ambient O_3_ concentrations and asthma-related symptoms and reliance on rescue medications were also reported (Table 2). In a study using a dataset passively collected by digital sensors monitoring inhaled medication use, the researchers analyzed 456,779 short-acting beta-agonist (SABA) puffs inhaled by 3,386 people with asthma or COPD between 2012 and 2019 across California. These researchers used a deletion/substitution/addition (D/S/A) modeling technique to build the surfaces for O_3_ [58]. In the multipollutant model, each 1-ppb increase in daily O_3_ exposure was associated with a RR of 1.005 (95% CI: 1.004–1.007) incremental increase in SABA puffs, and given a 30-ppb increase in O_3_, the incidence rate ratio on daily SABA puff was 1.161 (95% CI: 1.127–1.233).

In a cohort study of 298 asthmatic children in Michigan (1999–2002), using the measurements from two community-level monitoring sites in the area, researchers reported that for each 16-ppb increase in 1-hour peak-O_3_ during the previous 3–5 days, the ORs for wheezing, cough and shortness of breath were 1.12 (95% CI: 1.01–1.23), 1.09 (95% CI: 1.00–1.18) and 1.12 (95% CI: 1.01–1.21), respectively [59]. Similarly, a case-crossover study utilizing 2012–2017 electronic health records from over 533,446 patients in Florida and daily O_3_ data obtained from the US EPA and Centers for Disease Control and Prevention National Environmental Public Health Tracking Network, reported that the risk of acute exacerbation of asthma was found to be 6–7% higher when O_3_ levels was in the upper quartiles (≥ 36.4-ppb) during lag 0–6 days before the event in April, May and October [51]. The estimated effect of O_3_-related asthma exacerbations was largest in Hispanics, followed by Black and then Whites for the fourth O_3_ exposure quartile compared to the first quartile [60].

### Asthma-Related ED Visits/Hospitalizations

Many other observational studies have also evaluated associations between both short- and long-term O_3_ exposure and indicators of asthma severity―ED visits and hospitalizations (Table 2 and Table S2).

Two studies used CHIS data to assess the long-term O_3_ exposure impacts on asthma-related ED visits/hospitalizations (Table 2). One study used data (*n* = 1,502) restricted in San Joaquin Valley in 2001 and O_3_ concentration measurements from the CARB and local air-quality management district monitoring stations, and found that the OR for daily/weekly asthma-related ED visits/hospitalizations was 1.49 (95% CI: 1.05–2.11) for each 10-ppb increase in annual average O_3_ exposure in the previous year before interview date [53]. When stratified by age, there was a 63% increase in ED visits/hospitalizations per 10-ppb increase in O_3_ among children. Expanding on this, another study used O_3_ measurements from CARB monitoring stations and the California Department of Transportation. For each 10-ppb increase in annual average O_3,_ the OR for asthma-related ED visits/hospitalizations was 1.35 (95% CI: 0.85–2.14). The ORs increased to 2.89 (95% CI: 1.32–6.34) and 2.48 (95% CI:1.14–5.38) after adjusting for both PM_10_ and PM_2.5,_ respectively [54]. In a case-crossover study leveraging a dataset of over 405,320 ED visits from 42 U.S. children’s hospitals during 2004–2015, it was observed that each 10-ppb increase in warm-season (May-September) 8-hour maximum daily O_3_ was associated with asthma-related ED visits (RR = 1.013, 95% CI: 1.008–1.019). O_3_ exposure restricted to < 70-ppb was also associated with increases in the risk of ED visits for asthma (RR = 1.008, 95% CI: 1.002–1.014) [61].

An association between short-term increases in ambient O_3_ concentrations and elevated asthma-related ED visits/hospitalizations was also evident, particularly among children, older groups and other vulnerable populations (Table 2). In a case-crossover study linking the daily ED visits among children aged 3–17 from the New Jersy Department of Health and O_3_ concentrations generated from the Community Multi-Scale Air Quality (CMAQ) Model, it was observed that the associations with O_3_ were strongest on the same day of the ED visits (RR = 1.05, 95% CI 1.04–1.06) per 15.5-ppb increase in the O_3_ exposure [62]. In a Washington D.C. study using asthma-related pediatric ED visits (2001–2004) and daily O_3_ concentrations from local monitoring stations, it was reported that each 10-ppb increase in the same day O_3_ concentration being associated with a 4.5% and 8.3% increase in asthma-related ED admissions among the 1–17 years and 5–12 age groups respectively [63]. In another study conducted in three U.S. metropolitan areas (Atlanta, Dallas and St. Louis), it was reported that per 28-ppb increase in 3-day moving average of O_3_ exposure, the RR for asthma-related ED visits was 1.07 (95% CI: 1.04–1.10) in the 5–18 years old, 1.03 (95% CI: 1.01–1.05) in the 0–4 year olds, 1.03 (95% CI: 1.00–1.05) in 19–39 years old, 1.01 (95% CI: 0.98–1.04) in 40–64 years old, and 1.03 (95% CI: 0.97–1.09) in those 65 years and older [64] (Table 2). Similar findings were also observed in a study of over 39 million ED visit records across 17 U.S. states; it was reported that the RR for asthma-related ED visits was 1.06 (95% CI: 1.05–1.06) per 20-ppb increase in O_3_ on lag days 0–6 [65]. When stratified by age, the RRs for those 0–18, 19–64 and 65 + were 1.04 (95% CI: 1.03–1.06), 1.07 (95% CI: 1.06–1.08), 1.02 (95% CI: 1.00–1.05), respectively. In a case-crossover study in Fresno, California, it was observed that a 5-ppb increase in the concentration of O_3_ at the lag 0–14 days was associated with a 34% increased risk of having an asthma ED visit. When stratified by age, the ORs for having asthma-related ED visits were 1.42 (95% CI: 1.26–1.60), 1.25 (95% CI: 1.16–1.39), 1.34 (95% CI: 1.19–1.49) and 1.36 (95% CI: 1.06–1.71) for those who were aged 2–5, 6–18, 19–40, 41–64 and 65 + years for each 5-ppb increase in the average O_3_ concentration of lag 0–14 days, respectively [66]. When stratified by race/ethnicity, the risk increased by 40%, 38% and 27% among Hispanics, Blacks and Whites at the cumulative lag 0–14, respectively (Table 2). Although the evidence from most studies was suggestive of an association between O_3_ exposure and asthma-related ED visits/hospitalizations, some others reported non-significant results. In a study examining hospital discharge and exposure to O_3_ for 1991–1994 central LA, neither daily average on admission day nor 8-day average of O_3_ concentration was found to be significantly related to asthma-related hospital admissions [67]. Similarly, in a recent study using ED visit and inpatient admission data from Stony Brook University Hospital in New York State between 2014 and 2023, no positive associations were observed between daily O_3_ exposure and asthma-related ED visits or inpatient admissions [68].

## Other Respiratory-Related Mortality and Morbidity

Although literature has largely emphasized the respiratory effects of O_3_ exposure, including asthma exacerbation, an expanding body of evidence implicates both short- and long-term exposure in respiratory mortality and a broader range of adverse respiratory outcomes (Table 3 and Table S3).

**Table 3:** Selected studies of ozone exposure and other respiratory-related mortality and health outcomes

Author (year)	Study Region/year	Study Population	Exposure Metrix (per unit increase)	Health Outcome	Effect Estimate
Other respiratory-related mortality					
Abbey et al. (1999) [70]	California/1977–1992	Adults (*n* = 6,338)	Monthly 8-hr average (> 100 ppb)	Lung cancer mortality	Male: RR = 4.19 (1.81, 9.69), Female: RR = 1.39 (0.52, 3.67)
Bell et al. (2004) [5]	95 U.S. urban communities/1987–2000	Death records from the communities (Not reported)	Daily 8-hour average (10 ppb)	Cardio-pulmonary related mortality	Daily counts: +0.52% (0.27%, 0.77%)
Ritz et al. (2006) [77]	Southern California/1989–1999	Infants (*n* = 19,664 deaths)	2-week, 1-month, 2-month, 6-month average levels before death (10 ppb)	Post-neonatal respiratory deaths (28 day to 1 year)	OR = 1.03 (0.93, 1.14)
Jerrett et al. (2009) [8]	96 U.S. metropolitans/1977–2000	Adults (*n* = 448,850)	Quarterly average (10 ppb)	Respiratory mortality	RR = 1.04 (1.01, 1.05)
Ren et al. (2010) [75]	Suffolk, Middlesex, Norfolk/1995–2002	Adults (*n* = 162,146)	4-day moving average of 8-hr max level (10 ppb)	Respiratory mortality	% change in mortality: +1.96% (–1.83%, 5.90%)
Jerrett et al. (2013) [74]	Los Angeles/1982–2000	Adults (*n* = 79,711)	Monthly average (24 ppb)	Respiratory and lung cancer related mortality	Respiratory: RR = 1.017 (0.902, 1.147), Lung cancer: RR = 1.015 (0.747, 0.992)
Lim et al. (2019) [72]	6 U.S. states and 2 metropolitans/1995–2011	Adults (*n* = 548,780)	Annual average level during warm season (10 ppb)	Respiratory, COPD, pneumonia, lung cancer related mortality	Respiratory: HR = 1.04 (1.00, 1.08), COPD: HR = 1.06 (1.01, 1.12), Pneumonia: HR = 1.06 (0.99, 1.14), Lung Cancer: HR = 0.98 (0.95, 1.00)
Kazemiparkouhi et al. (2020) [73]	U.S./2000–2008	Medicare beneficiaries (*n* = 22.2 million)	1-hr max during warm season (10 ppb)	Respiratory, COPD, lung cancer related mortality	Respiratory: RR = 1.03 (1.03, 1.04), COPD: RR = 1.07 (1.06, 1.07), Lung Cancer: RR = 1.02 (1.01, 1.02)
Liu et al. (2022) [76]	7 U.S. cities/2000–2015	Non-accidental mortality and death certificate data (*n* = 323,309 deaths)	Daily 8-hr max level with lag 0–2 (10 ppb)	Respiratory mortality	Mortality: 0.41% (–0.33%, 1.15%)
Mowla et al. (2026) [78]	U.S.A/2005–2017	livebirth (*n* = 35,665,533)	Monthly average (10ppb)	Infant respiratory-related mortality	Northwest: IRR = 1.06 (95% CI: 1.04–1.09); Counties with high SVI: IRR = 1.08 (95% CI:1.03–1.13); Warm Temperature: IRR = 1.03 (95% CI: 0.97–1.05); Male infant: IRR = 1.15 (95% CI: 0.95–1.40); Parent aged 26–35 at delivery: 1.25 (95% CI: 0.92–1.75); Parent had greater than high school education: IRR = 1.10 (95% CI: 0.95–1.20)
Other respiratory-related health outcomes					
Ostro et al. (1989) [88]	U.S./1976–1981	Adults (*n* = 7,233 - 8,243)	2-week daily average level (0.51 ppb)	Respiratory-related restricted activity days (RRAD)	𝛽 = 0.18–0.33 (*p* > 0.05)
Chen et al. (2000) [89]	North Nevada/1996–1998	Elementary students (*n* = 27,793)	14-day average level (50 ppb)	All-cause school absence	+ 13.0% (3.4%, 22.6%)
Gilliland et al. (2001) [90]	12 communities near Los Angeles/1996	4th graders students (*n* = 2,068)	8-hour average level (20 ppb)	All school absence, Illness-related absence, Respiratory-related absence	All school absence: +16.3% (-2.6%, 38.9%), Illness-related absence: +62.9% (18.4%, 124.1%), Respiratory-related absence: +82.9% (3.9%, 222.0%)
Rondeau et al. (2005) [91]	12 communities near Los Angeles/1996	4th graders students (*n* = 1,932)	2-year community average level (20 ppb)	Overall school absence, Respiratory-related absence	Overall absence: +30.9% (14.2%, 50.3%), Respiratory-related absence + 27.1% (4.5%, 54.6%)
O’Connor et al. (2008) [93]	7 U.S. urban cities/1998–2001	Children with persistent asthma (*n* = 861)	19-day average level (26.7 ppb)	Overall school absence	OR = 1.31 (0.83, 2.06)
Darrow et al. (2014) [84]	Atlanta/1993–2010	ED visits data (*n* = 503,004)	3-day moving average level (27.8 ppb)	Upper Respiratory Infection (URI) ED visits, Pneumonia ED visits	URI: RR = 1.041 (1.019, 1.064); Pneumonia: RR = 1.083 (1.038, 1.131)
Magzamen et al. (2017) [83]	Washington/1990–2006	Hospital admission data (*n* = 104, 468)	Daily max 8-hr level (lags 0–3 days) (10 ppb)	Respiratory hospital admissions	Clark County: RR = 1.04 (1.02, 1.07); Spokane County: RR = 1.03 (1.01, 1.05) King County: RR = 1.02 (1.01, 1.03)
Rhee et al. (2019) [81]	U.S./2000–2012	Medicare data (*n* = 1,164,784)	Annual average level (1 ppb)	Annual Acute Respiratory Distress Syndrome [79] hospital admission rates	Annual hospital admission rate for ARDS: +0.24% (0.18%, 0.31%)
Mendoza et al. (2020) [92]	Salt Lake City/2015–2018	K-12 students (26 elementary schools + 7 middle schools + 3 high schools)	Daily average level (1 ppb)	Overall school absence	RR ≈ 1.01 (*p* < 0.05)
Paulin et al. (2020) [82]	7 cities in U.S./2010–2018	SPIROMICS AIR participants (*n* = 1,874)	10-year average level (5ppb)	Severe Chronic Obstructive Pulmonary Disease (COPD) outcomes (e.g. emphysema, air trapping)	emphysema: 𝛽=0.94 (0.25, 1.64), higher odds of any exacerbation: 𝛽 = 1.37 (1.12, 1.66), Severe exacerbation: 𝛽=0.94 (1.07, 1.76)
Diver et al. (2026) [85]	21 U.S. states/ 1992–2017	Adults (*n* = 122,442)	Time-varying annual average (9.7ppb)	Lung cancer incidence	Lung cancer: HR = 1.07 (95% CI: 1.01–1.12); non-small cell lung cancer: HR = 1.05 (95% CI: 0.99–1.12), Squamous cell: HR = 1.01 (95% CI: 0.89–1.15), Adenocarcinoma: HR = 1.05 (95% CI: 0.96–1.14), Large cell carcinoma: HR = 1.13 (95% CI: 0.97–1.33); Small cell carcinoma: HR = 0.99 (95% CI: 0.84–1.18); Other carcinoma: HR = 1.03 (95% CI: 0.89–1.18)
Morphew et al. (2026) [94]	Pittsburgh, Pennsylvania/2015/16-2017/18	School attendance data from the Clairton School District of Pittsburgh (not specified)	Daily average of 8 h-maximum concentration (≥ 55 ppb vs. <55ppb)	School absence (non-asthmatic and asthmatic)	Asthmatic absence: RR = 0.94 (95% CI: 0.79–1.11); non-asthmatic absence: RR = 1.00 (95% CI: 0.88, 1.27)
Yazdi et al. (2026) [68]	Suffolk County/2014–2023	Stony Brook University Hospital ED visits and inpatient admissions (Not specified)	Not specified	Respiratory disease encounters	Total encounters: RR ≈ 1.00; emergency visits: RR ≈ 1.00; inpatient admissions: RR ≈ 1.00

This table summarizes the subset of studies discussed in the main text. The remaining studies reviewed are available in Supplementary Table S3. One paper (Yazdi et al., [68]) reports both asthma-related emergency department visits and hospitalizations and other respiratory-related health outcomes and therefore appears in both subsections

### Respiratory-Related Mortality

The evidence for adverse health effects associated with long-term exposure to O_3_ is mixed (Table 3 and Table S3), while the evidence from systematic review and meta-analyses is suggestive of associations with respiratory mortality [69]. In an earlier study of 6,338 nonsmoking Californian from Adventist Health Study, researchers linked O_3_ concentrations from 348 monitoring stations to 1977–1992 mortality of the cohort and reported a strong association with lung cancer mortality among men (RR = 4.19, 95% CI: 1.81–9.69) when O_3_ exposures exceeded 100-ppb [70] (Table 3). In a respiratory cancer study among Whites in LA and Honolulu, it was observed that the risk of cancer death was 1.06 (95% CI: 1.04–1.07) for each 10-ppb increase in annual average O_3_ exposure [71]. In a study using the American Cancer Society Cancer Prevention Study II cohort (*n* = 448,850 subjects with 118,777 death) and correlating with air-pollution data from 96 metropolitan statistical areas in the U.S., it was reported that the RR of respiratory-specific death was 1.04 (95% CI: 1.01–1.07) for each 10-ppb increase in quarterly average (April–September) O_3_ concentration [8]. In a cohort of over 500,000 U.S. adults aged 50–71, the researchers assigned annual O_3_ concentrations to each participant’s residence census tract using EPA Fused Air Quality Surface Using Downscaling data and reported that the hazard ratios for respiratory disease and COPD mortality were 1.04 (95% CI: 1.00–1.09) and 1.06 (95% CI: 1.01–1.12) per 10-ppb increase in 8-year average O_3_ exposure [72]. In another study examining the association between long-term warm-season averaged 1-hour maximum O_3_ exposure on cause-specific mortality for 22.2 million U.S. Medicare beneficiaries between 2000 and 2008, the researchers found strongest and most consistent increased risks for all respiratory mortality (RR = 1.03, 95% CI: 1.03–1.03), COPD-related mortality (RR = 1.07, 95% CI: 1.07–1.08) and lung cancer-related mortality (RR = 1.02, 95% CI: 1.01–1.02) [73]. While in a cohort of 79,711 California adults (1982–2000) in LA, the researchers calculated the O_3_ concentrations from inverse distance square weighting (IDW) models and reported that for each 24-ppb increase in 14-year average O_3_ exposure, the RR for all-caused respiratory mortality was 1.02 (95% CI: 0.90–1.15) [74] (Table 3).

Short-term O_3_ exposure has also been associated with elevated respiratory mortality, although significant results are not consistently observed across studies (Table 3). In a national study using daily cause specific mortality counts for 1987–2000 from the National Center for Health Statistics on 95 large urbanized areas and O_3_ data from US EPA AQS, the researchers reported that a 10-ppb increase in one-week average O₃ exposure was associated with a 0.52% increase in daily cardio-pulmonary mortality [5]. In a case-crossover study consisting of 162,146 deceased subjects aged 35 + from three counties in the eastern Massachusetts during 1995–2002, it was reported that each 10-ppb increase in the average of 8-hour-maximum O_3_ in the previous four days was associated with 1.96% (95% CI: -1.83–5.90%) increase in deaths from respiratory disorders [75]. Similarly, in a case-crossover study estimating the associations between short-term O_3_ exposure and all-cause and cause-specific mortality in seven U.S. states from 2000 to 2015, the researchers linked daily O_3_ exposure levels to over 3 million non-accidental death records based on census tracts or residential addresses and found a 0.41% (95% CI: -0.33–1.15%) increase in respiratory mortality for each 10-ppb increase in O_3_ exposure at lag 0–2 day [76].

Few studies investigated the association between O_3_ exposure and respiratory death among infants (Table 3). In a case-control study evaluating the influence of outdoor air pollution on infant all respiratory-caused death in the South Coast Air Basin of California, the researchers identified all infant deaths among infants born alive between 1989 and 2000 and calculated average birth address-based O_3_ exposure levels during the 2-week, 1-month, 2-month, and 6-month periods before a case subject’s death, but no association between O_3_ exposure and infant respiratory death was found [77]. A recent study examined monthly O_3_ exposure in relation to all-cause and cause-specific post-neonatal infant mortality using National Center for Health Statistics (NCHS) for all live births (*n* = 35,665,533) occurring in the contiguous U.S. between 2005 and 2017. Positive associations between average monthly O_3_ exposure and infant respiratory-related mortality were observed among infants living in Northwest (IRR = 1.06, 95% CI: 1.04–1.09) or counties with high social vulnerable index (SVI) (IRR = 1.08, 95% CI:1.03–1.13) [78].

### Other Respiratory-Related Morbidity

O_3_ exposure has been implicated in a range of respiratory health endpoints, including bronchitis, pneumonia and COPD, and acute respiratory distress syndrome [79]. Both long-term and short-term O_3_ exposures contribute to these outcomes, with effect sizes varying across different populations (Table 3 and Table S3).

In a cohort of Medicare recipients (*n* = 11,084,660) in seven southeastern states linking spatial-temporal model generated annual average O_3_ exposure from 2000 to 2012, the researchers found that for each 1-ppb increase in annual average O_3_ exposure, the hazard ratios for COPD, pneumonia and lung cancer-related hospitalizations were1.02 (95% CI: 1.02–1.03), 1.03 (95% CI: 1.03–1.03) and 1.004 (95% CI: 1.002–1.005), respectively [80] (Table 3). Similarly, using 2000–2012 Medicare data, it was observed that for each 1-ppb increase in the annual average O_3_ exposure, there was an increase in annual hospital admission rate for ARDS of 0.24% (95% CI: 0.18–0.31) in single pollutant model and 0.15% (95% CI: 0.08–0.22) when co-adjusting for PM_2.5_ [81]. In a cross-sectional study of 1,874 current and former smokers with or without chronic obstructive pulmonary disease the SPIROMICS AIR study across seven U.S. cities [82], a 5-ppb increase in 10-year average O_3_ concentration was associated with a higher risk of respiratory exacerbation (OR = 1.37, 95% CI: 1.12–1.66) and severe exacerbation (OR = 1.37, 95% CI:1.07–1.76). In a 16-year longitudinal study in Washington State, the researchers linked hospital admission and ambient monitoring data and found that for each 10-ppb increase in daily 8-hour maximum O_3_ at a 3-day lag, the RR for respiratory-related hospital admissions was increased by 2–4% [83]. Using the ED visits data among children (aged 0–4 years) within the Atlanta metropolitan area and O_3_ concentrations from ambient monitoring networks, the researchers found elevated risk of ED visits related to upper respiratory infection (URI; RR = 1.04, 95% CI: 1.02–1.06) and pneumonia (RR = 1.08, 95% CI: 1.04–1.13) for each 28-ppb increase in 3-day average O_3_ [84] (Table 3). Recently, in the American Cancer Society’s Cancer Prevention Study-II Nutrition Cohort, which included 122,442 participants, each 9.7-ppb increase in annual average O_3_ exposure was associated with a 7% higher risk of incident lung cancer (HR = 1.07; 95% CI: 1.01–1.12). Positive associations were also observed for nearly all lung cancer subtypes except small cell carcinoma, although the 95% CIs included the null [85]. These studies collectively highlight the role of transient O_3_ spikes in triggering acute respiratory events, reinforcing the need for real-time air quality monitoring and risk communication, especially for vulnerable populations. Although rare, some studies also indicated the acute impacts on restricted activity days and school absence due to some symptom exacerbations that are not perceived as severe enough to search for medical assistance [86]. Using the Health Interview Survey (HIS) 1976–1981, the researchers investigated the association between respiratory-related restricted activity days (RRAD) and minor restricted activity days (MRAD) [87] with 2-week average O_3_ exposure obtained from EPA’s Storage and Retrieval of Aerometric Data (SAROAD) monitoring network. O_3_ was observed to be associated with MRAD but not RRAD [88] (Table 3).

Only six studies have examined the association between O_3_ exposure and school absenteeism in U.S (Table 3). In the northern Nevada study (1996–1998), the researchers included 57 elementary schools with a total of 27,793 student enrollments, and accessed continuously hourly O_3_ concentrations from six monitoring sites in the Washoe County [89]. It was observed that for each 50-ppb increase in 1-hour maximum O_3_ concentration in the preceding 14 days, the absence rate would increase 13.01% (95% CI: 3.41–22.61%), indicating the absenteeism numbers for all elementary schools in the area will increase by 160 (95% CI: 42–278) each day due to the increase in O_3_. Another two studies nested in the Children’s Health Study in Southern California examined type-specific absence incidence data collected by an active surveillance system for the school children aged 9–10 years during January through June 1996 [90, 91]. Hourly-average O_3_ was measured at central-site monitors in each of the 12 communities. It was observed that an increase of 20-ppb of O_3_ concentration with 30-day lag period was associated with an increase of 16.3% (95% CI: -2.6–38.9%) for all school absence, 62.9% (95% CI: 18.4–124.1%) for illness-related absence rates, 82.9% (95% CI: 3.9–222%) for respiratory illnesses. An increase of 20-ppb of O_3_ concentration with 15-day lag period was related to an increase of 45.1% (95% CI: 21.3–73.7%) for upper respiratory illnesses, and 173.9% (95% CI: 91.3–292.3%) for lower respiratory illnesses with wet cough [90]. For each 20-ppb increase in annual average O_3_ exposure, there was an increase of 30.9% (95% CI: 14.2–50.3%) for all school absence, 25.9% (95% CI: 1.5–56.1%) for illness-related absences and 27.1% (95% CI: 4.5–54.6%) for respiratory illness-related school absence, respectively [91]. In a Utah study using the absence data from 26 elementary schools, 7 middle schools and 3 high schools located in the Salt Lake City School District [92], the researchers modelled O_3_ concentrations using an IDW method, and reported a positive relationship with prior day O_3_ exposure and school absence across the full academic year, Winter and Spring quarters. While in another study, researchers recruited 861 children with persistent asthma in seven U.S. urban communities and asked the caregivers to recall the number of days in the past 2 weeks that the child missed school because of asthma [93]. For each 26.7-ppb increase in the 19-day average O_3_ concentration, the risk ratios for school absence in single-pollutant and co-pollutant models were 1.31 (95% CI: 0.83–2.06) and 1.35 (95% CI: 0.82–2.20), respectively. Similarly, a recent study using the daily school attendance data (2015/16―2017/18) from the Clairton School District of Pittsburgh, Pennsylvania and outdoor air pollution data from US EPA AQS reported no association between O_3_ exposure and school absenteeism. Compared with days with a maximum 8-hour O_3_ concentration < 55-ppb, days with O_3_ concentrations ≥ 55-ppb were associated with rate ratios of 0.94 (95% CI: 0.79–1.11) for absenteeism among students with asthma and 1.00 (95% CI: 0.88, 1.27) for non-asthmatic absence [94].

## Discussion

**Fig. 2:**
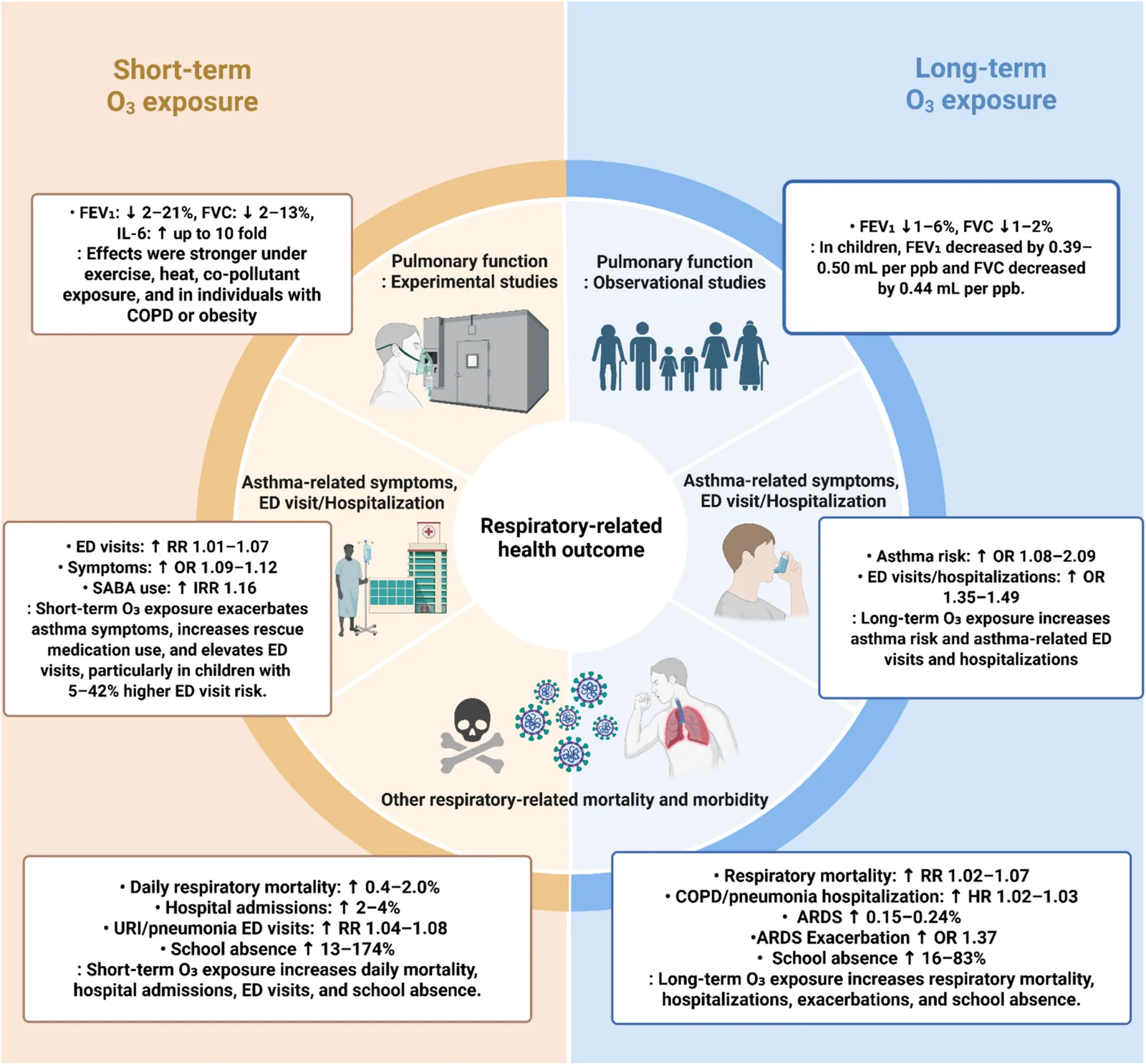
Short-term and long-term O_3_ exposure effects on respiratory health outcomes

We reviewed the literatures regarding the respiratory health outcomes including pulmonary function, asthma-related symptoms, ED visits/hospitalizations, as well as other respiratory-related morbidity and mortality due to O_3_ exposure, summarizing the findings that both short-term and long-term O_3_ exposures could contribute to pronounced effects for a broad range of respiratory health outcomes, especially among vulnerable populations such as children, older adults, and individuals with pre-existing health conditions (Fig. 2).

We started the literature review from pulmonary function because it is an objective and important measure of respiratory health and a predictor of cardiorespiratory morbidity and mortality [95], highlighting lung function as a mechanistically informative and policy-relevant outcome. Human experiment studies are usually considered in the context of the US EPA’s revised standards for O_3_ [96], consistently report small but measurable reductions in lung function, often accompanied by markers of airway inflammation, thus providing important insights into the short-term respiratory effects of O_3_, particularly under conditions of elevated ventilation such as exercise. However, several limitations are inherent in these experimental designs. First, previous experimental studies primarily evaluate pulmonary function after acute exposure, often in controlled environments that do not fully replicate real-world conditions. Second, these studies are typically conducted on healthy adults, thus limiting their generalizability to vulnerable populations such as children, the older population, and individuals with pre-existing conditions. Third, the intensity and duration of exercise used in these studies often exceed those encountered in daily life, further restricting their applicability [96]. Fourth, experimental studies benefit from repeated measures within the same individuals, strengthening within-person comparisons and offering valuable insights into the role of individual characteristics; however, the studies depend on participants’ collaboration and generally involve relatively small sample sizes and selected study populations, which may limit the generalizability of their findings to broader populations. Furthermore, multiple comparisons (i.e. paired test, two-way ANOVA) in these studies might introduce the risk of spurious findings. Therefore, although valuable for understanding specific biological responses, findings from these experimental studies may not fully represent responses across more diverse populations.

Alternatively, observational studies are more generalizable and can assess O_3_-related declines in lung function even among children. The camp study design enables researchers to collect repeated lung-function measurements along with standardized outdoor physical activities and examine within-subject variations in pulmonary responses to O_3_ exposures without ethical constraints. These studies are particularly important given children’s greater vulnerability to air pollution owing to higher ventilation rates per body weight and greater time spent outdoors, providing robust evidence that short-term O_3_ exposure―even at relatively low ambient concentrations―is associated with reductions in lung function among children. The findings are consistent across diverse settings, exposure metrics, and analytic approaches, and contribute meaningfully to the broader body of epidemiologic evidence on O_3_-related respiratory health effects in sensitive populations.

Observational studies including longitudinal, case-control, case-crossover and cross-sectional studies also present significant challenges. Most studies relied on administrative health records which constrained exposure assessment to ZIP code–level estimates rather than geocoded address-based estimates, thus limiting spatial resolution for O_3_ exposure and raising the possibility of O_3_ exposure misclassification. Also, community-level exposure assessments from fixed-site monitors were often used in previous studies, which might not capture the variability in individual exposure, especially for children or low-income populations with longer time spent performing outdoor activities. For example, among the six studies included in this review that investigated O_3_-related school absenteeism, air pollution exposures predominantly used community level measurements made by centrally located monitors, rather than using personal exposure assessments. Additionally, without incorporating occupational environment and residential history, potential misclassification of long-term O_3_ exposure cannot be ruled out. Furthermore, various exposure assessment methods might also contribute to inconclusive results. Most studies use the O_3_ concentration measured at governmental monitoring sites, restricting the studies limited areas and time periods [59, 90]. A few studies assessed the O_3_ exposure levels using spatial and/or temporal models such as IDW, however, there is still a lack of well-developed exposure modeling approach for O_3_ [97]. Further refinements in exposure modeling include the development of advanced exposure assessment models such as chemical transport models or land use regression models combined with monitoring data, land use, traffic and meteorological data are warranted.

Studies that isolate O_3_ exposure while limiting consideration of other contributing factors may over/underestimate health risks in such exposure contexts. Ambient O_3_ rarely acts alone. Rather, individuals are typically co-exposed to multiple pollutants, particularly in regions like California, where photochemical smog contains co-occurring pollutants such as PAN. Another pressing concern is the growing need to better characterize O_3_ exposure from wildfires. Given the increasing frequency and severity of wildfires, it is critical to examine the spatial and temporal impacts of these events on O_3_ levels to distinguish their effects from conventional emissions. Additionally, factors such as neighborhood air quality, access to green spaces, and dietary habits may also modify individual susceptibility to O_3_-related health effects. For example, dietary factors may increase susceptibility to pollutant-related health effects independently of socioeconomic status. A growing body of evidence suggests that diets rich in fruits and vegetables [98], antioxidant vitamin supplementation, and dietary fatty acids may help mitigate adverse respiratory effects associated with outdoor air pollution, supporting an important role for oxidative stress in the underlying biological pathways [99–101]. Other vulnerability factors such as obesity and low birth weight also need to be accounted for in the analysis [102]. Further, the adverse effects of O_3_ exposure might vary over the life-course (including prenatal exposures) such that future research should be directed toward differential effects at different stages of development.

Our review also demonstrated there were limited studies investigating various effects of O_3_ exposure across vulnerable populations. For example, effects of ethnicity might be confounded with those effects associated with low socioeconomic status. Children from socioeconomically disadvantaged families may experience higher ambient air pollution exposure and greater vulnerability to its health effects due to environmental inequities and co-occurring social and health-related risk factors [103, 104]. A nationwide U.S. study of public schools also found that schools with higher proportions of students from lower-income households, as indicated by eligibility for free or reduced-price lunch, were exposed to higher ambient air pollutant concentrations, particularly NO₂ [105, 106]. Also, few studies have considered factors such as maternal or early-life exposures, which are often shaped by parental socioeconomic status and may influence long-term respiratory outcomes.

For certain respiratory health outcomes such as asthma, in view of how important O_3_ pollution is as a trigger of exacerbations, one of the key questions is whether short-term peak exposures (e.g., 1-hour peak) versus time-weighted averages over longer time periods (e.g., 1–2 days) are more strongly associated with increased risk of adverse asthma outcomes. Some evidence points to peak exposures being more detrimental than long-term exposure, yet further investigation is needed. Furthermore, the lag time between O_3_ exposure and asthma impacts remains uncertain. Many studies report stronger adverse health outcomes occurring the day after high O_3_ exposure rather than on the same day [107]. Also, several studies used case-crossover design to control for many potential individual level confounders (e.g., socioeconomic status, body mass index, tobacco use), given that each case serves as its own control [60]. However, many studies fail to account for other important variables such as maternal age, gestational duration, or family history of asthma or allergy, all of which may confound or modify the relationship between O_3_ exposure and asthma-related health outcomes.

Several strengths need to be noted. First, unlike many prior reviews that primarily focused on pulmonary function or ED visits/hospitalizations separately, we provided an integrated synthesis of research findings spanning from lung function to respiratory-related mortality, enhancing our understanding of how O_3_ affects respiratory health across the continuum from early dysfunction to overt disease. Second, this review uniquely included school absenteeism as a meaningful and policy-relevant indicator of O_3_-related health impacts. Although not as specific as other health indicators (e.g. asthma-related ED visits), school absenteeism captures both minor and intermediate health consequences that are severe enough to prevent them from attending school but not necessarily leading to dramatic consequences such as ED visits/hospitalizations. Therefore, it is a good indicator for school-aged children to reflect their overall health condition and providing a more comprehensive assessment of the adverse impacts of O_3_ exposure [92, 108]. The inclusion of school absenteeism as an outcome further emphasizes the broader societal and economic consequences of O_3_ exposure including parental work loss and associated productivity costs [108–110], suggesting that their public health impacts may extend beyond the immediate health of the children.

## Conclusions

By integrating evidence from both controlled human exposure experiments and epidemiologic studies conducted in the U.S., this systematic review brings together complementary evidence on O₃-related respiratory health effects across experimental and population-based settings, suggesting relatively consistent associations between O_3_ exposure and impaired pulmonary function, asthma-related health effects, and other respiratory-related mortality and morbidity including school absenteeism. The associations observed between O_3_ exposure and multiple respiratory health outcomes—even at concentrations below current regulatory standards—highlight important implications for U.S. air quality policy. These findings suggest that existing O_3_ standards may not fully protect vulnerable populations and underscore the importance of continued reassessment of regulatory thresholds, particularly in light of climate-driven changes in O_3_ formation and wildfire-related emissions. In addition to regulatory considerations, the evidence supports targeted public health interventions, improved risk communication, and focused protection of high-risk populations to mitigate O_3_-related respiratory health impacts.

Future studies are encouraged to incorporate O_3_ exposures estimated derived from advanced exposure models within multipollutant frameworks to capture the complexity of real-world exposures and individual-level vulnerability, as well as standardizing definitions and improving confounders control which are critical to enhance comparability across the studies. Despite a substantial body of literature, more research efforts are needed and essential for informing equitable air quality standards and guiding targeted public health interventions to effectively reduce the respiratory health burden associated with O_3_ exposure.

## Supplementary Information

Below is the link to the electronic supplementary material.

Supplementary Material 1 (DOCX 3.00 MB)

## Data Availability

No datasets were generated or analysed during the current study.
