# Supplementary material for: The Impact of Ambient Ozone Exposure on Respiratory Health in the U.S. Populations: A Systematic Review
Source: Curr Environ Health Rep. 2026 Oct 17;13(1):47. doi: 10.1007/s40572-026-00568-0 (PMC13676670; doi:10.1007/s40572-026-00568-0)
Supplement: Supplementary file 1 [file 40572_2026_568_MOESM1_ESM.docx]

**Supplementary Materials**

# **Title: The Impact of Ambient Ozone Exposure on Respiratory Health in the U.S. Populations: A Systematic Review**

**Authors:** Yoojin Cho^1^, Yu Yu^1,2^, Xinyu Zhang^2^, Michael Kleeman^3^, Michael Jerrett^4^, Ninez A. Ponce^2,5*^

^1^ Department of Epidemiology, Fielding School of Public Health, University of California Los Angeles, California, USA

^2^ Center for Health Policy Research, University of California Los Angeles, California, USA

^3^ Department of Civil and Environmental Engineering, University of California Davis, California, USA

^4^ Department of Environmental Health Sciences, Fielding School of Public Health, University of California Los Angeles, California, USA

^5^ Department of Health Policy and Management, Fielding School of Public Health, University of California at Los Angeles, California, USA

**Corresponding authors information:** Addresses correspondence to **Ninez A. Ponce**, UCLA Center for Health Policy Research, University of California at Los Angeles, California, USA, 10960 Wilshire Blvd, Suite 1550, Los Angeles, CA 90024; Tel (310) 794 2691; Email: [nponce@ucla.edu](mailto:nponce@ucla.edu).

**Search Strategies for Systematic Review**

Our search strategies employed the following keyword combinations to align with the scope of this review:

<PubMed>

("O_3_" OR "Ozone"[MeSH] OR "Ozone" OR "Trioxygen" OR "Three oxygen molecules*") AND ("Direct" OR "Indirect" OR "Social Risk" OR "Asthma burden" OR "CHIS data" OR "School absence" OR "Work loss" OR "Asthma*" OR "Respiratory*" OR "ED visit" OR "Hospitalization" OR "Hospital Admission") AND ("California*" OR "CA" OR "United States" OR "U.S." OR "U.S.A.")

<Web of Science>

("O_3_" OR "Ozone" OR "Trioxygen" OR "Three oxygen molecules*") AND ("Direct" OR "Indirect" OR "Social Risk" OR "Asthma burden" OR "CHIS data" OR "School absence" OR "Work loss" OR "Asthma*" OR "Respiratory*" OR "ED visit" OR "Hospitalization" OR "Hospital Admission") AND ("California*" OR "CA" OR "United States" OR "U.S." OR "U.S.A.")

**Quality Assessment and Risk of Bias Evaluation Using the JBI Critical Appraisal Tools**

We assessed study quality using the Joanna Briggs Institute (JBI) critical appraisal tools (<https://jbi.global/critical-appraisal-tools>). Because JBI tools are organized by study design (e.g., cohort, case–control, cross-sectional, randomized controlled trial), studies that did not map cleanly to a single category were first discussed among reviewers. In such cases, we reached consensus on the best-fitting design based on the study’s data-collection methods; where specific items were clearly not applicable, we proceeded with minor adaptations to the checklist.

For each section, two reviewers independently appraised all included studies against the relevant JBI items. We then synthesized the ratings to a final decision, taking into account both the inter-reviewer agreement and each reviewer’s proportion of “Yes” responses. Operationally, studies were classified as: Low risk of bias (good quality) when both reviewers’ “% Yes” and the inter-reviewer agreement were ≥70%; Moderate risk of bias (moderate quality) when any of these metrics fell between 60% and <70%; and High risk of bias (poor quality) when any metric was <60% [1]. In the figures (Figure S1-S3), each heatmap cell displays the final decision (High/Moderate/Low), while the cell color encodes the inter-reviewer agreement, so that both individual reviewer rigor and between-reviewer concordance are conveyed simultaneously.

| **Table S1. Observational and Experimental Studies of Ozone Exposure and Pulmonary Function (Not Included in the Main Text)** | | | | | |
| --- | --- | --- | --- | --- | --- |
| **Author (year)** | **Study Region/year** | **Study Population** | **Exposure Metrix** | **Health Outcome** | **Effect Estimates** |
| **Experimental Studies** | | | | | |
| Folinsbee et al. (1980)^1^ | California | Male adults (n=30) | 0.20, 0.35, 0.50 ppm for 2 hr on each of 5 days | IC, ERV, FVC, FEV1, FEV2, FEV3, FEF50, FEF75, FEF25-75, MVV, FRC | ↓ FVC at 0.35(Day 3), 0.5 (Day 2, 3) ppm; ↓ FEV1 at 0.35(Day 2, 3), 0.5(Day 2,3,4) ppm; ↓FEV2 0.35(Daty 3) ppm; ↓FEF75 at 0.35(Day 2), 0.5(Day 2,3)ppm; ↓FEF25-75 at 0.35(Day 2, 3), 0.5(Day 2,3,4)ppm; ↓IC at 0.5 ppm; No association was found for other lung functions |
| Linn et al. (1980)^2^ | Southern California | Adults (n=64) | 0.22ppm O_3_, 200ug/m3 TSP/2hr | FEV1, FVC, PEFR, TLC, symptoms | ↓FEV1, ↓FVC, ↓PEFR, ↓TLC but no difference between healthy ans asthmatic groups; ↑symptoms only in asthmatic group |
| Adams et al. (1981)^3^ | Davis | Male adults (n=8) | 0, 0.20, 0.30, 0.40 ppm/120min | FVC, FEV1, MMFR, RV, TLC | ↓FVC, ↓ FEV1, ↓MMFR, ↓RV, ↓TLC starting between 0.20 and 0.30ppm |
| Dimeo et al. (1981)^4^ | California/2005-2010 | Adults (n=19) | 0.2, 0.4 ppm/2hr | Bronchial reactivity to inhaled histamine | ↑(reactivity), ↓(adaptation) |
| Folinsbee et al. (1981)^5^ | Santa Barbara | Male adults (n=8) | 0.5ppm O3, 0.5ppm NO2/2hr | VC, IC, ERV, FVC, FEV1, FEF50%, FEF25-75% | ↓VC, ↓ IC, ↓FEV1, ↓FEF50%, ↓FEF75%, but no synergism was observed |
| Horvath et al. (1981)^6^ | Santa Barbara | Male adults (n=24) | 0.5ppm/2hr/5 consecutive days | FVC, FEV1, FEF25-75% | ↓ FEV1, ↓ FVC, ↓FEF25-75%, with greatest decrement on 2nd day, adaption production requires 2-5 days, can averagely last for 7-14days |
| Mihevic et al. (1981)^7^ | Santa Barbara | Male adults (n=14) | 0.0, 0.3, 0.5ppm/40min | FVC, FEV1, FEF 25-75% | ↓ FVC, ↓ FEV1, ↓ FEF 25-75% |
| Linn et al. (1982)^8^ | Southern California | Adults (n=11) | 0.47ppm /2hr/4days/re-expose week 2-6 | FEV1, FVC, symptoms | ↓FEV1 on Day 1-3, no significant decrement after; ↑symptoms on Day1-2 and week2 follow-up |
| Linn et al. (1982)^9^ | Southern California | Adults with mild-moderate COPD (n=25) | 0.12ppm/1hr | FVC, FEV1, PEER, FEF25-75, SaO2 | ↓SaO2; no association was found between O3 and other lung functions. |
| Solic et al. (1982)^10^ | North Carolina | Male adults (n=13) | 0, 0.2ppm/2hr/2consecutive days | FVC, FEV1, FEF25-75, FEF200-1200, VO2, VCO2, RER, f, VE, Raw | ↓SaO2; no association was found between O3 and other lung functions. |
| Adams and Schelegle (1983)^11^ | Davis | Adults (n=10) | 0, 0.2 or 0.35ppm /1hr with either training or competition exercises | FVC, FEV1, FEF25-75, RV | ↓FVC, ↓FEV1, ↓ FEF 25-75 after O3 exposure; no exercise mode effects were found |
| Avol et al. (1983)^12^ | Los Angeles/1980-1981 | Adults (n=158) | 0.156ppm, 0.165 ppm/1 hr | FVC, FEV1, Vmax50, CC/TLC%, ∆N2, Rt, SaO2(%) | ↓ FEV1, ↓ FVC, ↓ Vmax50 |
| Gliner et al. (1983)^13^ | Santa Barbara | Adults (n=21) | 0.20, 0.20. 0.20, 0.42, 0.5 ppm/2hr/5 consecutive days | FVC, FEV1, FEF25-75% | ↓FVC, ↓FEV1, ↓FEF25-75% only for the adaptation 0.42 or 0.50ppm exposure and acute 0.42 and 0.50ppm exposure; no significant difference between acute and adaptation exposures |
| Linn et al. (1983)^14^ | University of Southern California | Adults with COPD (n=28) | 0, 0.18,0.25ppm/1hr | FVC, FEV1, FEV3, FEV1/FVC, PEER | No association was observed between O3 exposure and reduced lung function |
| McDonnell et al. (1983)^15^ | North Carolina | Male adults (n=135) | 0, 0.12, 0.18, 0.24, 0.30, or 0.40 ppm/2.5hr | FVC, FEV1, VT, FEF25-75, SRaw, symptoms | ↓FVC, ↓ FEV1, FEF25-75 starting at 0.12ppm; ↑ Sraw, ↑ symptoms starting at 0.24ppm |
| Avol et al. (1984)^16^ | Los Angeles | Adults (n=50) | 0, 0.08,0.16, 0.24, 0.32ppm/1hr | FVC, FEV1, symptoms | ↓FVC, ↑ symptoms after exposure to concentrations of 0.16, 0.24, and 0.32ppm O3 |
| Superko et al. (1984)^17^ | Davis | Male adults with CAD (n=6 ) | 0, 0.2, 0.3ppm/40min | FVC, FEV1, FEF25-75 | No association between O3 exposure and decreased lung function was found |
| Avol et al. (1985)^18^ | Southern California | Children (n=59) | Purified air or smoggy Los Angeles ambient air /2 week apart | FVC, FEV0.75, FEV1, MMFR, PEFR, symptoms | ↓FVC, ↓FEV0.75, ↓FEV1, ↓PEFR |
| Bedi et al. (1985)^19^ | Santa Barbara | Adults (n=6) | 0.45ppm/2hr/2days apart | FRC, FVC, FEV1, FEV3, FEF25-75%, TLC | ↓FVC, ↓FEV1, ↓FEV3, ↓FEF25-75, ↓ TLC with greater decrement on Day 2 |
| Kehrl et al. (1985)^20^ | North Carolina | Male adults with COPD (n=13) | 0.3ppm/2h/2 consecutive days | FVC, FEV1 FEF50, FEF25, oxygen saturation (SaO2) | No significant association between O3 exposure and lung function, SaO2 was found |
| Koenig et al. (1985)^21^ | Seattle | Adolescents (n=20) | 0, 0.12 ppm/1hr | FEV1, FRC, Rt, Vmax50, Vmax75 | No significant decrement |
| Kulle et al. (1985)^22^ | Maryland | Adults (n=20) | 0.00, 0.10, 0.15, 0.20, and 0.25ppm/4hr | FVC, FEV1, FEF25–75, SGaw, IC, TLC, symptoms | ↓FEV1, ↑ Sgaw starting at 0.15ppm; ↑symptoms with larger effects along with higher concentration |
| Lauritzen and Adams (1985)^23^ | Davis | Female adults (n=6) | 0, 0.20, 0.30, or 0.40ppm/1hr | FVC, FEV1, FEF25-75, RV, VE | ↓FVC, ↓FEV1, FEF25-75, ↑RV, ↑VE |
| McDonnell et al. (1985)^24^ | North Carolina | Male adults (n=32) | 0.12, 0.18, 0.24, 0.30, or 0.40 ppm/2.5hr/3w-14m apart | FVC, FEV1, FEF25-75, Sraw, symptoms | ↓FVC, ↓FEV1, ↓FEF25-75, ↑SRaw, ↑symptoms, highly reproducible for as long as 10 months and for all tested O3 concentrations of 0.18 ppm or greater |
| McDonnell et al. (1985)^25^ | North Carolina | Male children (n=23) | 0, 0.12ppm/2hr | FEV1, cough symptom | ↓FEV1 and persisted for 16-20 hours |
| Gong et al. (1986)^26^ | Los Angeles | Adults (n=17) | 0, 0.12,0.20ppm/60min/separate days | FVC, FEV1, FEV1/FVC, MVV, VO2, VE, VT | ↓VE, ↓V02, ↓VT at 0.20ppm; ↓FEV1 at 0.12 and 0.20 ppm; ↑symptom mildly at 0.12ppm and larger at 0.20ppm |
| Horvath et al. (1986)^27^ | Santa Barbara | Female adults (n=10) | 0, 0.27,0.48ppm/2hr | FVC, FEV1, FEV2, FEV3, IC, FEF25-75% | ↓ FVC, ↓ FEV1, ↓ FEV2, ↓ FEV3, ↓ IC, ↓ FEF25-75% with greater decrements when co-exposed with PAN |
| Linn et al. (1986)^28^ | Los Angeles | Adults (n=24) | 0.16, 0.14, 0.12, 0.10, 0.08, and 0.00 ppm/ 2h | FVC, FEV1, PEFR, MMFR | No significant decrement |
| Schelegle and Adams (1986)^29^ | Davis | Adults (n=10) | 0, 0.12, 0.18 or 0.24ppm/1hr | FVC, FEV1, FEF25-75, symptoms | ↓FVC, ↓FEV1, ↑symptoms at 0.18 and 0.24ppm; ↓FEF25-75 at 0.24ppm |
| Avol et al. (1987)^30^ | Los Angeles | Children (n=66) | 0, 0.113ppm/1hr | FVC, FEV1, PEFR, FEF75% | ↓FVC, ↓FEV1, ↓ PEFR, ↓FEF75% with greater decrements in girls |
| Drechsler-Parks et al. (1987)^31^ | Santa Barbara | Adults (n=8) | 0.30ppm PAN + 0.45 ppm/2hr/5consecutive days | FVC, FEV1, FEF25-75, RV, TLC | ↓FVC, ↓FEV1, ↓FEF25-75 with largest decrement on Day2, no effect modification was found from exposure to PAN |
| Horvath et al. (1987)^32^ | Santa Barbara | Male adults (n=9) | 0.25ppm, 1200-1600ug/m3H2SO4/2hr | FVC, MVV, FRC, TGV, Raw | No significant decrement with O3 exposure and combination with H2SO4 |
| Kehrl et al. (1987)^33^ | North Carolina | Adults (n=9) | 0, 0.4ppm/2hr | Sraw, FVC, 99mTc-DTPA clearance | SRaw: 4.79→7.92 cmH₂O·s (Δ+3.13); p=0.04. FVC: −0.81 L (−14% from baseline); p<0.001. 99mTc-DTPA clearance: 1.75 ± 0.43 %/min; elevated vs air, p=0.03 |
| Koenig et al. (1987)^34^ | Seattle | Adults (n=20) | 0, 0.12, 0.18ppm O3 or NO /30min | PF, RT, Vmax50, Vmax75, FEV1, VC | ↓ FEV1, ↑RT |
| McDonnell et al. (1987)^35^ | North Carolina | Male adults with seasonal allergic rhinitis (n=26) | 0.18 ppm for 2 hr | FEV1, FVC, FEF25-75, Sraw, Cough, SB, PDI | ↓FEV1, ↓FVC, ↓FEF25-75, ↑Sraw, ↑Cough, ↑SB, ↑PDI |
| Bedi et al. (1988)^36^ | Santa Barbara | Older Adults (n=16) | three 0.45ppm/2hr/ average 17.2-27.3 days apart | FVC, FEV1, FEF25-75 | No significant decrements in FVC, FEV1, FEF25-75 across the three exposures; older individuals may not respond consistently to similar O3 exposures |
| Folinsbee et al. (1988)^37^ | North Carolina | Male adults (n=10) | 0, 0.12ppm/6.6hr | FEV1, FVC, FEV1/FVC, FEF25-75, FIVC, FIV0.5, MVV, SRaw, symptoms | ↓FEV1, ↓FVC, ↓FEF25-75, ↓FIVC, ↓FIV0.5, MVV, ↑SRaw, ↑ symptoms |
| Koenig et al. (1988)^38^ | Seattle | Adolescent (12 healthy, 12 asthmatic, n=24) | 0, 0.12ppm O3, 0.30ppm NO2 alone or combined/60min | FVC, FEV1, Vmax50, Vmax75, RT, FRC | ↓ Vmax50 at 0.12ppm O3 exposure in asthmatic subjects |
| Linn et al. (1988)^39^ | Los Angeles | Adults (n=25) | 2-hr exposures at ~0.18 ppm O₃ | FVC, FEV1 | ↓FEV1, ↓FVC |
| Reisenauer et al. (1988)^40^ | Seattle | Adults (n=19) | 0.0, 0.2, 0.3ppm/60min | FEV1, FVC, FRC, TLC, IC, RT | Only ↑RT observed in females |
| Bedi et al. (1989)^41^ | Santa Barbara | Adults (n=24) | 0.45 ppm/2 hr/4 days | FVC, FEV1, FEF25-75% | ↓FVC, ↓ FEV1 on 1st and 2nd day but not 3rd and 4th day |
| Brookes et al. (1989)^42^ | North Carolina | Male adults (n=15) | 0.2ppm, 0.35ppm/1hr/3 consecutive days | FVC, FEV1, FEF25-75, symptoms | ↓FVC, ↓FEV1, ↓FEF25-75, ↑symptoms; re-exposure 0.35-0.35ppm enhanced ↓FVC, ↓FEV1, ↑symptoms; re-exposure to 0.20-0.35ppm enhanced ↓FEV1 only |
| Drechsler-Parks et al. (1989)^43^ | Santa Barbara | Adults (n=32) | (1) filtered air, (2) 0.45 ppm O3, (3) 0.13ppm peroxyacetyl nitrate (PAN) +0.45 ppm 03, (4) 0.60 ppm NO2 + 0.45 ppm 03 , and (5) 0.13ppm PAN + 0.60ppm NO2 +0.45 ppm 03 /2-hour | FVC, FEV1, FEF25-75% | ↓FVC, ↓FEV1, ↓FEF25-75% after exposure to O3, O3+NO2 and PAN+NO2+O3 with greatest and earlier decrement in PAN+O3; older subjects had smaller and fewer significant decrements |
| Koren et al. (1989)^44^ | North Carolina | Male adults (n=11) | 0, 0.4ppm/2hr | Bal | ↑PMN, ↑ total protein, ↑albumin, ↑ IgG, ↑Neutrophil elastase activity, ↑PGE2 |
| Schelegle et al. (1989)^45^ | Davis | Male adults (n=20) | 0, 0.2, 0.35 ppm/80 min | FVC, FEV1, SGaw, total symptom score, and plasma PGF2a responses (eicosanoids as a marker of response to acute 03 exposure) | ↓FEV1, ↓FVC, ↑PGF2a after exposure to both O3 concentrations |
| Schonfeld et al. (1989)^46^ | Davis | Male adults (n=40) | re-exposure to 0.35 ppm/60min at intervals of 24, 48, 72, and 120 hr | FVC, FEV1, FEF25-75, Sraw, VT, symptoms | ↓ FVC, ↓ FEV1, ↑ Sraw, ↑VT, ↑symptoms |
| Drechsler-Parks et al. (1990)^47^ | Santa Barbara | Adults (n=12) | 0, 0.45ppm/1hr with continuous exercise or 2 hrs intermittent exercise | FVC, FEV0.5, FEV1, FEV3, FEF25%, FEF50%, FEF25-75% | ↓ FEV0.5, ↓FEV1, ↓FEV3, ↓FEF25%, ↓FEF50%, ↓ FEF25-75% with all 4 exposures; no difference between FEV1 decrements between two exercise protocols |
| Horstman et al. (1990)^48^ | North Carolina | Male adults (n=22) | 0.00, 0.08, 0.10, 0.12ppm/6.6hr | FVC, FEV1, FEF25-75, FEFmax, PDI, and PD100 | ↓FEV1, ↓PDI, ↓PD100 |
| Koenig et al. (1990)^49^ | Seattle | Adolescents (n=13) | (1) air +100 ppb SO2 (2) 120 ppb O3 +120 ppb O3, and (3) 120 ppb O3 + 100 ppb SO2/ 45min+15min | FEV1, RT, Vmax50 | ↓FEV1, ↑RT, ↓Vmax50 when exposure to SO2+O3 |
| Messineo and Adams (1990)^50^ | Davis | Female adults (n=14) | 0, 0.18 or 0.3ppm/1hr | FVC, FEV1, FEF25-75% | ↓FVC, ↓FEV1, ↓FEF25-75 at 0.18 and 0.30ppm; ↓FEV1 doubled in younger groups |
| Aris et al. (1991)^51^ | San Francisco | Adults (n=39) | 0.5mg/ml HNO3, H2O fog, or clean air + 0.2ppm/3hr/3 separate days | SRaw, FEV1, FVC | ↓ FEV1, ↓FVC with greater decrement in exposure to clean air/O3 |
| Emmons et al. (1991)^52^ | New York | Adults (n=34) | 0.4ppm/2hr/6-month apart | FVC, FEV1, MMF, symptoms | No association was observed between O3 exposure and reduced lung function and symptoms before smoking cessation, but ↑symptoms after 6-month cessation |
| Koren et al. (1991)^53^ | North Carolina | Adults (n=10) | 0, 0.4ppm/2hr | BalF | ↑ neutrophils, ↑ IL-6, ↑prostaglandin E were higher at 1 hr than at 18 hr post exposure; ↑ fibronectin, ↑plasminogen activator were higher at 18 hr than at 1 hr after exposure |
| McDonnell et al. (1991)^54^ | University of North Carolina | Male adults (n=38) | 0, 0.08ppm/6.6hr | FVC, FEV1, FEF25-75, Raw, Vtg, Sraw, symptoms | ↓FVC, ↓FEV1, ↓FEF25-75, ↑ Raw, ↑ Sraw, ↑ symptoms |
| Schelegle et al. (1991)^55^ | Davis | Male adults (n=20) | 0, 0.3ppm/1hr/3 separate days | FEV1, FVC, SRaw, Vtg, Bal | ↓FEV1 after 1hour and return back at 6-hr and 24-hr after O3 exposure; ↑ neutrophils in PA sample at <1-hr, 6-hr and 24-hr after O3exposure; ↑ neutrophils in DAAS sample at 6-hr and 24-hr after O3exposure |
| Hazucha et al. (1992)^56^ | North Carolina | Male adults (n=23) | triangle 0.12ppm (steady state) or 0-0.24 ppm first 4 h and back to 0/8 hr | FVC, FEV1, FEV1/FVC, FEF25-75, FEFmax | ↓FVC, ↓FEV1, ↓FEV1/FVC, ↓FEF25-75, ↓FEFmax, significant difference between triangle and square-wave exposures |
| Foster et al. (1993)^57^ | Baltimore | Male adults (n=9) | 0, 0.33ppm/2hr | VC, IC, MMF, 133Xe (ventilation), 99mTc (aerosol) | ↓VC, ↓ IC, ↓MMF, ↑ aerosol distribution to middle lung region, ↑ ventilation to upper nd middle regions and ↓ventilation to lung base |
| Fox et al. (1993)^58^ | Davis | Female adults (n=9) | 0, 0.3ppm/1hr | FVC, FEV1, FEF25-75% | ↓ FVC, ↓ FEV1, ↓ FEF25-75% with larger decrements in the F menstrual phase |
| Seal et al. (1993)^59^ | North Carolina | Adults (n=372) | 0, 0.12, 0.18, 0.24, 0.30, or 0.40 ppm/2.33hr | FEV1, Raw, Vt, symptoms | ↓FEV1 at 0.12ppm; ↑Sraw at 0.18ppm; ↑symptoms at 0.18ppm; no significant difference was found among gender-race groups |
| Basha et al. (1994)^60^ | Michigan  /Not specified | Adults (n=10) | 0 and 0.2ppm/6hr/30days apart | FVC, FEV_1_, FEV_1_/FVC, sRaw, BalF | No decrements in lung function, ↑Polymorphonuclear leukocytes (PMN), ↑IL-6, ↑IL-8 |
| Folinsbee et al. (1994)^61^ | North Carolina | Male adults (n=17) | 0.12ppm/6.6hr/5 consecutive days | FVC, FEV1, FEF25-75, SRaw, Vtg, symptoms | ↓FEV1 on Day1-4; ↑Sraw on Day1-5; ↑symptoms on Day1 only |
| Gerrity et al. (1994)^62^ | North Carolina | Adults (n=20) | 0.4ppm/1hr | FVC, FEV1, TGV, Raw, VT, VE, FURT, FLRT | ↓FVC, ↓FEV1, ↓ TGV; Initial and average VE were associated with decreased FEV1, FLRT×VE was associated with VT decrement |
| Koenig et al. (1994)^63^ | Seattle | Adolescent (n=28) | air, oxidants (0.12ppm O3+ 0.3ppm NO2), oxidants + H2SO4 70ug/m3, oxidants +0.05ppm HNO3 /90min | Fp, RT, FRC, Vmax, FVC, FEV1 | No changes in any of the pulmonary function parameters were statistically significant |
| Weymer et al. (1994)^64^ | Los Angeles | Adults (n=21) | 3 exposures to 0, 0.10 or 0.25ppm then additional to 0.4ppm/1hr | FEV1, FVC | ↓FVC, ↓FEV1, ↑symptoms at 0.40ppm concentration |
| Drechsler-Parks (1995)^65^ | Santa Barbara | Male adults (n=10) | 0 or 0.45ppm/3hr/2-week apart | MVV, FEV1, FEF25-75%, FVC, RV | ↓FVC, ↓FEV1, ↓FEF25-75, ↑RV |
| Frampton et al. (1995)^66^ | Rochester | Adults (30 healthy, 30 allergic asthmatic subjects, n=60) | 100ug/m3 H2SO4 or NaCl/3h and 0.08, 0.12 or 0.18ppm/3hr/24-hour apart | FEV1, FVC, Sgaw | No significant decrements in lung functions among healthy subjects; ↓ FVC, ↓ FEV1 among asthmatic subjects with greater decrements with NaCl and H2SO4 preexposure |
| Horstman et al. (1995)^67^ | North Carolina | Adults (17 asthmatic, 13 non-asthmatic, n=30) | 0, 0.16ppm/7.6hr | FVC, FEV1, FEVI/FVC, FEF25-75, FEFmax, SVC, IC, TLC, respiratory symptoms | ↓ FVC, ↓ FEV1, ↓ FEVI/FVC with greater decrements in asthmatics; ↓ FEF25-75, ↓ FEFmax, ↓ SVC, ↓ IC and ↑ symptoms with no difference between asthmatics and healthy subjects |
| Linn et al. (1995)^68^ | Southern California | Children with asthma (n=24) | 0ppm O3/0.2ppm O3+0.3ppm NO2/0.2ppm O3+0.3ppm NO2+100ug/m3 H2SO4/90min | FVC, FEV1, Sraw | No significant decrement with O3 exposure and combination with NO2 and H2SO4 |
| McDonnell et al. (1995)^69^ | North Carolina | Adults (n=68) | 0, 0.08, 0.10, 0.12ppm/6.6hr | FEV1 | 47% of exposed subjects experienced 10% decrement of FEV1 when exposed to 0.12ppm |
| McKittrick and Adams (1995)^70^ | Davis | Male adults (n=12) | 0, 0.30 ppm/1-hr continuous or 2-hr intermittent exercise | FVC, FEV1, FEF25-75%, symptoms | ↓FVC, ↓ FEV1, ↓FEF25-75%, ↑symptoms; significant difference in the decrements was found between continuous and intermittent exercise groups |
| Weinmann et al. (1995)^71^ | Baltimore | Adults (n=24) | 0, 0.35ppm/130min/20days apart | FEV1, FEF25-75, FEV1/FVC, FEFs, VisoV, MBNW, FRV, RV | ↓FEF25-75, ↓Vmax50, ↓Vmax75 starting after 25min O3 exposure, ↓FEV1, ↓FVC still depressed after 24-hr |
| Weinmann et al. (1995)^72^ | Baltimore | Adults (n=8) | 0, 0.35 ppm/130min | FEV1, FVC, FEF25-75, Vmax50, and Vmax75, Rp and BalF | ↑neutrophils, ↑fibrinogen, ↑albumin, ↑PGE2, ↑PGF2a; ↓FVC, ↓FEV1 after exposure at 25min but not after 24hour; ↓FEF25-75 at 25min and after 24hour |
| Balmes et al. (1996)^73^ | San Francisco | Adults (n=20) | 0, 0.2ppm/4hr/separate day | FEV1, FVC, SRaw, symptoms, Pal, BalF | ↑SRaw, ↑lower respiratory symptoms, ↑neutrophils in Pal; ↑neutrophils, ↑ total protein, ↑ Il-8 in bronchial fraction; ↑neutrophils, ↑total protein, ↑ fibronectin, ↑GM-CSF in BalF; no significant decrements in FEV1 and FVC was found |
| Devlin et al. (1996)^74^ | North Carolina | Adults (n=8) | 0, 0.4ppm/2hr | BalF | ↑IL-6, ↑ PGE2 levels were higher 1 hr than 18hr after exposure; ↑ fibronectin, tissue-plasminogen acti vator levels were higher 1h hr than 1hr after exposure; ↑ PMNs, ↑ protein, ↑ C3a same levels at both time |
| Foster et al. (1996)^75^ | Rochester | Adults (n=10) | 150 - 350 ppb for 130 min | Lung volumes, FEF25-75 | No association |
| Foster et al. (1996)^76^ | Maryland | Adults (n=9) | 150 to 350 ppb for 130 min | FEV1 | ↓FEV₁ |
| Scannell et al. (1996)^77^ | Berkely | Adults with asthma (n=18) | 0, 0.2ppm/4hr/ | FEV1, FVC, SRaw, proximal airway lavage (Pal), bronchoalveolar lavage fluid (BalF), symptoms | For asthmatic sujects: ↓FEV1, ↓FVC, ↑ Sraw, ↑lower respiratory symptoms for asthmatic sujects; ↑ neutrophils in Pal, ↑ neutrophils, ↑ total protein, ↑ Il-8 in the bronchial fraction; ↑neutrophils, ↑ total protein, ↑LDH, fibronectin, ↑↑ Il-8, ↑GM-CSF, ↑MPO in Bal |
| Balmes et al. (1997)^78^ | Berkely | Adults (n=106) | once to air and twice to 0.2ppm/4hr/3w apart | FEV1, FVC, Bal, Pal, symptoms | ↓FEV1, ↓FVC, ↑SRaw, ↑ symptoms; ↑neutrophils and total protein in bronchial fraction and Bal fluids, ↑neutrophils in Pal fluid, which are correlated with O3-induced increases in sRaw, but not with O3-induced declines in FEV1 or FVC |
| Frampton et al. (1997)^79^ | Rochester | Adults (n=90) | 0.22ppm/4hr | FEV1, FVC, FEF25-75 | ↓FEV1 with smaller effects in smokers; pack-yr of smoking to be associated with decreased O3 responsiveness (OR=0.87, P = 0.017) |
| Frampton et al. (1997)^80^ | Rochester | Adults (56 nonsmokers, 34 smokers, n=90) | 0, 0.22, 0.22ppm/4hr/3-week apart | FEV1, Bal, NL | ↑ IL-6, ↑ IL-8, ↑ PMNs, ↑lymphocytes, ↑ mast cells in Bal with no significant difference between 2 groups; ↓FEV1 with smaller effects in smokers |
| Gong et al. (1997)^81^ | Los Angeles | Adults with asthma (n=10) | 0.4 ppm/3hr/5 consecutive days | FVC, FEV1, Bronchial reactivity | % change: FEV₁= -35% (Day 3), -34% (Day 4), -6% (Day 7), -15% (Day 8, 4-day follow-up), -17% (Day 9, 7-day follow-up); FVC=-24.7% (day 3), -24.3% (day 4), -12.4% (day 5), -10.3% (day 6), -3.6% (day 7), -10.5% (day 8), -11.7% (day 9); Bronchial reactivity=Not specified |
| McDonnell et al. (1997)^82^ | North Carolina | Adults (n=485) | 0.0, 0.12, 0.18, 0.24, 0.30, or 0.40 ppm/2hr | percent decrement (100% ×[pre - post]/pre) in FEV1 (DELFEV1), FEV1, VE | Not specified; ↓FEV1, ↑ DELFEV1 along with increasing O3 concentration (C), minute ventilation (E) during exposure, and duration of exposure (T). |
| Peden et al. (1997)^83^ | North Carolina/1995-1996 | Adults (n=8) | 0.16 ppm/7.6hr | FVC, FEV1, percentage of eosinophils recovered in BalF and ILF after exposure to air and ozone | % change: FVC=−9.16%; FEV1=−13.64%; significant increases in airway eosinophils, especially in bronchial lavage fluid |
| Christian et al. (1998)^84^ | San Francisco | Adults (n=15) | 0.2ppm/4hr/1 day or 4 consecutive days | FEV1, FVC, SRaw, BalF, symptoms | Day 1-3: ↓ FVC, ↓ FEV1 (day 1: p<0.001, day 2: p<0.0001, day 3: p<0.024);↓ IL-6 after 4 days; ↑symptoms at Day1-2 |
| Gong et al. (1998)^85^ | California | Adults (10 nonmedicated hypertensive, 6 healthy males, n=16) | 0.3ppm/3hr | FEV1, FVC, alveolar–arterial oxygen tension difference (AaPO2) | All subjects (n=16): FEV₁ 3.47→3.26 L (≈−6.0%) ‡ p<0.01; FVC 4.37→4.13 L (≈−5.5%) † p<0.05; SaO₂ 96.2→94.7% (−1.5 pp); A–aPO₂ 14.6→25.4 mmHg (+10.8) ‡ p<0.01. Hypertensive (n=10): FEV₁ 3.41→3.20 L (p<0.05); FVC 4.35→4.08 L; AaPO₂ 16.6→26.8 mmHg (p<0.05); Controls (n=6): FEV₁ 3.58→3.36 L (p<0.01); FVC 4.41→4.21 L (p<0.05); AaPO₂ 11.2→22.9 mmHg (p<0.05) |
| Gong et al. (1998)^86^ | Glendora (Los Angeles)/1986-1987 | Adults (n=164) | 0, 0.4ppm/2hr | FEV1, FVC | % change: FEV1=-6.8% (p<0.05), FVC=-5.9% (p<0.05); No association between chronic lung function changes and acute responses to O3 was found |
| Frampton et al. (1999)^87^ | Rochester | Adults (n=27) | 0, 0.22ppm/4hr | Aldehydes products from BalF | C9: increment (p=0.0001), C6: Not significant (p=0.16); No significant relationships of C6/C9 with pulmonary function changes, PMN, total protein, albumin, estimated O₃ dose, age, or sex |
| Kehrl et al. (1999)^88^ | North Carolina | Adults (n=9) | 0, 0.16ppm/7.6hr/4-week apart | FEV1 | Mean decrement in FEV1=-9.1% (p<0.01) |
| McDonnell et al. (1999)^89^ | North Carolina | Male adults (n=485) | 0.0, 0.12, 0.18, 0.24, 0.30, or 0.40 ppm/2hr | FEV1, symptoms | Prevalence ratios of 15% decrement of FEV1 and development of symptoms; Cough: PR=2.20 (95% CI 1.53–3.17); Shortness of breath: PR=2.08 (95% CI 1.46–2.97); Pain on deep inspiration (PDI): PR=1.75 (95% CI 1.28–2.40) |
| Adams (2000)^90^ | Davis | Adults (n=30) | 0, 0.12ppm/6hr | FEV1, FVC, FEF25-75% | ↓FEV1, ↓FVC, ↓FEF25-75% |
| Folinsbee and Hazucha (2000)^91^ | North Carolina | Female adults (n=19) | 0, 0.35ppm/75min | FEV1, FVC, FEF25-75%, PEF, FIVC (forced inspiratory vital capacity), FIV0.5 (inspired volume during the first 0.5 s of an FIVC maneuver), PIF (peak inspiratory flow), airway resistance (PC100Raw) | FVC Air pre 3.89±0.16, post 3.90±0.16, 18h 3.93±0.16, 42h 3.88±0.15; O3 pre 3.94±0.15, post 3.42±0.17 (p<0.01), 18h 3.85±0.15 (p<0.05), 42h 3.87±0.15 (NS); FEV1 Air pre 3.18±0.12, post 3.18±0.14, 18h 3.19±0.13, 42h 3.13±0.12; O3 pre 3.21±0.12, post 2.57±0.15 (p<0.01), 18h 3.05±0.12 (p<0.01), 42h 3.12±0.12 (NS); FEV1/FVC Air pre 82.4±2.1, post 82.2±2.0, 18h 81.9±2.0, 42h 81.5±2.0; O3 pre 82.1±2.1, post 76.1±3.0 (p<0.01), 18h 80.0±2.5 (NS), 42h 81.6±2.3 (NS); FEF25–75 Air pre 2.05±0.15, post 2.03±0.16, 18h 2.02±0.16, 42h 1.96±0.16; O3 pre 2.04±0.17, post 1.43±0.12 (p<0.01), 18h 1.86±0.17 (p<0.05), 42h 1.96±0.17 (NS); PEF Air pre 7.05±0.35, post 6.98±0.36, 18h 7.21±0.29, 42h 7.11±0.30; O3 pre 7.31±0.34, post 5.64±0.45 (p<0.01), 18h 6.92±0.32 (p<0.05), 42h 7.07±0.32 (NS); MVV Air pre 117.5±4.1, post 116.0±3.8; O3 pre 119.9±3.9, post 100.3±4.6 (p<0.01); Raw* Air pre 1.58±0.16, post 1.53±0.14, 18h 1.72±0.18, 42h 1.63±0.15; O3 pre 1.48±0.11, post 1.73±0.14 (p<0.05), 18h 1.84±0.16 (p<0.05), 42h 1.63±0.14 (NS); TGV* Air pre 2.72±0.12, post 2.83±0.12, 18h 2.79±0.13, 42h 2.65±0.09; O3 pre 2.80±0.13, post 2.80±0.11, 18h 2.76±0.15, 42h 2.72±0.14 (NS all); PC100 Raw* Air post 42.3±9.9, 18h 35.3±8.9, 42h 40.4±16.9; O3 post 18.6±4.9 (p<0.05), 18h 22.7±5.6, 42h 35.7±10.5 \|\| Forced Inspiratory (n=12) — FIVC Air pre 3.89±0.19, post 3.90±0.19, 18h 3.84±0.19, 42h 3.84±0.19; O3 pre 3.86±0.18, post 3.45±0.18 (p<0.01), 18h 3.72±0.20 (NS), 42h 3.72±0.20 (NS); FIV0.5 Air pre 2.43±0.15, post 2.45±0.14, 18h 2.44±0.12, 42h 2.42±0.13; O3 pre 2.55±0.16, post 2.02±0.18 (p<0.01), 18h 2.33±0.13 (p<0.05), 42h 2.46±0.11 (NS); FIV1 Air pre 3.74±0.17, post 3.76±0.18, 18h 3.70±0.18, 42h 3.71±0.17; O3 pre 3.72±0.18, post 3.15±0.20 (p<0.01), 18h 3.59±0.18 (NS), 42h 3.59±0.17 (NS); FIV1/FIVC Air pre 96.8±0.7, post 96.6±1.1, 18h 97.1±1.2, 42h 96.9±1.1; O3 pre 96.8±2.1, post 91.8±4.3 (NS), 18h 96.8±1.1 (NS), 42h 97.1±1.1 (NS); PIF Air pre 6.41±0.36, post 6.35±0.34, 18h 6.34±0.30, 42h 6.21±0.31; O3 pre 6.69±0.40, post 5.31±0.48 (p<0.01), 18h 6.19±0.32 (p<0.05), 42h 6.43±0.29 (NS) |
| Foster et al. (2000)^92^ | Baltimore | Adults (n=9) | 0, 120-240ppb/130min | FEV1, FVC | <130-min O₃ (mean 175 ppb) with exercise at 22 °C/30 °C> rapid–shallow breathing (Vₜ ↓ vs FA; f ↑); FEV₁/FVC ↓ ~2–7% mid/end exposure; at ~24 h FEV₁ still −2.3% ^93^ while FVC recovered |
| Frank et al. (2001)^94^ | Baltimore | Adults (n=8) | 0, 0.25ppm/2hr/4 consecutive days | Small airway function (SAWgrp): FEF25-75, Vmax50, Vmax75 | ↓FEF25-75, ↓ Vmax50, ↓Vmax75 on Day2-4 |
| Hazucha et al. (2003)^95^ | North Carolina | Adults (n=240) | 0.42, 0.4ppm ppm/1.5hr/1week-several months apart | FEV1 | <Cohorts (post-O₃ %FEV₁ change, mean [range])> Young males −16.3% [+1 to −44], Young females −16.6% [+2 to −53], Middle-aged males −11.6% [0 to −63], Middle-aged females −6.4% [+2 to −28]; middle-aged females vs all others p<0.005; middle-aged males vs young cohorts p<0.05 |
| Chen et al. (2004)^96^ | San Francisco | Adults (n=14) | 0, 0.2ppm/1hr/separate days | FEV1, FVC, cellular and biochemical markers, symptoms, concentration of DF causing a 15% decrease in FEV1 from the post-saline solution baseline (PC15) | <FEV1 (1-h exercise)> O3 −10% vs Air −4% (NS); <FVC & SRaw (1-h)> NS vs Air; <Lower respiratory symptoms> ↑ after O3 vs Air (significant); <Allergen sensitivity (PC15, AU/mL)> median O3 24 (3–723) vs Air 31 (3–3276), 9/14 lower after O3 (group NS); <Lavage (6 h post-allergen)> PAL neutrophils ↑ trend after O3 (p=0.06); no O3–Air differences in eosinophils, lymphocytes, total leukocytes, total protein, fibronectin, GM-CSF, IL-5, IL-6, IL-8, TGF-β1/β2, ECP, tryptase, MPO; biopsy morphometry: no differences. |
| Arjomandi et al. (2005)^97^ | San Franciso | Adults with asthma (n=14) | 0.2ppm/4hr/1 or 4 consecutive days | FVC, FEV1, FEF25-75, BalF, symptoms | <FEV1 (ΔL pre→post)> 1-D −0.5±0.4 (p=0.001); MD1 −0.6±0.6 (p=0.002); MD2 −0.9±0.7 (p<0.001); MD3 −0.5±0.5 (p=0.004); MD4 −0.1±0.2 (p=0.017); <FVC (ΔL)> 1-D −0.4±0.4 (p=0.001); MD1 −0.6±0.5 (p=0.001); MD2 −0.9±0.7 (p=0.001); MD3 −0.5±0.5 (p=0.004); MD4 −0.1±0.2 (p=0.036); <FEF25–75 (Δ L/s)> 1-D −0.7±0.9 (p=0.012); MD1 −0.9±1.2 (p=0.010); MD2 −1.1±0.8 (p<0.001); MD3 −0.7±0.8 (p=0.011); MD4 −0.1±0.4 (p=0.271); <FEF75 (Δ L/s)> 1-D −0.4±0.5 (p=0.011); MD1 −0.5±0.5 (p=0.001); MD2 −0.5±0.5 (p=0.001); MD3 −0.3±0.5 (p=0.026); MD4 0.0±0.2 (p=0.503); <Lower respiratory symptoms> ↑ at 1-D (p=0.012), MD1 (p=0.001), MD2 (p=0.001); NS at MD3 (p=0.229), MD4 (p=0.950). |
| Brown et al. (2008)^98^ | North Carolina | Adults (n=31) | 0, 0.06, 0.08ppm/6.6hr | FEV1, FVC, VT | ↓FEV1 |
| Schelegle et al. (2009)^99^ | Davis/2012-2019 | Adults (n=31) | 0, 60, 70, 80, and 87 ppb/6.6hr | FVC, FEV1, FEV_1_/FVC, total symptom scores | <FEV1 (%Δ from baseline)> 60 ppb: NS (max diff vs FA at 6.6 h −3.52 ± 1.52%); 70 ppb: ↓ significant at 6.6 h; 80 ppb: ↓ significant after 4th–6th periods & +1 h; 87 ppb: ↓ significant after 5th–6th periods & +1 h; <FVC (%Δ)> 60 ppb: NS; 70 ppb: NS; 80 ppb: ↓ significant after 4th & 6th periods; 87 ppb: ↓ significant after 5th–6th periods & +1 h; <FEV1/FVC (%Δ)> 60–80 ppb: NS; 87 ppb: ↓ significant after 5th–6th periods; all pulmonary function measures returned to baseline by +4 h. <TSS (total symptom score)> 60 ppb: NS at all time points; 70 ppb: ↑ significant after 5th–6th exercise periods; 80 ppb: ↑ significant after 5th–6th periods; 87 ppb: ↑ significant after 5th–6th periods; all returned to baseline by +4 h. |
| Madden et al. (2014)^100^ | North Carolina | Adults (n=15) | 0.3ppm/2hr/2 consecutive days | FEV1, FVC | <FEV1 (% change from pre)> Day1: Air ≈0, DE ≈0, O3 −9.9±2.5, DE+O3 −17.6±4.6; Day2 (all O3): after Air −10.9±2.6, after DE −14.7±3.3, after O3 −18.2±4.5 (Day1 O3 −10.3±3.3), after DE+O3 −13.8±2.2; <FVC (% change from pre)> Day1: Air ≈0, DE ≈0, O3 −8.7±2.3, DE+O3 −11.8±3.0; Day2 (all O3): after Air −7.8±1.8, after DE −9.2±1.9, after O3 ≈−7.8±1.8 (≈Day1 O3 −8.7), after DE+O3 similar; all returned to baseline by 4 h. |
| Kariisa et al. (2015)^101^ | Ohio/1998-2002 | Adults with COPD (n=1,218) | Average daily level with lag 0-3 and cumulative and average 6-year exposure (10 ppb) | FEV1, FVC, St. George’s respiratory score | <Post-BD FEV1%> Average: β=-0.2270 (p=0.1600), cumulative: β=-0.000650 (p<.0001), same-day: β=+4.9400 (p=0.4500), 3-day lag: β=-9.2700 (p=0.1700); <Post-BD FVC%> Average: β=-0.4990 (p=0.0680), cumulative: β=-0.000630 (p<.0010), same-day: β=+7.2000 (p=0.5200), 3-day lag: β=-5.7500 (p=0.6100); <SGRQ> Average: β=-0.3460 (p=0.1900), cumulative: β=+0.000945 (p<.0010), same-day: β=+0.0540 (p=0.6280), 3-day lag: β=+0.0210 (p=0.8500) |
| Habre et al. (2018)^102^ | Los Angeles/2014, 2015 | Non-smoking adults with mild to moderate asthma (n=22) | two occasions in public parks inside (exposure) and outside (control) of the high UFP zone | FENO, FEV1, FVC, MMEF, IL6, sTNFrII, PEFR | IL-6: 0.03 (-0.20, 0.27), sTNFrII:-52.30 (-91.09, -1), FEV1: 1.34 (-0.05, 2.73), FVC: 0.28 (-1.09, 1.66), MMEF: 0.48 (-3.97, 4.92), PEFR: 2.75 (-2.64, 8.13), FENO: -0.02 (-0.07, 0.03) |
| Rich et al. (2020)^103^ | Rochester, Chapel Hill, San Francisco | Adults (n=77) | 0, 70, 120 ppb/3hr | FEV1, FVC | ↓ FEV1, ↓FVC with greater decrements when co-exposed to NO2 and CO |
| Wilgus et al. (2025)^104^ | USA/2017-2020 | Adults with HIV (n=1411) | 1-year average (3ppb) | FEV1, FVC, FEV1/FVC, % predicted diffusing capacity for carbon monoxide (DLCO), respiratory symptom score | MACS-PWoH: FEV1/FVC: predicted DLCO :2.39% (95% CI: 0.65 -4.14), MACS-PWH: FEV1/FVC: 0.013 (95%CI: 0.002-0.023); WIHS-PWoH: SGRQ: -1.72 (95%CI: -3.13--0.31) |
| **Observational Studies** | | | | | |
| **Author (year)** | **Study Region/year** | **Study Population** | **Exposure Metrix** | **Health Outcome** | **Effect Estimates** |
| Lebowitz et al. (1985)^105^ | Tucson/1979-1980 | Adults (n=229) | Average hourly maximum level (Not specified) | PEF | <PEF> Young: ANOVA F=2.97 (df=29,644, p<0.001); adjusted PEF=0.287−6.025·O₃ (n=674, p=0.013; intercept & slope p<0.001); w/o TSP + add RH: R=−0.56 (p<0.0001, n=1,513), adjusted PEF=0.17−3.87·O₃ (Residual PEF p<0.002).  <PEF> Adults: Asthmatic cluster O₃×Temp ANOVA F(8,312)=9.25 (p<0.0001, R²=19.2%); PEF z by O₃ (<0.038/0.038–0.051/≥0.052 ppm): <61°F −1.94/−1.99/−2.11; 61–80°F −1.85/−1.78/−1.71; 80–96°F −2.17/−1.75/−1.57>. AOD group: ANOVA F=3.84 (df=15,243, p<0.001); β(O₃)=−5.946 (p<0.005); PEF z by O₃ terciles <0.038 −0.581, 0.038–0.051 −0.582, 0.052–0.12 −0.801> (t-test p<0.01; overall ANOVA F(15,349)=8.7, p<0.0001; n(days)=586). |
| Lioy et al. (1985)^106^ | New Jersey | Children (n=39) | Peak hour O3 concentration | FVC, FEV1, PEFR, MMEF | ↓ FVC, ↓ FEV1, ↓ PEFR |
| Lebowitz et al. (1987)^107^ | Arizona/1972-1974 | Adults (n=204) | Daily maximum level (Not specified) | daily peak flow, symptoms | Not specified(↓daily peak flow, ↑symptoms) |
| Vedal et al. (1987)^108^ | Pennsylvania/1980-1981 | Children (n=4,000) | Daily average level (Not specified) | morning PEER and Incidence rate of wheeze symptoms, upper respiratory illness (URI), lower respiratory illness (LRI) | No association was found between ozone exposure and symptoms and PEEF |
| Spektor et al. (1988)^109^ | New York/1985 | Adults (n=30) | Daily average level (Not specified) | FEV1, FVC, PEFR, FEF25-75 | <Respiratory function slopes> Full (n=30): FVC −2.08 ± 0.46 ml/ppb, FEV₁ −1.35 ± 0.35 ml/ppb, PEFR −9.21 ± 1.69 ml/s/ppb, FEF₂₅–₇₅ −6.00 ± 1.04 ml/s/ppb, FEV₁/FVC −0.0376 ± 0.0084 %/ppb; Male (n=20): FVC −2.45 ± 0.65, FEV₁ −1.86 ± 0.73, PEFR −12.07 ± 2.14, FEF₂₅–₇₅ −6.28 ± 1.49, FEV₁/FVC −0.0410 ± 0.0122; Female (n=10): FVC −1.35 ± 0.41, FEV₁ −1.31 ± 0.44, PEFR −3.47 ± 1.72, FEF₂₅–₇₅ −5.44 ± 1.01, FEV₁/FVC −0.0309 ± 0.0068. |
| Dockery et al. (1989)^110^ | 6 U.S. cities/1980-1981 | Children (n=5,422) | Daily average level (Not specified) | FEV1, FEV0.75, FVC, MMEF, symptoms | No association between O3 and any lung function or symptoms was found |
| Kinney et al. (1989)^111^ | Kingston and Harriman, Tennessee/1981 | Children (n=154) | 1h -max in the previous 24hr of measurement (Not specified) | FVC, FEV75, MMEF, Vmax75 | <FVC> −0.92 (0.36)* ml, <FEV₇₅> −0.99 (0.36)† ml, <MMEF> −1.9 (0.88)* ml/s, <Vmax₇₅> −2.4 (0.78)† ml/s; No difference in the decrements related to sex, asthma status, respiratory illness before 2 yr of age, or the MMEF/FVC ratio (a rough surrogate for airway size) |
| Berry et al. (1991)^112^ | New Jersey/1988 | Adolescents (n=34) | 1-h average before the test and 8-h average for the day of test (Not specified) | FVC, FEV1, PEF, symptoms | 1-h O₃ average – Rec-camp workers: <FEV₁> 0.57 (0.36), <FVC> 0.002 (0.68), <PEFR> 1.63 (2.23); Y-Camp workers: <FEV₁> 0.46 (0.49), <FVC> 0.84 (0.31), <PEFR> 0.98 (0.98); Y-Camp campers: <FEV₁> 0.82 (0.33), <FVC> 0.83 (0.38), <PEFR> −1.01 (1.17). 8-h O₃ average – Rec-camp workers: <FEV₁> 0.52 (0.70), <FVC> −0.19 (0.78), <PEFR> 0.83 (4.10); Y-Camp workers: <FEV₁> 0.21 (1.03), <FVC> 0.27 (0.99), <PEFR> 0.60 (1.23); Y-Camp campers: <FEV₁> 0.89 (0.42), <FVC> 1.29 (0.49), <PEFR> −2.35* (1.44) |
| Brunekreef et al. (1991)^113^ | Ohio, Tennessee, Pennsylvania/Not specified | Children (n=410) | Average on the day or days before measurement | FEV1, FVC, FEV75% | Kingston — O₃ (max hour): <FVC> 1.45 (p = 0.05), <FEV₀.75/1.0> 1.30 (p = 0.05). Fairview Lake — O₃ (1 h): <FVC> 1.20 (p = 0.01), <FEV₀.75/1.0> 1.57 (p = 0.01); (2 h): <FVC> 1.16 (p = 0.01), <FEV₀.75/1.0> 1.54 (p = 0.01); (4 h): <FVC> 1.17 (p = 0.01), <FEV₀.75/1.0> 1.46 (p = 0.01). |
| Spektor et al. (1991)^114^ | New Jersey/1988 | Healthy children (n=46) | Previous day mean level, 1-hr maximum level, 1-hr mean level | FVC, FEV1, FEF25-75, PEFR | <Exposure (Fairview Lake 1988)> 1-h O₃ exceeded 120 ppb on 5 days (max 150 ppb); 12-h H⁺ >10 µg/m³ on 7 days (max 18.6 µg/m³ on Jul 27); max THI 81 °F; correlations with 1-h peak O₃: prev 1-h O₃–PM r=0.89, prev 1-h O₃–AM r=0.77, avg O₃ (AM–PM) r=0.87, H⁺ r=0.56, THI r=0.31; for avg O₃ (AM–PM): r with O₃ in hour before PM=0.79 vs before AM=0.46; analyses on 46 children (≥7 days), carryover subset n=35; rainy periods excluded. |
| Krzyzanowski et al. (1992)^115^ | Arizona/1986-1988 | Children and Adults (n=287 children and 523 adults) | (a) daily 1-h max and the 8-h max moving average and (b) the 8-h average max from values obtained on preceding days, including the mean of these values for the previous 4 d. | PEFR | Model I (1-h max): O₃ × evening = −3.7 ± 3.1; O₃ × time out × asthma × evening = −2.3 ± 1.4 (p<0.05); constant = 566.3 ± 4.8; females = −145.2 ± 4.8; evening = 14.7 ± 1.9; asthma × morning = −7.8 ± 2.0; female × evening = −2.8 ± 1.0. Model II (5-h mean): O₃ × evening = −3.8 ± 3.6; O₃ × time out × asthma × evening = −2.9 ± 1.6 (p<0.05); environmental tobacco smoke = −16.4 ± 7.2; constant = 567.1 ± 4.9; females = −145.3 ± 6.7; evening = 14.4 ± 1.8; asthma × morning = −7.8 ± 1.9; female × evening = −2.8 ± 1.0. Model III (time-dependent): O₃ × time out × asthma × evening = −4.5 ± 1.8 (p<0.05); O₃ × time out × evening = 0.3 ± 0.05; constant = 571.1 ± 5.6; females = −143.9 ± 6.9; evening = 12.0 ± 0.9; asthma × morning = −9.8 ± 2.0; female × evening = −2.3 ± 1.1. |
| Neas et al. (1995)^116^ | Uniontown (Pennsylvania)/1990 | Children (n=98) | 12-hour average level (Not specified) | PEFR, FEV1, FVC, symptoms | mean ΔPEFR −2.8 L/min (95% CI −6.7, 1.1) |
| Kinney et al. (1996)^117^ | Governors Island (NY)/1992 | Adults (n=19) | summer (1992-S1, 1993-S2) and winter (1992-W) season average (Not specified) | Bal | BAL ROS release ↓ (p=0.03); BAL LDH ↑ ~2× (p=0.02); BAL IL-8 ↑ (p=0.12); BAL PGE₂ ↑ (p=0.06) |
| Raizenne et al. (1996)^118^ | U.S.A. and Canada /1998-1991 | Children (n=10,251) | annual average of 24-hour (18.5 ppb), daytime (39.4 ppb), 1-hr max (45.6 ppb) | FEV1, FVC, FEV0.75, FEF25-75%, PEFR | % Decrease: <24-hr> FVC -2.87% (-4.82%, -0.88%), FEV1 0.00% (0.00%, 0.00%), FEV0.75 -2.84% (-4.66%, -0.98%), FEF25–75% -4.00% (-7.35%, -0.53%), PEFR -0.52% (-4.80%, 3.96%); <Daytime> FVC -3.74% (-6.45%, -0.94%), FEV1 -2.93% (-4.80%, -1.02%), FEV0.75 -3.23% (-5.91%, -0.47%), FEF25–75% -3.98% (-8.89%, 1.20%), PEFR +0.96% (-5.00%, 7.29%); <1-hr max ozone> FVC -3.57% (-6.39%, -0.67%), FEV1 -3.29% (-6.10%, -0.39%), FEV0.75 -2.94% (-5.74%, -0.06%), FEF25–75% -3.49% (-8.58%, 1.89%), PEFR +1.07% (-4.99%, 7.50%) |
| Kunzli et al. (1997)^119^ | Berkeley/1995 | Adults (n=130) | Lifetime residential exposure (10 ppb) | FVC, FEV1, FEF25–75%, FEF75% | Hours >60 ppb (h/month): (i) Time–activity (SD=18.8): <FVC> 0.0 (0.1), <FEV₁> −0.092 (0.085), <FEF₂₅–₇₅> −0.326 (0.168, p<0.05), <FEF₇₅%> −0.252 (0.117, p<0.10), <N₂-slope> 0.033 (0.075). ^101^ Time outdoors (SD=10.5): <FVC> −0.167 (0.111), <FEV₁> −0.195 (0.096, p<0.05), <FEF₂₅–₇₅> −0.361 (0.192, p<0.05), <FEF₇₅%> −0.242 (0.134, p<0.10), <N₂-slope> 0.017 (0.086). ^120^ Ecologic (SD=30.8): <FVC> −0.148 (0.112), <FEV₁> −0.179 (0.096, p<0.05), <FEF₂₅–₇₅> −0.348 (0.193, p<0.05), <FEF₇₅%> −0.241 (0.134, p<0.10), <N₂-slope> 0.008 (0.086). (iv) Ecologic, ages ≤12 (SD=28.3): <FVC> +0.004 (0.090), <FEV₁> −0.048 (0.080), <FEF₂₅–₇₅> −0.196 (0.156), <FEF₇₅%> −0.190 (0.108, p<0.10), <N₂-slope> −0.045 (0.067). (v) Ecologic, ages <6 (SD=4.3): <FVC> −0.137 (0.116), <FEV₁> −0.196 (0.099, p<0.05), <FEF₂₅–₇₅> −0.416 (0.200, p<0.10), <FEF₇₅%> −0.262 (0.139, p<0.10), <N₂-slope> +0.060 (0.080).  10 AM–6 PM ppb·hr/day: (i) Time–activity (SD=89.6): <FVC> +0.066 (0.074), <FEV₁> −0.030 (0.065), <FEF₂₅–₇₅> −0.224 (0.128, p<0.05), <FEF₇₅%> −0.190 (0.089, p<0.10), <N₂-slope> −0.052 (0.056). ^101^ Time outdoors (SD=40.8): <FVC> −0.039 (0.102), <FEV₁> −0.105 (0.087), <FEF₂₅–₇₅> −0.320 (0.174, p<0.05), <FEF₇₅%> −0.243 (0.121, p<0.10), <N₂-slope> −0.079 (0.079). ^120^ Ecologic, ppb (SD=14.8): <FVC> −0.020 (0.103), <FEV₁> −0.092 (0.089), <FEF₂₅–₇₅> −0.311 (0.176, p<0.05), <FEF₇₅%> −0.247 (0.122, p<0.10), <N₂-slope> −0.089 (0.079). (iv) Ecologic, last residence, ppb (SD=12.7): <FVC> +0.109 (0.090), <FEV₁> +0.003 (0.080), <FEF₂₅–₇₅> −0.236 (0.153), <FEF₇₅%> −0.253 (0.105, p<0.05), <N₂-slope> −0.100 (0.067). (v) Ecologic, ages <6 (SD=18.1): <FVC> −0.024 (0.105), <FEV₁> −0.115 (0.091), <FEF₂₅–₇₅> −0.360 (0.180, p<0.05), <FEF₇₅%> −0.260 (0.125, p<0.10), <N₂-slope> −0.015 (0.080). |
| Galizia et al. (1999)^121^ | New Haven/1995 | Adults (n=520) | 10-year average summer-season daily 1-hr maximum ozone levels (Not specified) | FEV1, FVC, FEF25-75, FEF75, symptoms | % Difference: ↓FEV1=-3.07% (-5.92%, -0.22%), FVC=-1.06% (-3.96%, 1.84%), ↓FEF25-75=-8.11% (-13.90%, -2.32%), FEF75=-6.73% (-14.83%, 1.37%), ↑symptoms with greater change observed among males |
| Naeher et al. (1999)^122^ | Virginia/1994-1996 | Female adults (n=473) | 24-h average, 24-h max, 24-h min, 8-h average, 12-h avg, running 8-h average, and running 8-h max (30 ppb) | PEF | <Morning PEF> Lag 0: β -0.051, ΔPEF% -0.33, p 0.257; Lag 1: β -0.071, ΔPEF% -0.47, p 0.105; Lag 3: β -0.028, ΔPEF% -0.18, p 0.488; 3-day avg: β -0.120, ΔPEF% -0.78, p 0.081; Max 8-h avg: β -0.019, ΔPEF% -0.13, p 0.491 <Evening PEF> Lag 0: β -0.083, ΔPEF% -0.54, p 0.051; Lag 1: β -0.054, ΔPEF% -0.35, p 0.219; Lag 3: β -0.102, ΔPEF% -0.66, p 0.024; 3-day avg: β -0.151, ΔPEF% -0.99, p 0.027; Max 8-h avg: β -0.033, ΔPEF% -0.22, p 0.202 |
| Peters et al. (1999)^123^ | Southern California/1993-1994 | Children (n=3,600) | 24-hour average O3 and average daily 1-hour maximum O3 (40 ppb) | FVC, FEV1, MMEF, PEFR | FVC: male asthmatic +94.4 (SE 49.0), male nonasthmatic +35.1 (27.8), female asthmatic −248.8 (78.5), female nonasthmatic +6.4 (35.9); FEV₁: male asthmatic +107.4 (69.5), male nonasthmatic +40.0 (23.4), female asthmatic −135.0 (57.2), female nonasthmatic −16.9 (24.0); PEFR: male asthmatic +614.1 (189.8), male nonasthmatic +47.5 (67.8), female asthmatic −438.6 (270.6), female nonasthmatic −144.3 (58.4); MMEF: male asthmatic +37.7 (162.0), male nonasthmatic +31.5 (24.8), female asthmatic +162.3 (216.2), female nonasthmatic −129.6 (46.8). |
| Gauderman et al. (2000)^124^ | Los Angeles/1993-1997 | Children in 4th, 7th and 10th grade (n=3,035) | Annual average level (10-6: 38.6 ppb, overall: 55 ppb), | FEV1, FVC, maximal mid expiratory flow (MMEF), FEF75% | O₃ (10–6): FVC 4th −0.22% (−0.79, 0.36); 7th −0.10% (−0.68, 0.47); 10th 0.11% (−0.84, 1.07) \| FEV₁ 4th −0.19% (−0.99, 0.62); 7th 0.20% (−0.41, 0.81); 10th 0.24% (−1.03, 1.54) \| MMEF 4th −0.24% (−1.41, 0.95); 7th −0.37% (−2.20, 1.50); 10th 0.29% (−3.50, 4.23) \| FEF₇₅ 4th −0.85% (−2.38, 0.70); 7th −0.31% (−1.95, 1.35); 10th 0.49% (−3.36, 4.49) \|\| O₃ (24 h): FVC 4th 0.17% (−0.79, 1.15); 7th 0.39% (−0.51, 1.29); 10th 0.03% (−1.57, 1.65) \| FEV₁ 4th 0.56% (−0.73, 1.87); 7th 0.83% (−0.12, 1.79); 10th 0.79% (−1.33, 2.95) \| MMEF 4th 0.96% (−0.84, 2.79); 7th 0.51% (−2.45, 3.56); 10th 0.35% (−5.94, 7.07) \| FEF₇₅ 4th 0.69% (−1.88, 3.32); 7th 0.38% (−2.13, 2.95); 10th 1.08% (−5.34, 7.92).; No association was found between O3 exposure and any lung function |
| Kinney and Lippman (2000)^125^ | Fort Dix, Fort Benning, Fort Leonard Wood, Fort Sill/1990 | Adults (n=83) | Daily 1-hr maxima (5-wk summer, Not specified) | FVC, FEV1, FEF25-75 | High (Fort Dix): ΔFEV₁ −0.078 L (SE 0.041), ΔFVC −0.006 L (SE 0.052), ΔFEF₂₅–₇₅ −0.173 L/s (SE 0.104) \| Moderate (Forts Benning/Leonard Wood/Sill): ΔFEV₁ −0.031 L (SE 0.024), ΔFVC +0.024 L (SE 0.030), ΔFEF₂₅–₇₅ −0.075 L/s (SE 0.076) \| All subjects: ΔFEV₁ −0.0445 L (SE 0.021), ΔFVC +0.015 L (SE 0.026), ΔFEF₂₅–₇₅ −0.103 L/s (SE 0.062) |
| Mortimer et al. (2000)^126^ | 8 urban cities (Bronx, East Harlem, Baltimore, Washington, Detroit, Cleveland, Chicago, St. Louis)/1993 | Children (n=846) | Daily 8-h max average level with lags (15 ppb) | PEFR, incident symptoms | Morning %PEFR: Normal/term—Female +0.38 (−0.36, 1.11); Male −0.64 (−1.22, −0.06); Black −0.40 (−1.33, 0.53); Hispanic −0.84 (−1.65, −0.03); Atopy 0 −0.91 (−1.85, 0.04); 1–3 −0.12 (−0.86, 0.62); ≥4 +0.29 (−0.47, 1.05); No meds −0.06 (−1.10, 0.98); β-agonist/xanthines −0.43 (−1.11, 0.25); Cromolyn no steroids −1.34 (−2.70, 0.01); Steroids −0.13 (−0.86, 0.61); Crowding ≤1.0 +0.05 (−0.66, 0.76); >1.0 −0.57 (−1.16, 0.02); No AC −0.41 (−1.02, 0.19); AC present −0.28 (−0.95, 0.40); Stove electric −0.62 (−1.53, 0.30); gas −0.26 (−0.82, 0.31); No allergen exposure +0.30 (−0.76, 1.35); Exposed −0.39 (−0.92, 0.14) \| LBW/premature—Female −1.78 (−3.17, −0.40); Male −1.71 (−3.05, −0.37); Black −1.20 (−3.30, 0.89); Hispanic −1.67 (−3.47, 0.13); Atopy 0 −1.53 (−3.62, 0.57); 1–3 −2.32 (−3.77, −0.86); ≥4 −1.66 (−3.49, 0.16); No meds −3.19 (−5.93, −0.45); β-agonist/xanthines −0.73 (−2.14, 0.69); Cromolyn no steroids −0.03 (−3.46, 3.41); Steroids −2.69 (−4.16, −1.21); Crowding ≤1.0 −0.73 (−2.11, 0.66); >1.0 −2.54 (−3.93, −1.15); No AC −1.40 (−2.77, −0.03); AC present −2.12 (−3.51, −0.74); Stove electric −2.73 (−4.72, −0.74); gas −1.49 (−2.68, −0.30); No allergen exposure +0.34 (−2.13, 2.81); Exposed −2.00 (−3.14, −0.86) — Morning symptoms OR: Normal/term—Female 1.13 (0.93, 1.37); Male 1.10 (0.93, 1.30); Black 1.13 (0.87, 1.47); Hispanic 1.26 (1.03, 1.55); Atopy 0 1.29 (0.98, 1.71); 1–3 1.04 (0.85, 1.28); ≥4 1.08 (0.86, 1.34); No meds 0.87 (0.64, 1.20); β-agonist/xanthines 1.13 (0.92, 1.37); Cromolyn no steroids 1.67 (1.17, 2.39); Steroids 1.08 (0.86, 1.34); Crowding ≤1.0 1.14 (0.94, 1.38); >1.0 1.08 (0.92, 1.28); No AC 1.15 (0.98, 1.36); AC present 1.06 (0.88, 1.28); Stove electric 1.34 (1.02, 1.75); gas 1.04 (0.89, 1.21); No allergen exposure 1.06 (0.78, 1.45); Exposed 1.12 (0.97, 1.29) \| LBW/premature—Female 1.09 (0.71, 1.66); Male 1.45 (1.01, 2.08); Black 0.73 (0.34, 1.56); Hispanic 1.55 (0.73, 3.32); Atopy 0 1.47 (0.87, 2.50); 1–3 1.44 (0.89, 2.32); ≥4 0.96 (0.62, 1.47); No meds 3.06 (1.34, 6.95); β-agonist/xanthines 1.34 (0.87, 2.06); Cromolyn no steroids 1.02 (0.44, 2.37); Steroids 1.14 (0.72, 1.78); Crowding ≤1.0 1.34 (0.84, 2.14); >1.0 1.33 (0.97, 1.84); No AC 1.65 (1.05, 2.61); AC present 1.13 (0.80, 1.60); Stove electric 0.80 (0.40, 1.59); gas 1.44 (1.04, 1.98); No allergen exposure 1.85 (0.79, 4.36); Exposed 1.26 (0.92, 1.71).;↓ PEFR (with lags of 1-5day); ↑morning symptoms (lag of 1-4days); greater effects observed in children born > 3 week prematurely or weighing <5.5 lb. |
| Avol et al. (2001)^127^ | Southern California/1998 | Children (n=110) | Annual average level (10 ppb) | FEV1, FVC, MMEF, PEFR | Mean change: FVC −1.4 mL (−10.8, 8.0); FEV₁ +0.1 mL (−8.7, 8.9); MMEF −3.4 mL/s (−23.6, 16.8); PEFR −8.9 mL/s (−41.6, 23.8). |
| Mortimer et al. (2002)^128^ | 8 urban areas/1993 | Children (n=846) | Multiday moving average level (15 ppb) | PEFR | Morning—PEFR %Δ lag1 −0.06 (−0.38, 0.26), lag2 −0.13 (−0.40, 0.15), lag3 −0.32 (−0.59, −0.05), lag4 −0.22 (−0.48, 0.05), lag5 −0.22 (−0.49, 0.05), lag6 0.07 (−0.20, 0.33), avg lag1–5 −0.59 (−1.05, −0.13); ≥10% decline OR lag1 1.04 (0.97, 1.12), lag2 1.04 (0.98, 1.11), lag3 1.04 (0.97, 1.10), lag4 1.09 (1.02, 1.16), lag5 1.03 (0.97, 1.10), lag6 0.97 (0.91, 1.03), avg lag1–5 1.14 (1.02, 1.27); symptoms OR lag1 1.03 (0.94, 1.12), lag2 1.10 (1.02, 1.19), lag3 1.01 (0.94, 1.09), lag4 1.09 (1.01, 1.17), lag5 0.99 (0.92, 1.07), lag6 0.97 (0.90, 1.04), avg lag1–4 1.16 (1.02, 1.30); Evening—PEFR %Δ avg0–4 −0.05 (−0.51, 0.41); ≥10% decline OR avg0–4 1.00 (0.89, 1.13); symptoms OR avg0–3 1.02 (0.88, 1.18) |
| Delfino et al. (2003)^129^ | Los Angeles/1999-2000 | Children (n=22) | daily 1-h (14 ppb) or 8-hr average (10.8 ppb) with lags | PEF, asthma symptoms severity | 1-h max—lag0: OR>1 = 1.03 (0.72–1.48), OR>2 = 1.99 (1.06–3.72); lag1: OR>1 = 1.02 (0.70–1.50), OR>2 = 0.85 (0.56–1.28); 8-h max—lag0: OR>1 = 0.90 (0.60–1.36), OR>2 = 1.98 (1.03–3.80); lag1: OR>1 = 1.18 (0.79–1.75), OR>2 = 1.05 (0.73–1.52).; Lag 0 1-h and 8-h O3 was associated with symptom scores > 2 but not symptom scores > 1; no association between O3 and PEF was found |
| Lewis et al. (2005)^130^ | Detroit/1999-2002 | Children (n=86) | Daily average and 8-hour average levels with lag 1-5 (IQR: 14.5-16.0 ppb) | PF, FEV1 | % change in FEV₁ — CS users: daily mean—lag1 −0.41 (−3.02, 2.19; p=0.76), lag2 −0.73 (−3.21, 1.75; p=0.56), lag3–5 −1.86 (−4.86, 1.14; p=0.22); 8-hr peak—lag1 +1.75 (−0.20, 3.70; p=0.08), lag2 +3.19 (0.29, 6.08; p=0.03), lag3–5 −0.03 (−0.28, 0.22; p=0.82). URI present: daily mean—lag1 +4.08 (−1.78, 9.94; p=0.17), lag2 +7.62 (−0.49, 15.73; p=0.07), lag3–5 +1.47 (−7.73, 10.67; p=0.75); 8-hr peak—lag1 +5.79 (1.74, 9.85; p=0.005), lag2 +4.74 (0.46, 9.02; p=0.03), lag3–5 +0.27 (0.01, 0.53; p=0.04).↓FEV1 associated with 8-hr peal O_3_ exposure at lag1 and lag2 |
| Qian et al. (2005)^131^ | 4 U.S. communities/1990-1992 | Adults (n=10,240) | 1-year average level (SD: 8.3 ppb) | FVC, FEV1, FEV1/FVC | FVC: β=-0.0145 (p=0.0001), FEV1: β=-0.0107 (p=0.0001) with stronger effects among smokers or respiratory medication users or those who currently experience respiratory symptoms |
| Tager et al. (2005)^132^ | Berkeley/2000-2002 | Adults (n=255) | Lifetime average exposure (1 ppb) | FVC, PEER, FEV1, FEF75, FEF25–75 | FEV₁: Men β_O₃(eco) −0.011 (0.006), β_O₃(time-out) −0.002 (0.001); Women β_O₃(eco) −0.011 (0.004), β_O₃(time-out) −0.003 (0.0009) • LnFEF₇₅: Men β_O₃(eco) −0.027 (0.002), β_O₃(time-out) −0.006 (−11.75); Women β_O₃(eco) −0.029 (0.002), β_O₃(time-out) −0.006 (0.0005) • LnFEF₂₅–₇₅: Men β_O₃(eco) −0.020 (0.002), β_O₃(time-out) −0.004 (−12.48); Women β_O₃(eco) −0.23 (0.002), β_O₃(time-out) −0.005 (0.0004) • O₃×(FEF₂₅–₇₅/FVC) interaction: Men +0.007 (0.007) & +0.003 (0.001); Women +0.007 (0.003) & +0.002 (0.0008) |
| Girardot et al. (2006)^133^ | North Carolina and Tennessee (Great Smoky Mountains National Park)/2002, 2003 | Adult Hikers (n=354) | Daily 15 min-average level (1 ppbv) | FVC, FEV1, FVC/FEV1, PEF | Univariate: FVC 0.015±0.021 (p=0.484), FEV₁ 0.027±0.018 (p=0.145), PEF 0.089±0.057 (p=0.118), FVC/FEV₁ −0.017±0.026 (p=0.525), FEF₂₅–₇₅ −0.051±0.107 (p=0.634); Adjusted: FVC 0.007±0.024 (p=0.763), FEV₁ 0.024±0.020 (p=0.234), PEF 0.118±0.062 (p=0.059), FVC/FEV₁ −0.028±0.016 (p=0.074), FEF₂₅–₇₅ −0.041±0.064 (p=0.523); Piecewise (>40 ppbv): FVC −0.019±0.037 (p=0.613), FEV₁ −0.003±0.032 (p=0.911), PEF 0.127±0.098 (p=0.195), FVC/FEV₁ −0.025±0.025 (p=0.314), FEF₂₅–₇₅ −0.045±0.101 (p=0.659) |
| Alexeeff et al. (2007)^134^ | Greater Boston/1995-2005 | Male adults (n=904) | 48-h average level before visit (15 ppb) | FEV1, FVC | Overall: FEV₁ −1.29% (−1.95, −0.63); FVC −1.36% (−2.01, −0.70); AHR Nonresponder: FEV₁ −1.28% (−2.01, −0.54); FVC −1.29% (−1.96, −0.61); AHR Responder: FEV₁ −3.04% (−4.70, −1.34); FVC −2.03% (−3.57, −0.46); BMI <30: FEV₁ −1.15% (−1.91, −0.39); FVC −1.18% (−1.88, −0.48); BMI ≥30: FEV₁ −2.63% (−3.85, −1.39); FVC −2.05% (−3.17, −0.91); greater decrement in the obese than nonobese |
| Gauderman et al. (2007)^135^ | Southern California/1993-2004 | Children (n=3,677) | Annual average level | FVC, FEV1, MMEF | FVC (mL, difference vs >1500 m): <500 m [10y −17 (−70, 37); 18y −85 (−192, 22)]; 500–1000 m [10y −12 (−61, 37); 18y −54 (−151, 43)]; 1000–1500 m [10y −30 (−80, 21); 18y −81 (−181, 19)] FEV1 (mL, difference vs >1500 m): <500 m [10y −23 (−73, 28); 18y −121 (−219, −23)]; 500–1000 m [10y −32 (−78, 14); 18y −93 (−183, −4)]; 1000–1500 m [10y −34 (−81, 14); 18y −78 (−170, 14)] MMEF (mL/s, difference vs >1500 m): <500 m [10y −57 (−169, 56); 18y −230 (−432, −28)]; 500–1000 m [10y −92 (−195, 10); 18y −105 (−289, 79)]; 1000–1500 m [10y −45 (−150, 60); 18y −151 (−340, 38)] Percent predicted at 18 (<500 m): FEV1 97.0% (94.6–99.4); MMEF 93.4% (89.1–97.7); No association was observed between O3 exposure and reduced lung function |
| Islam et al. (2007)^136^ | Southern California/1993-2004 | Children (n=2,057) | 10-year annual average level (Not specified) | FEV1, FVC, FEF25-75 | No association between ozone exposure and lung function was found |
| Alexeeff et al. (2008)^137^ | Greater Boston/1995-2005 | Male adults (n=1,100) | Lag1-5 averages level before visit (15 ppb) | FEV1, FVC | % change in FVC: 1 day = -0.74 (-1.29, -0.17); 2 day = -1.29 (-1.95, -0.63); 3 day = -1.29 (-2.02, -0.55); 4 day = -1.02 (-1.81, -0.21); 5 day = -1.09 (-1.96, -0.21); % change in FEV1: 1 day = -0.83 (-1.43, -0.23); 2 day = -1.25 (-1.96, -0.54); 3 day = -1.08 (-1.86, -0.28); 4 day = -0.63 (-1.49, 0.23); 5 day = -0.66 (-1.61, 0.29); greater decrement among those carrying both the GSTP1 105Val variant and the HMOX1 long ^138^n repeat |
| Mortimer et al. (2008)^139^ | San Joaquin Valley/1989-2000 | Children (n=232) | Monthly average 8-hour maximums (lifetime and Prenatal, Trimester-specific, first 3 and 6 years) (Not specified) | FVC, FEV1, PEF, PEF25-75, FEV1/FVC, FEF25-75/FVC, FEF25, FEF75 | No association between ozone exposure and lung function was found |
| Thaller et al. (2008)^140^ | Galveston (Texas)/2002, 2003, 2004 | Adults (n=142) | Daily average and daily maximum level (10 ppm) | FVC, FEV1, FEV1/FVC | <Daily average> FVC: % Change=0.05 (-0.2, 0.3), FEV1/FVC: % change=-0.2 (-0.4, -0.04); <Daily Maximum> FVC: % change=0.06 (-0.07, 0.2), FEV1/FVC: % change=-0.1 (-0.2, -0.01) |
| Berhane et al. (2011)^141^ | 13 Southern Californian communities/2002-2003 | Children (n=2,240) | Cumulative daily average on lag days 1-23 days (IQR: 15.42 ppb) | FENO | % difference in FENO=14.25 (5.24, 24.04) |
| Eckel et al. (2012)^142^ | 4 U.S. counties/1990-1997 | Adults (n=3,382) | monthly 8-hour max average level (10 ppb) | FVC, FEV1 | FEV1—men current-month −5.3 mL (−23.2, 12.6), women +3.6 (−7.3, 14.5); men prior-month +7.5 (−25.7, 40.8), women +8.0 (−12.2, 28.3). FVC—men current-month −4.9 (−28.5, 18.7), women −0.3 (−16.5, 16.0); men prior-month −18.5 (−63.2, 26.2), women −3.3 (−34.2, 27.6). Typical ozone months (per +1 month): FEV1—men −2.4 (−3.3, −1.5), women −1.2 (−1.7, −0.7); FVC—men −8.7 (−9.8, −7.6), women −5.3 (−6.0, −4.6). |
| Rice et al. (2013)^143^ | Boston/1995-2001, 2002-2005, 2008-2011 | Framingham Offspring and Third Generation cohorts (n=3,262) | Categorical according to AQI and 1, 2, 3, 5, and 7-day moving averages before measurement (10 ppb) | FEV1, FVC | <Categorical: Good vs Moderate> Difference in FEV1=-55.7 mL (-100.7 mL, -10.8 mL), Difference in FVC=-50.6 mL (-13.6 mL, 2.4 mL); <Moving day average> FVC=Not significant for whole day's moving average, FEV1= Not significant except for 1- and 2-day moving average |
| Berhane et al. (2014)^144^ | Southern California/2006-2008 | Children (n=1,211) | daily 8-hour average during the test day and 60 days prior to testing (IQR: 2.1 ppb) | FeNO | Difference: 0.74 (-4.47, 3.01) |
| Urman et al. (2014)^145^ | Southern California/2002-2003 | Children (n=1,811) | 6-year average daily 8-hour max level (22.7 ppb) | FEV1, FVC | % difference: FEV1=-3.10% (-5.24%, -0.91%), FVC=-0.31% (-3.11%, 2.57%) |
| Neophytou et al. (2016)^146^ | U.S. and Puerto Rico/Not specified | Children (n=1449(Latino)+519(African American)) | 24-h average (Not specified) | FEV1, FVC, FEF25–75 | % change: <FEV1> Chicago=-1.33 (-7.53, 5.29), Houston=-0.14 (-5.79, 5.85), New York=-3.13 (-11.74, 6.32), Puerto Rico=1.54 (-2.37, 5.61), SF-GALA2=-0.59 (-3.10, 1.97), SF-SAGE2=-1.06 (-2.89, 0.81) |
| Schachter et al. (2016)^147^ | New York | Children (n=36) | Daily average levels with time lags up to 7 days (IQR: Not specified) | FEV1, Symptoms, albuterol puff use | <FEV1> Summer: OR=1.94 (1.02, 3.69), Winter: OR=0.73 (0.38, 1.43); <Symptoms> Summer: OR=1.95 (1.15, 3.30), Winter: OR=1.02 (0.65, 1.61), <Albuterol use> Summer: OR=1.69 (1.00, 2.88), Winter: OR=0.94 (0.57, 1.55) |
| Gilliland et al. (2017)^148^ | Southern California/1992-1993, 1995-1996, 2002-2003 | Children (n=2,120) | 4-year annual average level (Not specified) | FVC, FEV1 | No association was observed between ozone exposure and reduced lung function |
| Magzamen et al. (2018)^149^ | Seattle and Tacoma/2011-2013 | Adult with COPD (n=35) | Daily average 8-hour max level (IQR: 14.12 ppb) | daily count of short-acting beta-agonist inhaler use (puffs) | RR=0.989 (0.950, 1.030) |
| Aaron et al. (2019)^150^ | 6 U.S. cities/2000-2012 | Children (n=3,023) | 3-year average level (IQR: 3 ppb) | Total pulmonary vascular volume assessed on noncontrast computed tomography (TPVVCT) | No association between ozone exposure and difference in TPVVCT was found |
| Benka-Coker et al. (2019)^151^ | Fresno/2000-2008 | Children with asthma (n=315) | Daily 24-h average level (SD: 12.4 ppb) | FEV1, FVC, FEF25-75 | FEV1: β = −0.06 (−0.36, 0.25), FVC: β = −0.09 (−0.39, 0.22), FEF25-75%: β = 0.11 (−0.21, 0.42) |
| Benka-Coker et al. (2020)^152^ | Fresno/2000-2008 | Children (n=153) | 3 months average level (SD: 12.4 ppb) | FEV1, FVC, FEF25-75 | FEV1: β = −0.06 (−0.36, 0.25), FVC: β = −0.09 (−0.39, 0.22), FEF25-75%: β = 0.11 (−0.21, 0.42) |
| Martenies et al. (2020)^153^ | Colorado/Not specified | Adults (n=36) | Daily average level (1 doubling of ozone level: 100 ppb) | FVC, FEV1, FEF25-75, PEFR | FVC: % points=0.44 (-2.27, 3.15), FEV1: % points=0.80 (-2.43, 4.04), FEV/FVC: % points=0.04 (-2.15, 2.23), FEF25-75%: % points=4.43 (-4.76, 13.62), PEFR: % points=4.86 (-6.80, 16.53) |
| Marcus (2021)^154^ | California/2009-2017 | Children (n=5,788) | Daily 8-h max level (0.1 ppm) | Aerobic capacity (VO2max) | % of students with poor aerobic capacity: aerobic capacity (VO2max) =0.680 (p<0.05) can be affected even below NAAQS |
| Hao et al. (2022)^155^ | Los Angeles/2019 | Children with asthma (n=40) | Daily average of same day and lagged (up to 2 days) exposures (SD: 9.2 ppb) | FEV1, PEF, Asthma symptoms, inhaler use | FEV1 % predicted=-3.45% (-6.42%, -0.47%); inhaler use: RR=1.52 (1.02, 2.27); PEF: Not specified; Asthma symptoms: Not specified |
| Altman et al. (2023)^93^ | U.S. cities/2015-2017 (MUPPITS1 cohort), 2006-2009 (ICATA cohort) | Children with asthma/6–17/n=208+419 | AQI values matched to respiratory illness events (1 ppm) | FEV1, FEV1/FVC | FEV1 % predicted: β = –0.35, FDR = 0.014; FEV1/FVC: β = –0.0028, FDR = 0.014 |
| Chen et al. (2023)^156^ | Boston/2017-2019 | Adults (former smoker with moderate-to-severe COPD) (n=30) | Prior-daily personal exposure average (IQR: 5.1 ppb) | FEV1, FVC | Not find evidence of EMM by ozone on pulmonary function |
| Neophytou et al. (2023)^157^ | Fresno-Clovis (Central California)/2015-2017 | Children (n=222) | Monthly average during prenatal months and first three years of life (12.42 ppb) | FVC, FEV1, FEV1/FVC | No association was found between O_3_ exposure and decrements in lung function |
| Hazlehurst et al. (2024)^158^ | Tennessee/2006-2011 | Children (n=648) | Lifetime average from birth to age 8 (2 ppb) | FEV1, FVC, FEF25-75% | Difference in lung function: <FEV1> 0.14 (-0.03, 0.30), <FVC> 0.05 (-0.11, 0.21), <FEF 25-75%> 0.17 (0.00, 0.34) |
| Dearborn et al. (2025)^159^ | Tennessee/ 2006-2011 | Children (n=661) | average during pseudo glandular (5–16 weeks), canalicular (16–24 weeks), and saccular (24–35 weeks), per 10ppb | lung function at 8-9 years: FEV1, FVC, FEV1/FVC, and FEF25–75 | saccular window: FEV1/FVC: −0.23 (95% CI: −0.52-0.05) and FEF25–75: −0.17 (95% CI: −0.43-0.09); overall pregnancy: FEV1: −0.28 (95% CI: −0.63-0.08), FVC: −0.13 (95% CI: −0.50-0.23), FEF25–75: −0.37 (95% CI: −0.75-0.01), and FEV1/FVC: −0.30 (95% CI: −0.72-0.12) |
| Sack et al. (2025)^160^ | USA/2014-2018 | Adults (n=835) | 5-year average before the baseline (3ppb) | FVC, %DLCO, Supplemental Oxygen Use, respiratory-specific health-related quality of life | At Enrollment: <FVC> 1.57 (95%CI: 0.15, 2.98), <%DLCO>: 1.09 (95%CI: -0.09-2.26), supplemental Oxygen Use: OR=1.07 (95%CI: 0.86-1.35),St. Georges total: 0.38 (95%CI: -1.17, 1.93), CASA-Qcough impact: -0.42 (95%CI: -2.14-1.30), CASA-Qcough symptom: -0.64 (95%CI: -2.33-1.06),EuroQoL score: 0.72 (95%CI: -1.00-2.45), EuroQoL visual analog scale: 0.39 (95%CI: -1.18-1.96),SF-12 mental Component: 0.01 (95%CI: -0.74-0.76), SF-12 mental Component: -0.08 (95%CI: -0.91-0.74);1-year follow-up:None association was found |
| Xue et al. (2025)^161^ | Northeastern U.S./2000-2021 | Adults with fibrotic interstitial lung disease (n=1,424; FVC analysis n=1,106, DLCO analysis n=1,030) | 1-year average level (1ppb) | Baseline FVC % predicted, DLCO % predicted | <FVC % predicted> NN β=-0.01 (95% CI: -0.47–0.44), IDW β=0.81 (95% CI: 0.20–1.43), Kriging β=1.03 (95% CI: 0.36–1.72); <DLCO % predicted> NN β=-0.06 (95% CI: -0.22–-0.006), IDW β=0.001 (95% CI: -0.13–0.13), Kriging β=0.04 (95% CI: -0.11–0.18). |

| **Table S2. Studies of Ozone Exposure and Asthma-related symptoms, ED visits and Hospitalizations (Not Included in the Main Text)** | | | | | |
| --- | --- | --- | --- | --- | --- |
| **Author (year)** | **Study region/year** | **Study population** | **Exposure metrix (per unit increase)** | **Health Outcome** | **Effect Estimate** |
| **Asthma-related symptoms** | | | | | |
| Perry et al. (1983)^162^ | Denver/1979 | Asthma patients (n=24) | 12-hour average level (Not specified) | Asthma symptom score, nebulizer usage | Asthma symptom score: p=0.6303 (AM, PM); Nebulizer usage: p=0.5809 (AM), 0.6625 (PM) |
| Greer et al. (1993)^163^ | U.S./1977, 1987 | Adults (Seventh Day Adventists) (n=3,914) | Annual mean level over 20 years (1 pphm) | Incidence of Asthma | Overall: RR=1.31 (0.96, 1.78), Men: RR=3.12 (1.61, 5.85), Women: RR=0.94 (0.65, 1.34) |
| Abbey et al. (1995)^164^ | California/1977-1987 | Adults (n=3,914) | Annual mean level (1 pphm) | Incidence of Asthma | Overall: RR=1.35 (0.93, 1.96), Men: RR=3.12 (1.61, 5.85), Women: RR=0.94 (0.61, 1.45) |
| Avol et al. (1998)^165^ | Southern California/1996 | Children (n=195; asthmatic 49, wheezy 53, healthy 93) | 24-hour average level (Low ozone: less than 15.6 ppb, High ozone: greater than 32.4 ppb) | Asthmatic group: Trouble breathing, Wheezing, Oral medication, Inhaler use | Trouble breathing_High: OR=5.35 (3.13, 9.15), Trouble breathing_Low: OR=5.06 (2.83, 9.06), Wheezing_High: OR=6.14 (3.72, 10.13), Wheezing_Low: OR=3.68 (2.13, 6.35), Oral medication_High: OR=2.03 (0.99, 4.17), Oral medication_Low: OR=1.98 (0.97, 4.04), Inhaler use_High=7.74 (3.14, 19.04), Inhaler use_Low=6.27 (2.67, 14.71) |
| Delfino et al. (1998)^166^ | Southern California/1995 | Children with asthma (n=25) | Daily 8-hour maximum level (58 ppb) | Adverse asthma symptoms | OR=2.15 (1.04, 4.44) |
| Ostro et al. (2001)^167^ | Los Angeles/1993 | Children (n=83) | Daily average level (0.4 ppm) | Asthma symptoms, medication use | Medication use: OR=1.15 (1.12, 1.19) |
| Mortimer et al. (2002)^128^ | 8 U.S. cities/1993 | Children with asthma (n=846) | Daily average level (15 ppb) | Asthma symptoms | OR=1.16 (1.02, 1.30) |
| Delfino et al. (2003)^129^ | Los Angeles/1999-2000 | Children (Hispanic, n=22) | Daily 1-hr max level (IQR: 14 ppb), daily 8-hr max level (IQR: 10.8 ppb) | Asthma symptom score | Symptom score >1 and 1-hr max: OR=1.03 (Lag 0: 0.72, 1.48), OR=1.02 (Lag 1: 0.70, 1.50); Symptom score >2 and 1-hr max: OR=1.99 (Lag 0: 1.06, 3.72), OR=0.85 (Lag 1: 0.56, 1.28); Symptom score >1 and 8-hr max: OR=0.90, (Lag 0: 0.60, 1.36), OR= 1.18 (Lag 1: 0.79, 1.75); Symptom score >2 and 8-hr max: OR=1.98 (Lag 0: 1.03, 3.80), OR=1.05 (Lag 1: 0.73, 1.52) |
| Millstein et al. (2004)^168^ | Southern California/1994-1995 | Children (n=2,034) | Annual average level (IQR: 27.83 ppb) | Annual asthma medication use and wheeze | Medication use: OR=1.80 (1.19, 2.70), Wheeze: OR=0.84 (0.62, 1.14) |
| Rabinovitch et al. (2004)^169^ | Denver/1999-2002 | Children with asthma (n=147) | 3-day moving average level (11.4 ppb) | Asthma exacerbations | OR=0.91 (0.785, 1.056) |
| Schildcrout et al. (2006)^170^ | 8 U.S. cities/1993-1995 | Children with asthma (n=990) | Daily 1-hour maximum level (30 ppb) | Asthma symptoms, rescue inhaler use | Asthma symptoms: OR=1.01 (3-day moving sum: 0.94, 1.09), 1.06 (Lag 0: 0.92, 1.23), 1.00 (Lag 1: 0.88, 1.14), 1.02 (Lag 2: 0.92, 1.13); Inhaler use: RR=1.00 (3-day moving sum: 0.95, 1.04), 1.01 (Lag 0: 0.92, 1.10), 0.99 (Lag 1: 0.92, 1.06), 1.00 (Lag 2: 0.95, 1.06) |
| O’Connor et al. (2008)^171^ | 7 U.S. urban communities/1998-2001 | Children with persistent asthma (n=861) | Daily average level (26.7ppb) | Asthma symptoms | OR=1.03 (0.82, 1.28) |
| Mann et al. (2010)^172^ | Fresno/2000-2005 | Children with asthma (n=315) | Daily maximum 8-hour level (20 ppb) | Wheeze in asthmatic children | OR=1.01 (0.92, 1.12) |
| McConnell et al. (2010)^173^ | Southern California/2002-2003 | Children (wheeze/asthma-free at baseline, n=2,497) | Daily 8-hour average level (30.3 ppb) | New-onset asthma | HR=0.76 (0.38, 1.54) |
| Nishimura et al. (2013)^174^ | Chicago, Bronx, Houston, San Francisco/2006-2011 | Children and adolescents (n=4,320) | Annual average 8-hour level (5 ppb) | New onset asthma risk | Huston: OR=1.01 (0.59, 1.71), Puerto Rico: 1.86 (0.94, 3.98), Chicago: OR=0.94 (0.59, 1.50), New York: OR=0.83 (0.48, 1.42) |
| Li and Lin (2014)^175^ | U.S./2006–2009 | Adults (n=412,832) | 4-year average of the annual fourth-highest daily maximum ozone concentration (1 ppb) | Asthma risk | OR=1.023 (1.010, 1.027) |
| Wendt et al. (2014)^176^ | Harris/2005-2007 | Children (Medicaid-enrolled) (n=18,289) | daily maximum 8-h moving average (10 ppb) | New-onset asthma diagnosis | OR=1.05 (1.02, 1.08) |
| Brewer et al. (2017)^177^ | Houston/2010-2012 | Children's medical records (n=142,407) | Average of quarterly means over three years (Based on daily 8-hour maximum level) (Range: 23.8-31.4 ppb) | Childhood asthma diagnosis risk relative to whites | Model 3: OR=1.01 (0.97, 1.06), Model 5: OR=1.02 (0.97, 1.06), Model 6: OR=1.03 (0.99, 1.08) |
| Cox (2017)^178^ | U.S./2008–2012 | Adults (n=228,369) | Daily average level (Not specified) | Asthma risk | OR=1.66 (0.26, 10.6) |
| Li et al. (2019)^179^ | Florida/2010-2012 | Dyads of pediatric patients with asthma and parents (n=229) | Mean concentration within a 7-day (IQR: 12.3) | Asthma control status | Model 2: β=-0.02 (-0.15, 0.12), Model 4=-0.16 (-0.34, 0.02) |
| Huang et al. (2021)^180^ | Philadelphia metropolitan/2011–2014 | Pediatric asthma exacerbation cases (n=54,632) | Daily 8-hour max level during warm and cold months (IQR_Warm: 15.19 ppb, IQR_Cold: 12.33 ppb) | Asthma exacerbation | Warm Season: OR= 1.00 (0.95-1.06), Cold season: OR=1.08 (1.02, 1.14) |
| Hao et al. (2022)^155^ | Los Angeles/2019 | Children with asthma (n=40) | Daily average of same day (9.2 ppb) | Asthma symptoms, inhaler use | Inhaler use: RR=1.52/9.2ppb, Asthma symptoms: OR=0.96 (0.61, 1.52) |
| Hurbain et al. (2022)^181^ | U.S. counties/2003–2012 | General population (n=705,632; Asthma=91,705) | Annual level of Air Quality Index (AQI quartiles used but specific cut-offs not reported) | Asthma prevalence in U.S. counties | Q2: OR=0.93 (0.82, 1.05), Q3: OR=0.93 (0.83, 1.05), Q4: OR=0.88 (0.78, 0.98) |
| Su et al. (2022)^182^ | Kentucky/2012-2017 | General population (n=1,039) | Daily maximum 8-hr average (IQR: 15.65 ppb) | Asthma rescue medication usage | +0.113 mean inhaler uses per day (0.097, 0.129) |
| Mirabelli et al. (2023)^183^ | North Carolina/2001–2016 | Children (n=80) | Daily ozone level (lagged by 3-5 days, tertile range: 43.99-92.4 ppb) | Fatal asthma exacerbations | OR=1.6 (0.6, 4.8) |
| Robles et al. (2023)^184^ | California/2003-2004 | Adolescents with asthma (n=476, CHIS 2003) | Annual average of daily 8-hour maximum level (10 ppb) | Asthma symptom frequency | [Adjusted] ≤3mi: β = –0.23 (-0.46, -0.002) , p = 0.048; ≤5mi: β = –0.12 (-0.29, 0.06), p = 0.20 |
| Hazlehurst et al. (2024)^185^ | San Francisco, Minneapolis, Rochester, Seattle/2006-2011 | Mother-child dyads (n=1,279) | Average level during the pregnancy and after birth to 4 years (5 ppb) | Current Asthma at age 8-9, Asthma with recent exacerbation | Current Asthma: RR_5-16 week=1.05 (0.90, 1.23), RR_16-24 week=1.00 (0.85, 1.16), RR_24-36 week=0.96 (0.80, 1.14), RR_36+week=0.94 (0.82, 1.08); Recent exacerbation: RR_5-16 week=1.20 (0.95, 1.51), RR_16-24 week=0.97 (0.79, 1.20), RR_24-36 week=0.96 (0.77, 1.20), RR_36+week=0.96 (0.78, 1.17) |
| Kelchtermans et al. (2024)^186^ | Philadelphia/Not specified | Pediatric patients with asthma (EMR data) (n=8,129) | Daily county level (exacerbation associated with ambient air pollution for ozone at least 2 SD above the daily average) | Severity of pediatric asthma | OR=1.39 (1.08, 1.78) |
| Amialchuk et al. (2025)^187^ | U.S. Add Health Waves IV (2008) and V (2016–2018) data; O₃ exposure data (2002–2017) | Children (n=6,860; 3,462 males and 3,398 females) | Residential census tract–level long-term average level (1ppb) | Physician diagnosis of asthma | <Asthma, all children> β=0.002 (SE=0.002); <Asthma, males> β=0.003 (SE=0.003); <Asthma, females> β=0.001 (SE=0.003); <Asthma, females aged 10–12 years at Wave V> β=0.011 (p<0.05) |
| Byrwa-Hill et al. (2025)^188^ | Mon Valley/2018-2020 | EMR data (n=117,545 residents with 675 asthma, 423 bronchitis, and 696 COPD exacerbation visits) | Same day 8-hour AQI (>100 vs. ≤100) | Asthma exacerbations | <Overall asthma exacerbations> RR=1.73 (95% CI: 0.56–5.37); <Age <18 years> RR=1.50 (95% CI: 0.21–10.76); <Age <18 years, female> RR=3.40 (95% CI: 0.50–24.79); <Age <18 years, public insurance> RR=1.80 (95% CI: 0.25–12.94); <Age 18–64 years> RR=0.52 (95% CI: 0.13–2.10); <Age 18–64 years, female> RR=0.81 (95% CI: 0.20–3.26); <Age 18–64 years, public insurance> RR=0.93 (95% CI: 0.23–3.72) |
| Dearborn et al. (2025)^189^ | 6 U.S. cities (Memphis, TN; Seattle and Yakima, WA; Minneapolis, MN; Rochester, NY; and San Francisco, CA)/2007-2023 | Full-term children (n=1,188) | Average level from birth to age 2 years (2ppb) | Caregiver-reported current asthma and current wheeze at ages 4–6 years; strict asthma at ages 8–9 years; and wheeze trajectories | <Current asthma at ages 4–6 years> OR=1.31 (95% CI: 1.02–1.68); <Current wheeze at ages 4–6 years> OR=1.30 (95% CI: 1.05–1.64); <Strict asthma at ages 8–9 years> OR=1.06 (95% CI: 0.77–1.45); <Early wheeze vs. never wheeze> OR=1.00 (95% CI: 0.89–1.43); <Late wheeze vs. never wheeze> OR=0.96 (95% CI: 0.86–1.57); <Persistent wheeze vs. never wheeze> OR=1.16 (95% CI: 0.84–1.73). |
| Lu et al. (2025)^190^ | Fresno and Clovis/2015-2019 | Children (n=299) | 12-month average ambient O₃level before each visit (IQR=0.72ppb) | Persistent cough and wheeze in the past 12 months among children with a history of asthma diagnosis | <Wheeze in the past 12 months> single-pollutant OR=1.16 (95% CI: 0.91–1.48), single-pollutant + IPW OR=1.13 (95% CI: 0.87–1.45), PM₂.₅-adjusted OR=1.20 (95% CI: 0.93–1.55), PM₂.₅-adjusted + IPW OR=1.16 (95% CI: 0.88–1.53); <Cough for more than 2 days in the past 12 months> single-pollutant OR=1.39 (95% CI: 1.08–1.78), single-pollutant + IPW OR=1.39 (95% CI: 1.06–1.82), PM₂.₅-adjusted OR=1.40 (95% CI: 1.08–1.81), PM₂.₅-adjusted + IPW OR=1.40 (95% CI: 1.05–1.86). |
| Po et al. (2025)^191^ | 9 communities in Southern California/1993-2014 | Children (n=1,794 children with 260 incident asthma cases) | Baseline annual average level (IQR=10.1ppb) | New physician-diagnosed childhood asthma, | <Incident childhood asthma> overall aRR=1.01 (95% CI: 0.75–1.35), Hispanic aRR=1.03 (95% CI: 0.64–1.69), non-Hispanic aRR=1.14 (95% CI: 0.78–1.67); <O₃ × PRS interaction> overall β=-0.09 (95% CI: -0.24–0.08), Hispanic β=-0.32 (95% CI: -0.57–-0.07), non-Hispanic β=-0.01 (95% CI: -0.24–0.21). |
| Wang et al. (2025)^192^ | 32 ECHO sites across the contiguous U.S./1981-2021 (with modeled ozone data available for 2000-2016) | Children (n=17,657; 2,743 incident asthma diagnoses by age 10) | Annual 8-hour maximum O₃ level (IQR=6.87ppb) | Incident physician-diagnosed asthma | <Prior-year exposure, main model> HR=1.11 (95% CI: 1.01–1.22); <Prior-year exposure, three-pollutant model> HR=1.05 (95% CI: 0.95–1.16); <Cumulative exposure from pregnancy through the prior year> HR=0.86 (95% CI: 0.69–1.06); <Prior-year exposure, age 5> HR=1.22 (95% CI: 1.08–1.39); <Prior-year exposure, age 10> HR=1.00 (95% CI: 0.81–1.23). |
| Wang et al. (2026)^193^ | U.S.A./1997-2014 | Women in the Nurses' Health Study II (n=4,326) | 48-month average ambient O₃ level (10ppb) | Asthma exacerbation requiring hospitalization, an emergency department visit, or an urgent care visit in the past year | <Asthma exacerbation> single-pollutant model OR=0.85 (95% CI: 0.70–1.04); multi-pollutant model OR=1.07 (95% CI: 0.85–1.36). |
| **Asthma-related ED visits and Hospitalizations** | | | | | |
| Cody et al. (1992)^194^ | New Jersey/1988-1989 | ED visits data (n=~147,000) | 10 am-15 pm average level (Not specified) | Hospital visits for asthma | All year: β = 20.3 (Lag 1: p=0.005); 1988: β = 24.6 (Lag 1: p=0.0041); 1989: β = 27.7 (Lag 1: p=0.0206), β = 37.2 (Lag 0: p=0.0067) |
| White et al. (1994)^195^ | Atlanta/1990 | Children's ED visits data (n=600 visits) | 1-hr maximum level (Q1: <0.080 ppm, Q2: 0.080-0.089 ppm, Q3: 0.090-0.099 ppm, Q4: 0.100-0.109 ppm, Q5: ≥0.110 ppm) | Asthma ED visits | Q2: RR=1.01 (0.75, 1.36), Q3: RR=1.24 (0.93, 1.65), Q4: RR=1.29 (0.86, 1.93), Q5: RR=1.50 (1.02, 2.21) |
| Weisel et al. (1995)^196^ | Central New Jersey/1986-1990 | ED visits data (Not specified, Daily average: 4-6 visits) | Low vs High ozone level (Cutoff: 0.06 ppm) | Asthma ED visits | Daily mean ED visits (Low vs High): 4.08 vs 5.40 (1986, p=0.0697), 4.07 vs 5.20 (1987, p=0.0243), 5.83 vs 4.20 (1988, p=0.0216), 5.14 vs 7.07 (1989, p=0.0226), 5.69 bs 6.99 (1990, p=0.4082), 6.40 vs 5.27 (All years, p=0.0001) |
| Carlin and Xia (1999)^197^ | Atlanta/1993-1995 | 7 emergency care centers data (Not specified) | Daily maximum 8-hr average level (20 ppb) | Pediatric ER visits for asthma | RR=1.026 (1.000, 1.049) |
| Cassino et al. (1999)^198^ | New York City/1992-1995 | Adults with asthma (n=1,216) | Daily maximum 8-hr average level (50 ppb) | Asthma ED visits | RR=1.72 (1.13, 2.62) |
| Sheppard et al. (1999)^199^ | Seattle/1987-1994 | Hospital admissions data (n=7,837) | Daily maximum 8-hr average (20 ppb) | Asthma hospital admissions | Lag 0: RR=1.01 (0.97, 1.06), Lag 1: RR=1.03 (0.99, 1.08), Lag 2: RR=1.06 (1.02, 1.11), Lag 3: RR=1.02 (0.97, 1.07) |
| Tolbert et al. (2000)^200^ | Atlanta/1993-1995 | ED visits data (n=130,000 visits) | Daily maximum 8-hr average (20 ppb) | Asthma ER visits | RR=1.04 (p<0.05) |
| Tolbert et al. (2000)^201^ | Atlanta/1998-2000 | Asthma ED visits (n=1,068,810) | Daily maximum 8-hr average (25 ppb) | Daily asthma ED visits | RR=1.064 (p=0.03) |
| Friedman (2001)^202^ | Atlanta/1996 | Children's ED visits and hospital admission data from 4 databases (Not specified) | Daily maximum 8-hr average (50 ppb) | Asthma acute care visits during Olympic period | Georgia Medicaid: RR=0.48(0.44, 0.86), Health maintenance organization: RR=0.58 (0.32, 1.06), 2 pediatric emergency departments: RR=0.93 (0.46, 1.11), Georgia hospital discharge database: RR=0.71 (0.46, 1.11) |
| Weisel et al. (2002)^203^ | New Jersey/1995 | ER visits data (Not specified) | Daily 1-hr max, 5-hr average, and 8-hr average level (1 ppb) | Daily asthma ED visits and hospital admissions | <Same day ER visits> 1-hr max: β = 0.040 (p<0.05), 5-hr mean β = 0.039 (p<0.05), 8-hr mean β = 0.039 (p<0.05); <Hospital admissions> Lag 24-hr: β = 0.092 (p<0.05), Lag 48-hr: β = 0.110 (p<0.05), Lag 72-hr: Not specified (insignificant) |
| Jaffe et al. (2003)^204^ | Cincinnati, Cleveland, Columbus/1991-1996 | Children and adults (n=5,416) | Daily maximum 8-hr average (0.01 ppm) | Asthma ED visits | ED visits=+3% (0%, 6%) |
| Peel et al. (2005)^205^ | Atlanta/1993-2000 | ED visits data (n=30,000-40,000 visits/year) | 3-day moving averages (25 ppb) | Asthma ED visits | RR=1.022 (0.996, 1.049) |
| Wilson et al. (2005)^206^ | Portland/1998-2000, Manchester/1996-2000 | ER database (Not specified) | Daily maximum 8-hr average (IQR_Portland: 12.3ppbv, IQR_Manchester: 13.6 ppbv) | Asthma ER visits | Portland: RR=1.056 (1.010, 1.104), Manchester: RR=1.044 (0.004, 0.085) |
| Tolbert et al. (2007)^207^ | Atlanta/1993-2004 | ED visits and hospitalization data (n=10,206,389) | Daily maximum 8-hr average (29.75 ppb) | Emergency department visits for respiratory and cardiovascular outcomes | RR=1.039 (1.027, 1.052) |
| Babin et al. (2008)^208^ | Washington, DC/1994-2005 | Medicaid patients’ data (Not specified) | Daily 8-hr maximum level (0.01 ppm) | Daily Asthma ED visits | Mean change of ED visits=+4.6% (0.9%, 8.4%) |
| Babin et al. (2008)^209^ | Washington, DC/1994-2005 | Medicaid patients’ data (n=61,218) | Daily maximum 8-hr average (0.01 ppm) | General acute care visits for asthma | Mean change of ED visits=0.9% (0.2%, 1.7%) |
| Lin et al. (2008)^210^ | New York State/1995-2000 | Integrated Child Health Information System birth data (n=1,204,396) | Daily maximum 8-hr average (1 ppb) | Asthma hospital admissions | OR=1.16 (1.15, 1.17) |
| Moore et al. (2008)^211^ | California/1983-2000 | California Healthcare Information Resource Center data (n=13,209,192) | Daily 1-hr maximum level (10 ppb) | Asthma hospitalizations | RR=4.6% (p<0.05) |
| Paulu et al. (2008)^212^ | Maine/2000-2003 | Asthma ED visits data (n=8,020 visits) | Daily maximum 8-hr average (10 ppb) | Asthma ED visits | ED visits=+7% (4%, 11%) |
| Neidell and Kinney (2010)^213^ | Southern California/1989-1997 | California Hospital Discharge Data (n=115,446 counts) | 1-hour level (0.01 ppm) | Asthma hospitalizations | All ages: RR=1.00027 (1.00021, 1.00034), 5-19 years: RR=1.00037 (1.00024, 1.00051), 20-64 years: 1.00022 (1.00013, 1.00032), 65+ years: RR=1.00031 (1.00016, 1.00046) |
| Peel et al. (2010)^214^ | Atlanta/1996 | ED visits data (n=17,602 visits) | Daily 1-hr maximum level (During Olympic vs Reference) | Asthma ED visit | RR=0.953 (0.650, 1.399) |
| Silverman and Ito (2010)^215^ | New York/1999-2006 | Hospital data (n=75,383) | Daily maximum 8-hr average (22 ppb) | Asthma hospital admissions | ICU Admissions: RR=1.19 (1.01, 1.40), General Hospitalizations: RR=1.20 (1.11, 1.29) |
| Sinclair et al. (2010)^216^ | Atlanta/1998-2002 | Medicare ED visits data (n=47,572 visits) | Daily maximum 8-hr average level (1 SD: 24.01 ppb) | Asthma ED visits | <Children> 25 months lag0–2: RR=0.992 (95%CI 0.952–1.033), 25 months lag3–5: RR=0.976 (95%CI 0.937–1.017), 25 months lag6–8: RR=0.972 (95%CI 0.933–1.012), 28 months lag0–2: RR=1.002 (95%CI 0.969–1.036), 28 months lag3–5: RR=0.991 (95%CI 0.957–1.026), 28 months lag6–8: RR=0.986 (95%CI 0.952–1.021); <Adult> 25 months lag0–2: RR=1.016 (95%CI 0.962–1.072), 25 months lag3–5: RR=0.978 (95%CI 0.925–1.035), 25 months lag6–8: RR=0.981 (95%CI 0.927–1.038), 28 months lag0–2: RR=0.996 (95%CI 0.956–1.038), 28 months lag3–5: RR=0.978 (95%CI 0.937–1.020), 28 months lag6–8: RR=0.980 (95%CI 0.939–1.022) |
| Strickland et al. (2010)^217^ | Atlanta/1993-2004 | Children (n=91,386 visits) | Daily maximum 8-hr average (29.2 ppb) | Asthma ED visits | RR=1.062 (1.031, 1.093) |
| Hanna et al. (2011)^218^ | North Carolina/1996-2004 | North Carolina hospital data (Not specified) | Daily 1-hour maximum level (10 ppb) | Asthma hospitalizations | % change: DT=2%~5% (-0.05%, 8%, Current+lag 1-5), MT+/++=03% (0.2%, 6%, Current), TR=3%~4% (1%, 6%, Lat3-4) |
| Strickland et al. (2011)^219^ | Atlanta/1993-2004 | Children (n=41,741 visits) | Daily maximum 8-hr average (25 ppb) | Asthma ED visits | RR=1.070 (1.037, 1.104) |
| Delamater et al. (2012)^220^ | California/2001-2008 | Hospitalization data (Not specified) | Monthly means of daily concentrations | Monthly asthma hospitalizations | No associations |
| Glad et al. (2012)^221^ | Pittsburgh/2002-2005 | General population (n=6,979) | 1-hour maximum ozone level (10 ppb) | Asthma ED visits | OR=1.025 (p<0.05) |
| Winquist et al. (2012)^222^ | St. Louis/2001-2007 | ED visits and hospitalization data (Not specified) | Daily maximum 8-hr average level (IQR:27.3 ppb) | Asthma/wheeze related ED visits, hospital admissions | Non-elective Hospital admission: 1.101 (1.017, 1.192), ED visits: RR=1.069 (1.028, 1.111), Hospital admissions through ED visits: RR=1.106 (1.020, 1.200), All Hospital admission: RR=1.070 (0.992, 1.154) |
| Anderko et al. (2014)^223^ | Washington (District of Columbia)/2007-2010 | Hospitalization data (n=5,921) | Daily maximum 8-hr average (0.01 ppm) | Daily asthma hospitalization | Multiplicative effect estimate=0.988 (0.966, 0.010) |
| Delfino et al. (2014)^224^ | Orange County/2000-2008 | Infants and children (n=11,390 visits) | 24-hour daily average, 6-month averaged seasonal level | Asthma-related hospital encounters during warm and cool seasons | Not specified |
| Gass et al. (2014)^225^ | Atlanta/1999-2009 | Asthma ED visit data (Not specified, 4,010 days ED visits data) | 3-day moving average population-weighted level (Not specified) | Daily pediatric asthma ED visits | <Terminal node data> T1: RR=1.10 (1.05, 1.16); T2: RR=1.08 (1.03, 1.14), T7: RR=1.08 (1.01, 1.15); Other terminal nodes results were not statistically significant |
| Raun et al. (2014)^226^ | Houston/2004-2011 | Pediatric asthma patients (n=11,754) | Daily maximum 8-hr average (20 ppb) | Daily emergency ambulance-treated asthma attacks | RR=1.05 (1.00, 1.09) |
| Sacks et al. (2014)^227^ | North Carolina/2006-2008 | General population (n=121,621) | Daily maximum 8-hr average (20 ppb) | Daily asthma ED visits | OR=1.019 (0.998, 1.040) |
| Strickland et al. (2014)^228^ | Atlanta/2002-2010 | Children (n=109,758) | Daily maximum 8-hr average level (IQR: 24.03 ppb) | Daily ED visits for asthma or wheeze | RR=1.082 (1.051, 1.114) |
| Winquist et al. (2014)^229^ | Atlanta/1998-2004 | Pediatric asthma patients (Not specified) | 3-day moving average level (IQR: 29.18 ppb) | Daily counts of pediatric asthma ED visits during the warm season | Joint effect (ozone, NO₂, SO₂, CO, PM) RR=1.13 (1.06, 1.21) |
| Gass et al. (2015)^230^ | Atlanta/1999-2009, Dallas/2006-2009, St. Louis/2001-2007 | Children asthma patients (n=488,408) | Daily maximum 8-hr average level (Not specified) | Pediatric asthma ED visits | <Air polluntants mixture_O3/NO2/PM2.5> Mixture 10 (3/1/4, 3/2/4): RR=1.07 (1.03, 1.12); Mixture 13 (3/3/2, 3/3/3, 3/3/4, 3/4/2, 3/4/3, 3/4/4): RR=1.06 (1.02, 1.09); <Reference mixture_O3/NO2/PM2.5> 1/1/1 |
| Gleason et al. (2015)^231^ | Newark/2004-2007 | Children with asthma (n=case-cross over 3197+ Time-series 3675) | Daily maximum 8-hr average level (IQR_Same day: 16.95 ppb, IQR_Lag 1: 16.69 ppb, IQR_Lag 2: 16.52 ppb, IQR_Lag 3: 16.60 ppb, IQR_Lag 4: 16.73 ppb, IQR_Lag 5: 16.70 ppb, IQR_3-Day average: 12.80 ppb, IQR_5-Day average: 11.03 ppb) | Daily count of asthma ED visits | <Time-series_RR> Same-day: 1.08 (1.04–1.12), Lag 1: 1.08 (1.04–1.13), Lag 2: 1.03 (0.97–1.05), Lag 3: 1.06 (1.01–1.09), Lag 4: 0.98 (0.95–1.02), Lag 5: 0.90 (0.87–0.93), 3-Day average: 1.10 (1.06–1.15), 5-Day average: 1.10 (1.05–1.15); <Case-crossover_OR> Same-day: 1.10 (1.06–1.14), Lag 1: 1.08 (1.04–1.12), Lag 2: 0.99 (0.95–1.02), Lag 3: 1.01 (0.98–1.05), Lag 4: 0.94 (0.91–0.97), Lag 5: 0.88 (0.85–0.91), 3-Day average: 1.08 (1.04–1.12), 5-Day average: 1.03 (0.99–1.07) |
| Pearce et al. (2015)^232^ | Atlanta/1999-2008 | Children with asthma ED visits data (Not specified) | Daily maximum 8-hr average level (IQR: Not specified) | Pediatric asthma ED visits | RR=1.02 (1.01,1.03), Exact values were not reported and were instead estimated from the figure |
| Rodopoulou et al. (2015)^233^ | Central Arkansas/2002-2012 | Adults ED visits data (n=29,402) | Daily maximum 8-hr average level (10 ppbv) | Asthma ED visits | All period: % increase=2.78 (-4.89, 11.06), Cold period: % increase=2.95 (-10.88, 18.92), Warm period: % increase=0.44 (-8.79, 10.59) |
| Sheffield et al. (2015)^234^ | New York/2005-2011 | Children's ED visits and hospitalization data (n=8,009 (Hospitalization)+ 35,907 (ED visits)) | Daily 24-h average level (IQR: 0.013 ppm) | Asthma ED visits and hospitalizations | <ED visits> Boys: % excess risk= 2.9 (0, 5.9) – 8.4 (5.4, 11.5), Girls: % excess risk=5.4 (2, 9) – 6.5 (3.1, 10) |
| Malig et al. (2016)^235^ | California/2005-2008 | ED visits data (n=3.7 million) | Daily 1-hr maximum level (10 ppb) | Asthma ED visits | Excess risk=+2.7% (Lag 0-3: 1.5%, 3.9%) |
| Shmool et al. (2016)^236^ | New York/2005-2011 | Children's New York SPARCS data (n=2,353 (Hospitalizations) + 11,719 (ED visits)) | Daily average level (10 ppb) | Asthma ED visits, Hospitalizations | Hospitalization: % excess risk=6.5 (0.2, 13.1)-13 (6.0, 20.6) (lag 1-5); ED visits: % excess risk=2.9 (0.1, 5.7)-9.4 (6.3, 12.7) (lag 1-5) |
| Xiao et al. (2016)^237^ | Georgia/2002-2008 | Children ED visits (n=148, 256) | 3-day moving average (IQR: 18.5 ppb) | Asthma/Wheeze ED visits | OR=1.025 (1.007, 1.042) |
| Goodman et al. (2017)^238^ | New York/1999-2009 | New York SPARCS data (n=295,497) | Daily maximum 8-hr average level (10 ppb) | Asthma hospitalizations | All year: RR=1.0066 (0.9988, 1.0145), Warm season: RR=1.0068 (0.9969, 1.0167) |
| O' Lenick et al. (2017)^239^ | Atlanta/2002-2008, Dallas/2006-2008, St. Louis/2002-2007 | ED visit data (Not specified) | Daily maximum 8-hr average level (25 ppb) | Daily respiratory ED visit (Aggregated data: upper respiratory infections, bronchiolitis, pneumonia, asthma, and wheeze) | Atlanta: OR=1.08 (PI: 1.06, 1.11), Dallas: OR=1.04 (PI: 1.01, 1.07), St.Louis: OR=1.03 (PI: 0.99, 1.07) |
| Zu et al. (2017)^240^ | Texas/2001-2013 | Hospital admission data (n=28,488) | Daily maximum 8-hr average (10 ppb) | Asthma hospitalizations | All ages: RR=1.009 (Lag 0: 1.002, 1.016), 1.018 (Lag 1: 1.012, 1.025), 1.012 (Lag 2: 1.006, 1.019), 1.024 (Lag 0-3: 1.014, 1.034); 5-14 years: RR=1.017 (Lag 0: 1.003, 1.032), 1.034 (Lag 1: 1.020, 1.048), 1.024 (Lag 2: 1.010, 1.038), 1.047 (Lag 0-3: 1.025, 1.069); 15-64 years: RR=1.009 (Lag 0: 1.000, 1.019), 1.012 (Lag 1: 1.003, 1.021), 1.007 (Lag 2: 0.998, 1.016), 1.018 (Lag 0-3: 1.005, 1.032); 65+ years: RR=0.993 (Lag 0: 0.978, 1.007), 1.011 (Lag 1: 0.997, 1.025), 1.010 (Lag 2: 0.995, 1.024), 1.002 (Lag 0-3: 0.981, 1.02) |
| Castner et al. (2018)^241^ | Erie/2007-2012 | Asthma emergency visits data (n=76,651) | Daily maximum 8-hr average (0.017 ppm) | Daily count of asthma ED visits | ED visits=+4.68% (2.06%, 7.31%) |
| Krall et al. (2018)^242^ | 5 U.S. cities/2002-2008 | ED visits data (Not specified) | Daily maximum 8-hr average level (IQR: 25.34 ppb) | Asthma/Wheeze ED visits | Atlanta: RR=1.057 (Times-series: 1.004, 1.112), 1.055 (Multicity: 1.011, 1.102), 1.056 (MCM: 1.009, 1.105); Birmingham: RR = 1.113 (Times-series: 0.965, 1.284), 1.068 (Multicity: 0.983, 1.160), 1.070 (MCM: 0.983, 1.164); Dallas: RR = 1.011 (Times-series: 0.946, 1.080), 1.031 (Multicity: 0.975, 1.091), 1.028 (MCM: 0.971, 1.088); Pittsburgh: RR = 1.029 (Times-series: 0.946, 1.120), 1.043 (Multicity: 0.981, 1.110), 1.042 (MCM: 0.976, 1.112); St. Louis: RR = 1.094 (Times-series: 1.022, 1.171), 1.074 (Multicity: 1.014, 1.136), 1.077 (MCM: 1.017, 1.141) |
| Barry et al. (2019)^243^ | 5 U.S. cities/2002-2008 | ED visits data (Not specified) | Daily maximum 8-hr average level (25 ppb) | Asthma ED visits | Atlanta: RR=1.04 (1.02, 1.07), Birmingham: RR=1.04 (0.98, 1.11), Dallas: RR=1.04 (1.00, 1.08), Pittsburgh: RR=1.04 (1.01, 1.08), St.Louis: RR=1.07 (1.03, 1.11) |
| Gharibi et al. (2019)^244^ | Fresno/2015 | ED visits (n=1,101) | Daily maximum 8-hr average (18.1 ppb) | Asthma ED visits | OR=1.066 (1.032, 1.082) |
| Lovinsky-Desir et al. (2019)^245^ | New York City/2008-2011 | Children with asthma (n=190) | Annual average level (summer average: 1 ppb) | Asthma ED visits | Overall sample: PR=0.92 (0.79, 1.07), Lower asthma prevalence neighborhood: PR=0.56 (0.36, 0.87), Higher asthma prevalence neighborhood HAPN: PR=0.96 (0.84, 1.13) |
| Mendy et al. (2019)^246^ | U.S./2005-2006 | NHANES data (n=10,348) | Daily maximum 8-hr average (10 ppb) | Asthma ED visits | OR=1.07 (1.02, 1.13) |
| Reid et al. (2019)^247^ | California/2008 | Hospitalizations and ED visits data (median population size per ZIP codes=9,745) | Daily 8-hr maximum level (moving averages of the two days prior to the event) (10 ppb) | Asthma ED visits, Hospitalizations | Hospitalization: RR=0.939 (Before fires: 0.862, 1.023), RR=1.014 (During fires: 0.956, 1.076), RR=0.995 (After fires: 0.923, 1.072); ED visit: RR=0.971 (Before fires: 0.934, 1.008), RR=1.050 (During fires: 1.022, 1.078), RR=1.030 (After fires: 0.997, 1.064) |
| Witonsky et al. (2019)^248^ | Bronx/2001-2008 | Asthma patients’ data (n=43,729 visits) | 3-day moving average (Lag 0-2) (1 SD increase) | Daily asthma-related ED visits and hospitalizations | ED visits: β_Full year = 0.002 (p = 0.948), β_Winter = -0.240 (p = 0.000), β_Spring = -0.022 (p = 0.689), β_Summer = 0.112 (p = 0.024), β_Fall = -0.034 (p = 0.525); Hospital admissions: β_Full year = -0.065 (p = 0.073), β_Winter = 0.055 (p = 0.240), β_Spring = -0.143 (p = 0.015), β_Summer = 0.019 (p = 0.727), β_Fall = -0.082 (p = 0.164) |
| Baek et al. (2020)^249^ | South Texas/2010-2014 | Children with asthma (n=711) | Daily 8-hr average level (1 ppb) | Hospital length of stay | Lag 0: OR=1.014 (0.999, 1.029), Lag 0–1: OR=1.016 (1.000, 1.032), Lag 0–2: OR=1.018 (1.001, 1.036), Lag 0–3: OR=1.019 (1.000, 1.037), Lag 0–4: OR=1.018 (0.999, 1.038), Lag 0–5: OR=1.019 (0.998, 1.039), Lag 0–6: OR=1.019 (0.997, 1.041), Lag 0–7: OR=1.018 (0.996, 1.041) |
| Baek et al. (2020)^250^ | South Texas/2010-2014 | Children's re-admission data (n=111) | Daily maximum 8-hr average level (1 ppb) | Asthma readmission | Lag 0: OR=1.020 (0.999, 1.041), Lag 1: OR=1.008 (0.987, 1.028), Lag 2: OR=1.001 (0.979, 1.022), Lag 3: OR=1.007 (0.988, 1.027), Lag 0–1: OR=1.016 (0.993, 1.039), Lag 0–2: OR=1.013 (0.989, 1.038), Lag 0–3: OR=1.015 (0.988, 1.042) |
| Byrwa-Hill et al. (2020)^251^ | Allegheny/2008–2013 | ED visits data from Allegheny Health Network (n=6,682) | Average levels of lag days 0–5 (10 ppb) | Asthma ED visits | <Children> Lag 0: OR=1.000 (0.921, 1.086), Lag 1: OR=1.105 (1.017, 1.200), Lag 2: OR=1.017 (0.937, 1.103), Lag 3: OR=1.022 (0.943, 1.109), Lag 4: OR=1.048 (0.965, 1.139), Lag 5: OR=0.974 (0.898, 1.057), Lag 0–5: OR=1.075 (0.938, 1.231); <Adults> Lag 0: OR=0.968 (0.938, 1.000), Lag 1: OR=0.968 (0.938, 1.000), Lag 2: OR=0.959 (0.929, 0.990), Lag 3: OR=0.967 (0.937, 0.998), Lag 4: OR=0.969 (0.939, 1.000), Lag 5: OR=0.915 (0.867, 0.965), Lag 0–5: OR=0.981 (0.951, 1.013) |
| Rosenquist et al. (2020)^252^ | Nevada/2011-2017 | Children and Adults medical record (n=23,319) | Daily maximum 8-hour ozone level (For analysis, 3-day moving average ozone concentration was used) (20 ppb) | All asthma ED visit | OR=1.04 (1.04, 1.08) |
| Baek et al. (2022)^253^ | South Texas/2010-2016 | Children (n=902) | Yearly average level during study period (Quartile cutoffs were not specified) | Asthma hospitalization | Q2: OR=1.78 (1.01, 3.14), Q3: OR=1.43 (0.77, 2.67), Q4: OR=1.30 (0.64, 2.65) |
| Fecho et al. (2022)^254^ | North Carolina/2016 | Asthma patients (n=157,410) | Averaged daily maximum ozone level for 1 year study period (bin 1: 27.8-39.0 ppb, bin 2: 39.0-42.73 ppb, bin 3: 42.73-46.45 ppb) | Annual ED or inpatient visits | The proportion of individuals in that exposure level who had an ED visit due to asthma exacerbation: bin 1=8.9%, bin 2=15.4%, bin 3=19% ( χ2=102.6; p<0.001) |
| Park et al. (2022)^255^ | Louisiana/2010-2012 | Daily ED visits data from SharCor LLC (n=63,789) | Daily maximum ozone level (1 ppb) | Asthma daily ED visits | Same day visits with thunderstorm: RR=1.001 (0.998, 1.003), Next day visits with thunderstorm: RR=1.001 (0.997, 1.004), Same day visits without thunderstorm: RR=1.002 (1.001, 1.003), Next day visits without thunderstorm: No data |
| Rosser et al. (2022)^256^ | Allegheny/2010-2018 | Children's ED visits or hospitalization data from UPMC Children's hospital (n=6,573) | Daily AQI (10 AQI units) | Asthma ED visits | Lag 0: OR=0.99 (0.97, 1.01), Lag 1: OR=0.99 (0.97, 1.01), Lag 2: OR=1.01 (0.99, 1.03), Lag 3: OR=1.01 (0.99, 1.03), Lag 4: OR=1.00 (0.98, 1.02), Lag 5: OR=0.99 (0.97, 1.01) |
| Bi et al. (2023)^257^ | 10 U.S. states/2005-2014 | ED visit data from Environmental Exposures and Health Across the Nation (ENVISION) study (n=3,190,333) | Daily maximum 8-hour level (IQR: 0.02 ppm) | Asthma ED visits | Lags 0-2: RR=1.022 (1.014, 1.030), Lags 3-6: RR=1.002 (0.991, 1.012), Lags 0-7: RR=1.008 (0.995, 1.022) |
| Sharma et al. (2023)^258^ | New York City/2005-2011 | Children's Statewide Planning and Research Cooperative Systems (SPARCS) data (n=145,834 visits) | Daily 24-hr average level (10 ppb) | Asthma ED visits | Year-round: β=-1.10 (Lag 0: -1.99, -0.21), β=1.37 (Lag 1: 0.51, 2.23), β=1.84 (Lag 2: 1.04, 2.65), β=0.77 (Lag 3: -0.01, 1.55), β=-0.01 (Lag 4: -0.77, 0.77), β=-0.96 (Lag 5: -1.71, -0.20), β=-0.97 (Lag 6: -1.73, -0.20); Warm season: β=-1.07 (Lag 0: -2.37, 0.26), β=4.75 (Lag 1: 3.53, 5.97), β=4.66 (Lag 2: 3.54, 5.79), β=3.92 (Lag 3: 2.84, 5.00), β=1.60 (Lag 4: 0.56, 2.66), β=0.66 (Lag 5: -0.37, 1.70), β=-0.30 (Lag 6: -1.34, 0.74); Cold season: No data |
| Horne et al. (2024)^259^ | Utah’s Wasatch Front/1999-2022 | Asthma patients (n=63,976) | Daily maximum 8-hr average (10 ppb) | Asthma hospitalizations | Lag 0 – Wildfire OR=1.017 (0.981–1.053), Inversion OR=1.010 (0.982–1.039), All months OR=1.005 (0.985–1.025), Lag 1 – Wildfire OR=1.019 (0.983–1.056), Inversion OR=0.995 (0.967–1.024), All months OR=0.992 (0.973–1.012), Lag 2 – Wildfire OR=1.009 (0.973–1.046), Inversion OR=0.979 (0.952–1.007), All months OR=0.978 (0.959–0.998), mAvg 0–2 – Wildfire OR=1.027 (0.980–1.076), Inversion OR=1.003 (0.968–1.040), All months OR=0.988 (0.964–1.014), mAvg 0–6 – Wildfire OR=1.046 (0.981–1.116), Inversion OR=0.958 (0.911–1.006), All months OR=0.963 (0.932–0.996), mAvg 7–13 – Wildfire OR=1.045 (0.979–1.115), Inversion OR=0.975 (0.926–1.025), All months OR=0.972 (0.940–1.006), mAvg 14–20 – Wildfire OR=1.029 (0.967–1.094), Inversion OR=0.982 (0.934–1.032), All months OR=0.997 (0.964–1.006), mAvg 21–27 – Wildfire OR=1.014 (0.953–1.079), Inversion OR=1.014 (0.964–1.067), All months OR=1.009 (0.975–1.043) |

| **Table S3. Studies of Ozone Exposure and Other Respiratory-related Mortality and Morbidity (Not Included in the Main Text)** | | | | | |
| --- | --- | --- | --- | --- | --- |
| **Author (year)** | **Study region/year** | **Study population** | **Exposure metrix (per unit increase)** | **Health Outcome** | **Effect Estimate** |
| **Other respiratory-related mortality** | | | | | |
| Mills et al. (1991)^260^ | California/1977-1986 | California Seventh-day Adventists (n=6,340) | Average annual hours in excess of 10 pphm (500 hours/year) | Respiratory cancers mortality | RR=2.25 (0.96, 5.31) |
| Samet et al. (2000)^261^ | 20 counties/1987–1994 | National Center for Health Statistics data (n=+50 million) | 24-hour average level (10 ppb) | Cardio-pulmonary related mortality | Results were not specified (Ozone is not a significant predictor of death from cardio-respiratory causes) |
| Woodruff et al. (2008)^262^ | U.S. counties (with >250,000 residents)/1999–2002 | Infants (n=6,639 deaths) | 24-hour average level (10 ppb) | Respiratory mortality | OR=0.92 (0.76, 1.10) |
| Zanobetti et al. (2008)^263^ | 48 U.S. cities/1989-2000 | National Center for Health Statistics data (n=1,614,124 deaths) | Daily 8-h mean at lag 0, sum lags 0–20, 0–3, and 4–20 (10 ppb) | Respiratory mortality | Lag 0: 0.54% (0.26%, 0.81%), Sum lags 0-20: 0.61% (-0.41%, 1.65%), Sum lags 0-3: 0.83% (0.38%, 1.28%), Sum lags 4-20: -0.24% (-1.08%, 0.60%) |
| Zanobetti et al. (2011)^264^ | 105 U.S. cities/1985-2006 | Medicare data (n=3,210,511 COPD cases) | Yearly averages of the 8-hour mean daily level for summer (5 ppb) | Chronic Obstructive Pulmonary Disease (COPD) related mortality | HR=+1.07 (1.04, 1.09) (May to Sep) HR=+1.03 (1.00, 1.06) (Spring to Autumn) |
| Peng et al. (2013)^265^ | 86 U.S. cities in NMMAPS /1987–1997 | Daily mortality data (n=250,000 to over 9 million) | Daily 1-hour maximum level (average of lags 0 -1) (10 ppb) | Respiratory-related mortality | No association: Results were not specified because results for respiratory mortality were less consistent and not statistically significant in any case according to the author. |
| Xu et al. (2013)^266^ | Honolulu, Los Angeles/1992-2008 | Respiratory cancer cases (n=58,586) | Monthly average (10 ppb) | Respiratory cancer-specific death | Honolulu: HR=1.04 (1.03, 1.06), Los Angeles: HR=1.04 (1.02, 1.06) |
| Kravchenko et al. (2014)^267^ | North Carolina/1993–2010 | National Center for Health Statistics data (n=101,374 deaths were caused by pneumonia, 13,187 by emphysema, and 5,509 by asthma) | Month-specific average level (1 ppb) | Monthly death rate of emphysema, asthma, and pneumonia related death rates | Emphysema: beta 0.0061±0.0030 (p<0.05) Asthma: beta 0.0082±0.0056 (p>0.05) Respiratory: beta -0.0011±0.0019 (p>0.05) |
| Hao et al. (2015)^268^ | U.S./2007–2008 | National Center for Health Statistics data (n=265,223 deaths) | County-level average daily ozone (5 ppb) | CLRD (Chronic Lower Respiratory Disease) mortality | RR=1.05 (1.01, 1.09) |
| Turner et al. (2016)^269^ | 50 U.S. states, DC, Puerto Rico/1982-2004 | National Death Index and death certificates data (n=237,201 deaths/669,046 participants) | Daily 8-hour maximum level (10 ppb) | Respiratory, Pneumonia and influenza, COPD and allied conditions, and lung cancer related mortality | Respiratory: HR=1.12, Pneumonia and influenza: HR=1.10, COPD and allied conditions: HR=1.14, Lung cancer: HR=0.96 |
| Rush et al. (2017)^270^ | 47 U.S. states/2011 | Nationwide Inpatient Sample ARDS patients’ data (n=93,950) | Daily maximum 8-hr level (0.01 ppm) | In-hospital mortality for patients with ARDS | OR=1.07 (1.06-1.08) |
| Yang et al. (2024)^271^ | Southern California/2020-2021 | COVID cases (n= 773,374) and death (n=14,311) records | 1-year average (55ppb) | COVID-19 death | COVID-death: IRR=1.26 (95%IC: 1.18-1.36) |
| Azan et al. (2025)^272^ | NEW York City/2015-2020 | electronic health record (EHR) (not specified) | 11-year average (1ppb) | COVID-19 death | Overall: rate difference= + 0.55 per 10000 people (95%CI: 0.39-0.71 per 10000 people); Pandemic phase 1 (2015-2019): HR=1.06 (95CI: 1.04-1.08), Phase 2 (1/2020-12/2020): HR= 1.03 (95%CI: 1.00-1.06) |
| Kleeman et al. (2026)^273^ | Southern California/ 6/1/2020-1/1/2021 | COVID-19 cases (n=773,374) and deaths (14,311) that occurred during the study period in Los Angeles County | annual average (IQR=55ppb) | COVID-19 death | COVID-19 mortality: IRR=1.27 (95% CI: 1.17-1.36) |
| Luben et al. (2026)^274^ | North Carolina/2003-2015 | infant (n=37499, 30490 controls +3049 cases) | average concentration between birth and event | Respiratory infant mortality | OR=1.48 (95%CI: 0.72-3.00) |
| **Other respiratory-related morbidity** | | | | | |
| Lebowitz et al. (1987)^107^ | Arizona /1972-1974 | Adults with asthma (n=204) | Daily hourly maximum level (10 ppb) | Respiratory symptoms | Asthmatics: p < 0.05 (test statistics are not shown) Asymptomatics: p > 0.05 (test statistics are not shown) |
| Vedal et al. (1987)^108^ | Pennsylvania /1980-1981 | Children with asthma symptoms (n=4,000) | Daily average level (lagged by 24 and 48 hours) (25.5 ppb) | LRI, URI, Wheeze | No associations were found (The point estimates and confidence intervals were not reported) |
| Abbey et al. (1991)^275^ | California/1977-1987 | Seventh-day Adventist (n=3,914) | annual hours above multiple thresholds (500 hours above 10 pphm) | Bronchitis definite symptoms | RR=1.20 (0.97, 1.52) |
| Abbey et al. (1993)^276^ | California/1977-1987 | Seventh-day Adventist (n=3,914) | Annual average hours exceeding thresholds (threshold 10 pphm, each 500-h average annual increment) | Respiratory symptoms | RR=1.40 (0.99, 2.34) |
| Ostro et al. (1993)^138^ | Southern California /1978-1979 | Adults (n=321) | 1-hour maximum and 7-hour average levels (10 pphm) | Lower respiratory tract symptom | Lower respiratory symptoms_1-hr level: OR = 1.22 (1.11, 1.34), Lower respiratory symptoms_7-hr level: OR = 1.32 (1.14, 1.52) |
| Schwartz (1994)^277^ | Alabama/1986-1989 | Medicare data (n=not specified) | Daily 24-h average level (lagged) (50 ppb) | Respiratory hospital admissions | Pneumonia: RR = 1.14 (0.94, 1.38) COPD: RR = 1.17 (0.86, 1.60) |
| Schwartz et al. (1994)^278^ | 6 U.S. cities /1984-1988 | Children (n=1,844) | Daily mean level (30 ppb) | Cough | OR=1.27 (1.05, 1.54) |
| Abbey et al. (1995)^164^ | California/1977-1987 | Seventh-day Adventist (n=3,914) | Days & hours above threshold levels (10, 12, 15, 20, 25 ppm) | Chronic bronchitis, Respiratory cancer incidence | Chronic bronchitis: RR=1.02 (0.83, 1.25), Respiratory cancer incidence: RR=2.25 (0.96, 5.31) |
| Jones et al. (1995)^279^ | Louisiana/1990 | Three local hospital patients ED visit data (n=1,265) | Daily hourly ozone levels | Adult respiratory-related ED visits | β=0.269 (p<0.04) |
| Neas et al. (1995)^116^ | Pennsylvania /1990 | Children (n=83) | 12-hour average level | Respiratory symptoms (evening cough) | cough: OR = 1.6 (1.1, 2.4) |
| Schwartz (1996)^280^ | Spokane/1988-1990 | U.S. Health Care Financing Administration repots data (n=4,241 respiratory hospital admissions) | Daily 24-h average level based on peak-hour level (25.5 ppb) | Respiratory hospital admissions | RR = 1.244 (1.002, 1.544) |
| Moolgavkar et al. (1997)^281^ | Minneapolis-St. Paul, Birmingham/1986-1991 | U.S. Health Care Financing Administration record (n=not specified) | Daily 24-h average level (lags 0-2 days) (15 ppb) | Respiratory hospital admissions | % increase: 5.15 (2.36, 7.94) |
| Beeson et al. (1998)^282^ | California /1977-1992 | Adults (n=6,338) | Monthly average daily 8-hr level (IQR: 100 ppb) | Lung cancer incidence | RR=3.56 (1.35, 9.42) |
| McConnell et al. (1999)^283^ | California /1993 | Children (n=3,676) | yearly average daily maximum 1-hr level (32 ppb) | Bronchitis, Phlegm, Cough | Asthma: Bronchitis OR=1.0 (95% CI = 0.6-1.7) Phlegm OR = 1.2 (95% CI = 0.5-3.1) Cough OR = 1.1 (95%CI = 0.6-2.0) Wheeze/no asthma: Bronchitis OR=1.1 (95% CI = 0.7-1.6) Phlegm OR = 0.8 (95% CI = 0.5-1.4) Cough OR = 0.7 (95%CI = 0.5-1.1) No wheeze/no asthma: Bronchitis OR=0.9 (95% CI = 0.4-1.8) Phlegm OR = 0.8 (95% CI = 0.5-1.5) Cough OR = 0.9 (95%CI = 0.6-1.3) |
| Peters et al. (1999)^284^ | Southern California/1993 | Children (n = 3,600) | Hourly average level (40 ppb) | Respiratory symptoms and illnesses (ever asthma, current asthma, wheeze, cough, and bronchitis) | Bronchitis: OR = 1.14 (95%CI: 0.84, 1.55) Cough: OR = 0.98 (95%CI: 0.82, 1.17) Wheeze: OR = 1.08 (95%CI: 0.87, 1.35) |
| Linn et al. (2000)^285^ | Los Angeles/1992-1995 | California Office of Statewide Health Planning and Development (OSHPD) Hospitalization data (not specified the sample size) | 24-hr average level (1 pphm) | Pulmonary hospitalizations | All seasons: β=0.008 (p=0.004), Winter: β=-0.006 (p=0.010), Spring: β=0.011 (p=0.008), Summer: β=0.003 (p=0.007), Autumn: β=0.009 (p=0.011) |
| Moolgavkar (2000)^286^ | Cook County, Los Angeles County, Maricopa County/1987-1995 | Hospital admissions data from Health Care Financing Administration (not specified the sample size) | Daily average level (Lag 0-5) (10 ppb) | COPD related hospitalizations | % change of daily COPD admission; Cook county: 0.5 (p>0.05, Lag 0), 2.0 (p<0.05, Lag 1), 2.2 (p<0.05, Lag 2), 1.5 (p>0.05, Lag 3), 1.0 (p>0.05, Lag 4), -1.1 (p>0.05, Lag 5); Los Angeles county: 3.2 (p<0.05, Lag 0), 3.0 (p<0.05, Lag 1), 2.0 (p<0.05, Lag 2), 1.3 (p<0.05, Lag 3), 1.0 (p>0.05, Lag 4), 0.3 (p>0.05, Lag 5); Maricopa county: –0.4 (p>0.05, Lag 0), 0.3 (p>0.05, Lag 1), 1.1 (p>0.05, Lag 2), 1.5 (p>0.05, Lag 3), –0.5 (p>0.05, Lag 4), –0.1 (p>0.05, Lag 5) |
| Moolgavkar et al. (2000)^287^ | Washington/1987-1995 | Comprehensive Hospital Abstract Reporting System data (n=daily hospital admissions range from 0 - 23) | Daily 24-h average level (lagged) (1 ppm) | Chronic respiratory hospital admissions | Log RR * 100 = -1.2 (p>0.05, Lag 0), 0.8 (p>0.05, Lag 1), 0.0 (p>0.05, Lag 2), 1.0 (p>0.05, Lag 3), –2.0 (p>0.05, Lag 4); values were approximated from the figure since detailed numerical results were not reported. |
| Mortimer et al. (2000)^126^ | 8 urban areas /1993 | Children (n=846) | Multiday moving average level (lags 1 - 5 days) (15 ppb) | Respiratory symptoms | Normal birthweight and full term: OR = 1.09 (0.95, 1.24) Low birthweight or premature: OR = 1.42 (1.10, 1.82) |
| Gwynn and Thurston (2001)^288^ | New York/1988-1990 | Statewide Planning and Research Cooperative System (SPARCS) Hospital admissions data (n=1,096 admissions per day) | Daily average level (58.6 ppb) | Respiratory hospital admissions | White: RR = 1.032 (0.977, 1.089) Non-white: RR = 1.122 (1.074, 1.172) |
| Ross et al. (2002)^289^ | Illinois/1994 | Asthmatic residents (n=59) | Daily average 8 hr maximum level with lag days (20 ppb) | Respiratory symptom score | AM: 0.14 (0.08, 0.21) PM: 0.11 (0.05, 0.16)  Asthma medication use: 0.10 (0.02, 0.18) |
| McConnell et al. (2003)^290^ | California/1993 | Children (n=225) | Annual averages of 8 hours level (10am - 6pm) (1 ppb) | Bronchitic symptoms | Within community: OR = 1.06 (1.00, 1.12) Between community: OR = 0.99 (0.98–1.01) |
| Medina-Ramón et al. (2006)^291^ | 36 U.S. cities/1986-1999 | Children (n=225) | Daily maximum 8-hr average level (lags 0-1; cumulative) (5 ppb) | Respiratory hospital admissions | <% increase> Pneumonia: 0.27 (0.08, 0.47)  COPD: 0.41 (0.26, 0.57) |
| Triche et al. (2006)^292^ | Virginia/1994-1996 | Infants (n=691) | 24-hr average (IQR: 11.8 ppb), peak 1-hr (IQR: 18.0 ppb), and maximum 8-hr running average level (0–1-day lag) (IQR: 19.0 ppb) | Infant respiratory symptoms | 24-hr average Wheeze: OR=1.32 (0.91, 1.92), 24-hr average difficulty breathing: OR=1.10 (0.69, 1.75), 8-hr max Wheeze: OR=1.04 (0.76, 1.43), 8-hr max difficulty breathing: OR=1.17 (0.77, 1.77), peak 1-hr_Wheeze: OR=1.00 (0.77, 1.31), peak 1-hr_difficulty breathing: OR=1.09 (0.78, 1.52) |
| Karr et al. (2007)^293^ | South Coast Air Basin of California/1995–2000 | Infants (n=18,595 cases, n=169,472 controls) | Daily 8-hr average max level (14 ppb) | Respiratory hospitalization for bronchiolitis | Subchronic exposure: OR = 0.92 (0.88, 0.97) Chronic exposure: OR = 0.92 (0.88, 0.97) |
| Lin et al. (2008)^294^ | New York State/1991-2001 | New York State Department of Health (NYSDOH) – Statewide Planning and Research Cooperative System (SPARCS) (n=134,099) | Daily maximum 8-hr average level (1-3 days lags) (IQR: 23 ppb) | Daily respiratory hospital admissions | % increase: 1.75 (1.01, 2.48) |
| Parker et al. (2009)^295^ | U.S./1999–2005 | Children (n=73,198) | Summer average of daily 8-hr level (10 ppb) | Reporting of respiratory allergy/hay fever | OR=1.20 (1.15-1.26) |
| Choi et al. (2011)^296^ | Maryland/2002 | ED visits data (n=452) | Three days average level of 8-hour max level (10 ppb) | Respiratory ED visits | Increase of the percentage change in respiratory ED visits: +2.4% (1.06%, 3.76%) |
| Darrow et al. (2011)^297^ | Atlanta/1993-2004 | ED visit data (n=1,068,525 visits) | Daily maximum 8-hr average level (28.9 ppb) | Respiratory emergency department visits | RR=1.020 (1.012, 1.028) |
| Peel et al. (2011)^298^ | Egleston/1998-2002 | Infants (n=4,277) | 8-hr maximum level (25 ppb) | Apnea (prolonged pauses in breathing) | OR=1.036 (0.987, 1.088) |
| Berhane et al. (2016)^299^ | 8 Southern California communities/1993 - 2001, 1996-2004, 2003 - 2012 | Children (n=4,602) | Community-level annual average level (10 AM–6 PM) (per median reduction: 3.6 ppb) | Annual age-specific prevalence of bronchitic symptoms | Children with asthma: OR=0.66 (0.50, 0.86), children without asthma: OR=0.85 (0.74, 0.97) |
| Ware et al. (2016)^300^ | Nashville/2006-2012 | ICU patients (n=1,558) | 3-year average level (5 ppb) | Acute Respiratory Distress Syndrome (ARDS) risk | 95% CI=1.27, 1.96 |
| Sack et al. (2017)^301^ | Baltimore, Chicago, Los Angeles, New York, St Paul, Winston Salem /2000/2001/2002 - 2010/2011/2012 | Adults (n=5,495) | 10-year average level (10 ppb) | Interstitial lung abnormalities (ILAs) and High attenuation areas | ILAs in never-smokers: OR = 0.3 (0.10, 0.93) High attenuation areas: % increase = 0.10 (−0.49, 0.71) |
| Pearce et al. (2018)^302^ | South Carolina/2002–2013 | Residents of Columbia (n=2,192,170 cardiorespiratory events) | Daily 8-hr max level (lags 0 -3 days) (20 ppb) | Upper respiratory infection hospital admissions | Asthma: RR=1.8% (1.1, 2.2), URI: RR=2.2% (1.8, 4.1) |
| Pirozzi et al. (2018)^303^ | Utah’s Wasatch Front/2009-2012 | 7 Wasatch Front Intermountain Healthcare ED visit data (n=4,336 visits) | Daily maximum 8-hr average (lag days 1-7) (10 ppb) | Severe pneumonia | <65 adults: OR=1.02 (1.01, 1.04) >=65 adults: OR=1.00 (0.99, 1.01) |
| Seeni et al. (2018)^304^ | 12 U.S. cities/2002-2008 | Newborn (n=223,375) | level of 3 months' preconception (90 days before conception, IQR: 12.30 ppb), trimester one (gestational weeks 1-13, IQR: 12.32 ppb), trimester two (gestational weeks 14-28, IQR: 11.96 ppb), and the whole pregnancy (conception to birth, IQR: 7.83 ppb) | Transient tachypnea of the newborn ^305^, Asphyxia, Respiratory distress syndrome (RDS) | <3 months preconception> TTN: RR = 0.97 (95% CI: 0.91, 1.04), Asphyxia: RR = 1.76 (95% CI: 1.25, 2.48), RDS: RR = 1.09 (95% CI: 1.01, 1.18) <Trimester 1> TTN: RR = 0.98 (95% CI: 0.91, 1.06), Asphyxia: RR = 1.72 (95% CI: 1.32, 2.24), RDS: RR = 1.21 (95% CI: 1.12, 1.31) <Trimester 2> TTN: RR = 0.94 (95% CI: 0.87, 1.01), Asphyxia: RR = 1.34 (95% CI: 1.03, 1.75), RDS: RR = 1.13 (95% CI: 1.04, 1.22) <Whole pregnancy> TTN: RR = 0.97 (95% CI: 0.89, 1.05), Asphyxia: RR = 1.73 (95% CI: 1.24, 2.42), RDS: RR = 1.16 (95% CI: 1.05, 1.29) |
| Glick et al. (2019)^306^ | U.S./2007-2008 | NIS data (n=57,972 with pneumonia) | Daily maximum 8-hr average level (at +70 ppb and +60 ppb) | Length of stay (hospitalization) due to pneumonia | >70ppb: +0.19 (0.05, 0.33), >60ppb: +0.04 (-0.02, 0.10) |
| Reilly et al. (2019)^307^ | Pennsylvania/2005-2015 | ICU Patients (n=996) | Daily 8-hr max O₃ (daytime/summer only) with 3-day lag, 6-wk average, 3-yr average (1 ppb) | Acute Respiratory Distress Syndrome (ARDS) | 3-year: OR=1.44 (1.12, 1.86) 6-week: OR=1.13 (0.88, 1.46) 3-day: OR=0.90 (0.64, 1.26) |
| Yazdi et al. (2019)^308^ | Florida, Alabama, Mississippi, Georgia, North Carolina, South Carolina, Tennessee/2000-2012 | Medicare data (n=11,084,660) | Annual average level (1 ppm) | Hospitalization for COPD and pneumonia | COPD: RR = 1.024 (1.023, 1.025), Pneumonia: RR=1.030 (1.029, 1.031) |
| Cromar et al. (2020)^309^ | California/2012-2014 | California’s Office of Statewide Health Planning and Development data (n=1,373,165 respiratory emergency department visits) | Daily maximum 8-hr average level (March–October_IQR (AQI): <Southern California> 18, <San Joaquin Valley> 21, <San Francisco Bay Area> 12 November–February_IQR (AQI): <Southern California> 8, <San Joaquin Valley> 13, <San Francisco Bay Area> 11) | Respiratory ED visits | < Southern California > March–October_RR: Lag 0 = 1.004 (1.000–1.009), Lag 1 = 1.005 (1.000–1.010), Lag 2 = 1.003 (0.998–1.007), Lag 3 = 1.004 (1.000–1.009), Lag 4 = 1.003 (0.998–1.008), Lag 5 = 0.999 (0.995–1.004), November–February_RR: Lag 0 = 1.004 (1.000–1.009), Lag 1 = 1.007 (1.002–1.012), Lag 2 = 1.006 (1.002–1.011), Lag 3 = 1.006 (1.001–1.011), Lag 4 = 1.000 (0.996–1.005), Lag 5 = 0.994 (0.989–0.999)  < San Joaquin Valley > March–October_RR: Lag 0 = 0.988 (0.974–1.003), Lag 1 = 0.983 (0.968–0.998), Lag 2 = 0.989 (0.974–1.003), Lag 3 = 0.996 (0.981–1.010), Lag 4 = 0.991 (0.977–1.004), Lag 5 = 0.982 (0.969–0.994), November–February_RR: Lag 0 = 1.024 (1.014–1.034), Lag 1 = 1.021 (1.011–1.030), Lag 2 = 1.019 (1.010–1.028), Lag 3 = 1.016 (1.007–1.025), Lag 4 = 1.011 (1.002–1.019), Lag 5 = 1.007 (0.998–1.016)  < San Francisco Bay Area > March–October_RR: Lag 0 = 1.009 (1.001–1.016), Lag 1 = 1.012 (1.005–1.019), Lag 2 = 1.012 (1.005–1.019), Lag 3 = 1.014 (1.006–1.021), Lag 4 = 1.007 (1.001–1.014), Lag 5 = 1.007 (1.001–1.013), November–February_RR: Lag 0 = 0.994 (0.983–1.005), Lag 1 = 1.008 (0.996–1.020), Lag 2 = 1.009 (0.998–1.021), Lag 3 = 1.008 (0.997–1.019), Lag 4 = 1.010 (1.000–1.021), Lag 5 = 1.004 (0.993–1.015) |
| Fuller et al. (2020)^310^ | Washington, DC/2013-2014 | Kaiser Permanente Mid-Atlantic States dataset (n=~700,000) | Daily maximum 8-hr average level (lagged) (10 ppb) | Respiratory related healthcare utilization | <% increase> Nurse calls/emails: 2.95 (1.93, 3.96) ED/UC visits: 1.56 (0.38, 2.74)  Provider visits: 1.10 (0.48, 1.73) Hospital admissions: −2.13 (95% CI: -4.73, 0.47) |
| Steurer et al. (2020)^311^ | California/2007-2012 | Preterm infants (n=205,178) | 3-year average based on daily maximum 8-hr average level (Not specified, author used quartile) | Respiratory hospital admissions | Unadjusted RR = 1.29 (1.21, 1.37), adjusted RR=1.02 (0.95, 1.10) |
| Yazdi et al. (2021)^312^ | U.S./2000-2016 | Medicare data (n=63,006,793) | Annual average level (1 ppb) | Pneumonia related hospital admissions | Median risk difference: 0.00413% (0.00376%, 0.00447%) |
| Belz et al. (2022)^313^ | Several locations in the U.S./2010-2015 | Adults (n=1,874) | 10-year average level (5 ppb) | Respiratory morbidity | high poverty areas: 𝛽=0.97 (0.25, 1.69) medium poverty areas: 𝛽=0.61 (0.004, 1.23)  low poverty areas: 𝛽=2397 (-0.35, 0.87) |
| Yazdi et al. (2022)^314^ | U.S./2001-2016 | Medicare beneficiaries’ data (n=436,684) | 2-year average levels (1 ppb) | Respiratory-related hospital admissions | RD=9.51/100,000p-y (6.59, 12.13) |
| Aglan et al. (2023)^315^ | Boston/2017-2019 | Patients with COPD (n=30) | Previous-day 24-hr community-level (IQR: 11.8 ppb) | Any respiratory symptoms | Community level: OR=1.35 (1.07, 1.70) |
| Elam et al. (2023)^316^ | U.S. military Periodic Health Assessment locations/2015-2018 | Patients with CRS (military treatment facilities) (n=399) | 1 year mean level before CRS diagnosis (5 ppb) | Chronic rhinosinusitis (CRS) | OR=1.66 (0.73, 3.74) |
| Reilly et al. (2023)^305^ | Philadelphia Metropolitan/2008-2018 | Patients with septic (n=1,858) | Daily (daytime/summer only) level with 3-day lag (13.7 ppb), 6-wk average (7.6 ppb), 5-yr average (2.0 ppb) | ARDS risk | 3-day: OR=1.22 (0.98, 1.52), 6-week: OR=1.24 (1.00, 1.54), 3-yr: OR=1.09 (0.96, 1.24) |
| Garcia et al. (2024)^317^ | California /1992-1993, 1995-1996, 2002-2003 | Adults (n=1,308) | Annual average level based on daily 8-hour maximum level (10 ppb) | Adult bronchitic symptoms | Not having childhood asthma: OR=0.83 (0.60, 1.15), Having childhood asthma: OR=1.02 (0.67, 1.53) |
| Yang et al. (2024)^271^ | Southern California/2020-2021 | COVID cases (n= 773,374) and death (n=14,311) records | 1-year average (55ppb) | COVID-19 cases | COVID: IRR=1.15 (95%CI:1.06-1.18) |
| Aguilera et al. (2025)^318^ | California/2006–2019 | Respiratory hospitalizations (n=18,185,219 from 1,147 CA ZIP codes) | Daily wildfire-specific O₃ level (IQR=8.5ppb) | Respiratory hospital admissions (rate per 100,000 people) | <Respiratory hospital admissions> all months β=-0.002847 (95% CI: -0.006593–0.0008241); excluding summer β=-0.004735 (95% CI: -0.01079–0.001304); summer only β=0.006001 (95% CI: 0.001910–0.01011) |
| Ferschl et al (2025)^319^ | San Francisco, CA/2019-2023 | Pediatric patients (18 and younger) | 15-day average before procedure: high (highest AQI >100) and low (highest AQI ≤100) O3 exposure | Intraoperative or immediate postoperative adverse respiratory events including bronchospasm, laryngospasm, and reintubation | RR=1.43 (95%CI: 1.02-2.03) |
| Kannoth et al. (2025)^320^ | New York City/2020-2021 | electronic health record (EHR) (not specified) | 11-year average (1ppb, highest versus lowest quantile) | COVID-19 related severe morbidity | Phase I (3/2020-6/2020): <ARDS> RR=1.02 (95%CI: 0.99-1.05), <Dialysis> RR=1.02 (95%CI: 0.91-1.14), <Length of stay> RR=0.99 (95%CI: 0.98-1.00), <Pneumonia> RR=1.00 (95%CI: 0.98-1.02), <Ventilation> RR=1.01 (95%CI: 0.99-1.01); Phase 2&3 (7/2020-3/2021): <ARDS> RR=1.04 (95%CI: 1.00-1.08), <Dialysis> RR=1.13 (95%CI: 0.96-1.34), <Length of stay> RR=0.99 (95%CI: 0.98-1.00), <Pneumonia> RR=1.08 (95%CI: 1.06-1.10), <Ventilation> RR=1.11 (95%CI: 1.07-1.16). |
| Kronzer et al. (2025)^120^ | USA /2014-2018 | US veterans (n=78553) | 1-3,3-5 and 5+ years average (per 10ppb increase and Highest vs lowest quartile) | Rheumatoid Arthritis–Associated Interstitial Lung Disease (RA-ILD) | Overall RA-ILD: OR=0.90 (95%CI: 0.67-1.19) per 10ppb increase; OR=0.89 (95% CI: 0.65–1.21) (highest vs. lowest quantile) |
| Park et al. (2025)^321^ | California/1993-2015 | Adults (n=38,654) | time-varying monthly average (IQR=10ppb) | Incident COPD | HR=0.76 (95%CI: 0.68-0.84). For O3, the association became positive when accounting for NOx |
| Stingone et al. (2025)^322^ | New York City/2020-2021 | Patients hospitalized with COVID-19 (n=20,318) | Residential ZIP code–level mean ambient O₃ concentration over 2009–2019 (1ppb) | COVID-19–related ARDS and pneumonia during hospitalization | <ARDS> Phase 1: RR=1.00 (95% CI: 0.98–1.02), catchment subset RR=0.96 (0.94–0.98); Phases 2–3: RR=1.03 (1.00–1.06), catchment subset RR=1.03 (0.99–1.06); <Pneumonia> Phase 1: RR=0.98 (0.96–1.00), catchment subset RR=0.83 (0.81–0.86); Phases 2–3: RR=1.07 (1.06–1.09), catchment subset RR=1.09 (1.07–1.11); <Mechanical ventilation> Phase 1: RR=1.03 (1.01–1.05), catchment subset RR=1.05 (1.02–1.08); Phases 2–3: RR=1.11 (1.07–1.15), catchment subset RR=1.18 (1.12–1.24). |
| Chen et al. (2026)^323^ | South California/1992-2003 | Adults (n=1444) | yearly average | bronchitic symptoms | Increased risk associated with O3 exposure at ages 4–5 and 12–15 years with the highest at age 14 years: RR=1.51 (95% CI: 1.23-1.86), negative associations were observed for O3 at ages 8–11 years with the strongest at age 10 years: RR = 0.80 (95% CI: 0.71, 0.90) |
| Kleeman et al. (2026)^273^ | Southern California/ 6/1/2020-1/1/2021 | COVID-19 cases (n=773,374) and deaths (14,311) that occurred during the study period in Los Angeles County | annual average (IQR=55ppb) | COVID-19-related outcomes | COVID-19 incidence: IRR=1.12 (95% CI: 1.06-1.18) |
| Ou et al. (2026)^324^ | Utah/2000-2016 | Testicular cancer survivors/15-39 (n=385) | Daily maximum 8-hour level 1-4 days before visit (1ppb) | Respiratory encounters | Lag 1: <Any respiratory encounter> OR=1.01 (95% CI: 1.00–1.02), <Respiratory ED/urgent care> OR=1.01 (95% CI: 1.00–1.02), <Respiratory inpatient> OR=1.00 (95% CI: 0.97–1.02); Lag 2: <Any respiratory encounter> OR=1.01 (95% CI: 1.00–1.02), <Respiratory ED/urgent care> OR=1.01 (95% CI: 1.00–1.02), <Respiratory inpatient> OR=1.00 (95% CI: 0.97–1.02); Lag 3: <Any respiratory encounter> OR=0.99 (95% CI: 0.98–1.00), <Respiratory ED/urgent care> OR=0.99 (95% CI: 0.98–1.00), <Respiratory inpatient> OR=0.99 (95% CI: 0.96–1.01); Lag 4: <Any respiratory encounter> OR=1.00 (95% CI: 0.99–1.01), <Respiratory ED/urgent care> OR=1.00 (95% CI: 0.99–1.01), <Respiratory inpatient> OR=1.01 (95% CI: 0.99–1.03) |
| Ramsay et al. (2026)^325^ | Utah/1998-2016 | adolescent and young adult cancer survivors (n=8016) | across the 6 days before the event (lag day 1 to lag day 6, per 10ppb increase) | respiratory healthcare visits (inpatient admissions and ED visits) | Inpatient encounters: lag day 4: OR=1.01 (95% CI:0.90-1.08); ED visits: lag day 4: OR=1.01 (95%CI: 0.98-1.03) |
| Zhang et al. (2026)^326^ | 20-county Atlanta/1999-2019 | Respiratory ED visits at 41 hospitals (not specified) | Population-weighted 3-day moving average (lags 0–2) of the daily 8-hour maximum level (IQR=24.9ppb) | Combined respiratory ED visits (asthma/wheeze, bronchiolitis, COPD, pneumonia, and upper respiratory infections) | <Respiratory ED visits> conventional quasi-Poisson RR=1.039 (95% CI: 1.027–1.051); constant-dispersion DGLM RR=1.037 (95% CI: 1.024–1.050); time-varying-dispersion DGLM RR=1.029 (95% CI: 1.020–1.039). |
| Zhao et al. (2026)^327^ | Florida, USA/2005-2016 | ED visits records (n = 79378186) | Daily average (10ppb) | Respiratory related ED visits | Percentage change: + 3.32% (95%CI: 2.88-3.77%) |

**<Experimental study>**

Figure S1-1. Quality Assessment of Experimental Studies of Ozone Exposure and Pulmonary Function. Note: the quality of each paper was accessed using the Joanna Briggs Institute (JBI) critical appraisal tools. Studies were classified as: Low risk of bias (good quality) when both reviewers’ “% Yes” and the inter-reviewer agreement were ≥70%; Moderate risk of bias (moderate quality) when any of these metrics fell between 60% and <70%; and High risk of bias (poor quality) when any metric was <60%.

Figure S1-1. (Continued). Quality Assessment of Experimental Studies of Ozone Exposure and Pulmonary Function. Note: the quality of each paper was accessed using the Joanna Briggs Institute (JBI) critical appraisal tools. Studies were classified as: Low risk of bias (good quality) when both reviewers’ “% Yes” and the inter-reviewer agreement were ≥70%; Moderate risk of bias (moderate quality) when any of these metrics fell between 60% and <70%; and High risk of bias (poor quality) when any metric was <60%.

Figure S1-1. (Continued). Quality Assessment of Experimental Studies of Ozone Exposure and Pulmonary Function. Note: the quality of each paper was accessed using the Joanna Briggs Institute (JBI) critical appraisal tools. Studies were classified as: Low risk of bias (good quality) when both reviewers’ “% Yes” and the inter-reviewer agreement were ≥70%; Moderate risk of bias (moderate quality) when any of these metrics fell between 60% and <70%; and High risk of bias (poor quality) when any metric was <60%.

**<Cohort study>**

Figure S1-2. Quality Assessment of Observational Studies of Ozone Exposure and Pulmonary Function. Note: the quality of each paper was accessed using the Joanna Briggs Institute (JBI) critical appraisal tools. Studies were classified as: Low risk of bias (good quality) when both reviewers’ “% Yes” and the inter-reviewer agreement were ≥70%; Moderate risk of bias (moderate quality) when any of these metrics fell between 60% and <70%; and High risk of bias (poor quality) when any metric was <60%.

**<Cohort study>**

**<Cross-sectional study>**

Figure S1-2. (Continued) Quality Assessment of Observational Studies of Ozone Exposure and Pulmonary Function. Note: the quality of each paper was accessed using the Joanna Briggs Institute (JBI) critical appraisal tools. Studies were classified as: Low risk of bias (good quality) when both reviewers’ “% Yes” and the inter-reviewer agreement were ≥70%; Moderate risk of bias (moderate quality) when any of these metrics fell between 60% and <70%; and High risk of bias (poor quality) when any metric was <60%.

**<Cohort study>**

**<Case-control study>**

Figure S2-1. Quality Assessment of studies for Ozone Exposure and Asthma-related symptoms. Note: the quality of each paper was accessed using the Joanna Briggs Institute (JBI) critical appraisal tools. Studies were classified as: Low risk of bias (good quality) when both reviewers’ “% Yes” and the inter-reviewer agreement were ≥70%; Moderate risk of bias (moderate quality) when any of these metrics fell between 60% and <70%; and High risk of bias (poor quality) when any metric was <60%.

**<Cross-sectional study>**

Figure S2-1. (Continued) Quality Assessment of studies for Ozone Exposure and Asthma-related symptoms. Note: the quality of each paper was accessed using the Joanna Briggs Institute (JBI) critical appraisal tools. Studies were classified as: Low risk of bias (good quality) when both reviewers’ “% Yes” and the inter-reviewer agreement were ≥70%; Moderate risk of bias (moderate quality) when any of these metrics fell between 60% and <70%; and High risk of bias (poor quality) when any metric was <60%.

**<Cohort study>**

**<Case-control study>**

**<Cross-sectional study>**

Figure S2-2. Quality Assessment of studies for Ozone Exposure and Asthma-related ED visits and Hospitalizations. Note: the quality of each paper was accessed using the Joanna Briggs Institute (JBI) critical appraisal tools. Studies were classified as: Low risk of bias (good quality) when both reviewers’ “% Yes” and the inter-reviewer agreement were ≥70%; Moderate risk of bias (moderate quality) when any of these metrics fell between 60% and <70%; and High risk of bias (poor quality) when any metric was <60%.

**<Cross-sectional study>**

**Figure S2-2. (Continued) Quality Assessment of studies for Ozone Exposure and Asthma-related ED visits and Hospitalizations. Note: the quality of each paper was accessed using the Joanna Briggs Institute (JBI) critical appraisal tools. Studies were classified as: Low risk of bias (good quality) when both reviewers’ “% Yes” and the inter-reviewer agreement were ≥70%; Moderate risk of bias (moderate quality) when any of these metrics fell between 60% and <70%; and High risk of bias (poor quality) when any metric was <60%.**

**<Cohort study>**

**<Case-control study>**

**<Cross-sectional study>**

Figure S3-1. Quality Assessment of studies for Ozone Exposure and Other Respiratory-related Mortality. Note: the quality of each paper was accessed using the Joanna Briggs Institute (JBI) critical appraisal tools. Studies were classified as: Low risk of bias (good quality) when both reviewers’ “% Yes” and the inter-reviewer agreement were ≥70%; Moderate risk of bias (moderate quality) when any of these metrics fell between 60% and <70%; and High risk of bias (poor quality) when any metric was <60%.

**<Cohort study>**

**<Case-control study>**

**Figure S3-2. Quality Assessment of Studies for Ozone Exposure and Other Respiratory-related Morbidity.** **Note: the quality of each paper was accessed using the Joanna Briggs Institute (JBI) critical appraisal tools. Studies were classified as: Low risk of bias (good quality) when both reviewers’ “% Yes” and the inter-reviewer agreement were ≥70%; Moderate risk of bias (moderate quality) when any of these metrics fell between 60% and <70%; and High risk of bias (poor quality) when any metric was <60%.**

**<Cross-sectional study>**

**Figure S3-2. (Continued) Quality Assessment of Studies for Ozone Exposure and Other Respiratory-related Morbidity. Note: the quality of each paper was accessed using the Joanna Briggs Institute (JBI) critical appraisal tools. Studies were classified as: Low risk of bias (good quality) when both reviewers’ “% Yes” and the inter-reviewer agreement were ≥70%; Moderate risk of bias (moderate quality) when any of these metrics fell between 60% and <70%; and High risk of bias (poor quality) when any metric was <60%.**

**References**

1.	Folinsbee LJ, Bedi JF, Horvath SM. Respiratory responses in humans repeatedly exposed to low concentrations of ozone. *Am Rev Respir Dis*. 1980;doi:10.1164/arrd.1980.121.3.431

2.	Linn WS, Jones MP, Bachmayer EA, et al. Short-term respiratory effects of polluted ambient air: a laboratory study of volunteers in a high-oxidant community. *Am Rev Respir Dis*. 1980;

3.	Adams WC, Savin WM, Christo AE. Detection of ozone toxicity during continuous exercise via the effective dose concept. *J Appl Physiol Respir Environ Exerc Physiol*. 1981;doi:10.1152/jappl.1981.51.2.415

4.	Dimeo MJ, Glenn MG, Holtzman MJ, Sheller JR, Nadel JA, Boushey HA. THRESHOLD CONCENTRATION OF OZONE CAUSING AN INCREASE IN BRONCHIAL REACTIVITY IN HUMANS AND ADAPTATION WITH REPEATED EXPOSURES. *American Review of Respiratory Disease*. 1981;

5.	Folinsbee LJ, Bedi JF, Horvath SM. Combined effects of ozone and nitrogen dioxide on respiratory function in man. *Am Ind Hyg Assoc J*. 1981;doi:doi: 10.1080/15298668191420206.

6.	Horvath SM, Gliner JA, Folinsbee LJ. ADAPTATION TO OZONE - DURATION OF EFFECT. *American Review of Respiratory Disease*. 1981;

7.	Mihevic PM, Gliner JA, Horvath SM. Perception of effort and respiratory sensitivity during exposure to ozone. *Ergonomics*. 1981;doi:10.1080/00140138108924858

8.	Linn WS, Medway DA, Anzar UT, et al. Persistence of adaptation to ozone in volunteers exposed repeatedly for six weeks. *Am Rev Respir Dis*. 1982;

9.	Linn WS, Fischer DA, Medway DA, et al. Short-term respiratory effects of 0.12 ppm ozone exposure in volunteers with chronic obstructive pulmonary disease. *Am Rev Respir Dis*. 1982;

10.	Solic JJ, Hazucha MJ, Bromberg PA. The acute effects of 0.2 ppm ozone in patients with chronic obstructive pulmonary disease. *Am Rev Respir Dis*. 1982;doi:DOI:?10.1164/arrd.1982.125.6.664

11.	Adams WC, Schelegle ES. Ozone and high ventilation effects on pulmonary function and endurance performance. *J Appl Physiol Respir Environ Exerc Physiol*. 1983;doi:10.1152/jappl.1983.55.3.805

12.	Avol EL, Linn WS, Shamoo DA, Venet TG, Hackney JD. ACUTE RESPIRATORY EFFECTS OF LOS-ANGELES SMOG IN CONTINUOUSLY EXERCISING ADULTS. *Journal of the Air Pollution Control Association*. 1983;

13.	Gliner JA, Horvath SM, Folinsbee LJ. Preexposure to low ozone concentrations does not diminish the pulmonary function response on exposure to higher ozone concentrations. *Am Rev Respir Dis*. 1983;doi:10.1164/arrd.1983.127.1.51

14.	Linn WS, Shamoo DA, Venet TG, et al. Response to ozone in volunteers with chronic obstructive pulmonary disease. *Arch Environ Health*. 1983;doi:10.1080/00039896.1983.10544007

15.	McDonnell WF, Horstman DH, Hazucha MJ, et al. Pulmonary effects of ozone exposure during exercise: dose-response characteristics. *J Appl Physiol Respir Environ Exerc Physiol*. May 1983;54(5):1345-52. doi:10.1152/jappl.1983.54.5.1345

16.	Avol EL, Linn WS, Venet TG, Shamoo DA, Hackney JD. Comparative respiratory effects of ozone and ambient oxidant pollution exposure during heavy exercise. *J Air Pollut Control Assoc*. 1984;

17.	Superko HR, Adams WC, Daly PW. Effects of ozone inhalation during exercise in selected patients with heart disease. *Am J Med*. 1984;doi:10.1016/0002-9343(84)90105-0

18.	Avol EL, Linn WS, Shamoo DA, et al. Respiratory effects of photochemical oxidant air pollution in exercising adolescents. *Am Rev Respir Dis*. 1985;doi:10.1164/arrd.1985.132.3.619

19.	Bedi JF, Drechsler-Parks DM, Horvath SM. Duration of increased pulmonary function sensitivity to an initial ozone exposure. *Am Ind Hyg Assoc J*. 1985;doi:10.1080/15298668591395643

20.	Kehrl HR, Hazucha MJ, Solic JJ, Bromberg PA. Responses of subjects with chronic obstructive pulmonary disease after exposures to 0.3 ppm ozone. *Am Rev Respir Dis*. 1985;

21.	Koenig JQ, Covert DS, Morgan MS, et al. Acute effects of 0.12 ppm ozone or 0.12 ppm nitrogen dioxide on pulmonary function in healthy and asthmatic adolescents. *Am Rev Respir Dis*. 1985;doi:DOI:?10.1164/arrd.1985.132.3.648

22.	Kulle TJ, Sauder LR, Hebel JR, Chatham MD. Ozone response relationships in healthy nonsmokers. *Am Rev Respir Dis*. 1985;

23.	Lauritzen SK, Adams WC. OZONE INHALATION EFFECTS CONSEQUENT TO CONTINUOUS EXERCISE IN FEMALES - COMPARISON TO MALES. *Journal of Applied Physiology*. 1985;

24.	W. F. McDonnell r, Horstman DH, Abdul-Salaam S, House DE. Reproducibility of individual responses to ozone exposure. *Am Rev Respir Dis*. 1985;doi:DOI:?10.1164/arrd.1985.131.S5.S36

25.	W. F. McDonnell r, Chapman RS, Leigh MW, Strope GL, Collier AM. Respiratory responses of vigorously exercising children to 0.12 ppm ozone exposure. *Am Rev Respir Dis*. 1985;doi:DOI:?10.1164/arrd.1985.132.4.875

26.	H. Gong J, Bradley PW, Simmons MS, Tashkin DP. Impaired exercise performance and pulmonary function in elite cyclists during low-level ozone exposure in a hot environment. *Am Rev Respir Dis*. 1986;doi:DOI:?10.1164/arrd.1986.134.4.726

27.	Horvath SM, Bedi JF, Drechsler-Parks DM. Effects of peroxyacetyl nitrate alone and in combination with ozone in healthy young women. *J Air Pollut Control Assoc*. 1986;doi:10.1080/00022470.1986.10466067

28.	Linn WS, Avol EL, Shamoo DA, et al. A dose-response study of healthy, heavily exercising men exposed to ozone at concentrations near the ambient air quality standard. *Toxicol Ind Health*. 1986;doi:10.1177/074823378600200105

29.	Schelegle ES, Adams WC. Reduced exercise time in competitive simulations consequent to low level ozone exposure. *Med Sci Sports Exerc*. Aug 1986;18(4):408-14.

30.	Avol EL, Linn WS, Shamoo DA, et al. SHORT-TERM RESPIRATORY EFFECTS OF PHOTOCHEMICAL OXIDANT EXPOSURE IN EXERCISING CHILDREN. *Japca-the International Journal of Air Pollution Control and Hazardous Waste Management*. 1987;

31.	Drechsler-Parks DM, Bedi JF, Horvath SM. Pulmonary function desensitization on repeated exposures to the combination of peroxyacetyl nitrate and ozone. *Japca*. 1987;doi:10.1080/08940630.1987.10466316

32.	Horvath SM, Folinsbee LJ, Bedi JF. Combined effect of ozone and sulfuric acid on pulmonary function in man. *Am Ind Hyg Assoc J*. 1987;doi:10.1080/15298668791384454

33.	Kehrl HR, Vincent LM, Kowalsky RJ, et al. Ozone exposure increases respiratory epithelial permeability in humans. *Am Rev Respir Dis*. 1987;

34.	Koenig JQ, Covert DS, Marshall SG, Belle GV, Pierson WE. The effects of ozone and nitrogen dioxide on pulmonary function in healthy and in asthmatic adolescents. *Am Rev Respir Dis*. 1987;doi:DOI:?10.1164/ajrccm/136.5.1152

35.	McDonnell WF, Horstman DH, Abdul-Salaam S, Raggio LJ, Green JA. The respiratory responses of subjects with allergic rhinitis to ozone exposure and their relationship to nonspecific airway reactivity. *Toxicol Ind Health*. 1987;doi:10.1177/074823378700300405

36.	Bedi JF, Horvath SM, Drechsler-Parks DM. Reproducibility of the pulmonary function response of older men and women to a 2-hour ozone exposure. *Japca*. 1988;doi:10.1080/08940630.1988.10466442

37.	Folinsbee LJ, McDonnell WF, Horstman DH. Pulmonary function and symptom responses after 6.6-hour exposure to 0.12 ppm ozone with moderate exercise. *JAPCA*. Jan 1988;38(1):28-35. doi:10.1080/08940630.1988.10466349

38.	Koenig JQ, Covert DS, Smith MS, Belle Gv, Pierson WE. The pulmonary effects of ozone and nitrogen dioxide alone and combined in healthy and asthmatic adolescent subjects. *Toxicol Ind Health*. 1988;doi:10.1177/074823378800400410

39.	Linn WS, Avol EL, Shamoo DA, et al. Repeated laboratory ozone exposures of volunteer Los Angeles residents: an apparent seasonal variation in response. *Toxicol Ind Health*. 1988;doi:10.1177/074823378800400409

40.	Reisenauer CS, Koenig JQ, McManus MS, Smith MS, Kusic G, Pierson WE. Pulmonary response to ozone exposures in healthy individuals aged 55 years or greater. *Japca*. 1988;doi:10.1080/08940630.1988.10466353

41.	Bedi JF, Horvath SM, Drechsler-Parks DM. Adaptation by older individuals repeatedly exposed to 0.45 parts per million ozone for two hours. *Japca*. 1989;doi:10.1080/08940630.1989.10466521

42.	Brookes KA, Adams WC, Schelegle ES. O.35 ppm O3 exposure induces hyperresponsiveness on 24-h reexposure to 0.20 ppm O3. *J Appl Physiol (1985)*. 1989;doi:10.1152/jappl.1989.66.6.2756

43.	Drechsler-Parks DM, Bedi JF, Horvath SM. Pulmonary function responses of young and older adults to mixtures of O3, NO2 and PAN. *Toxicol Ind Health*. 1989;doi:10.1177/074823378900500310

44.	Koren HS, Devlin RB, Graham DE, et al. Ozone-induced inflammation in the lower airways of human subjects. *Am Rev Respir Dis*. Feb 1989;139(2):407-15. doi:10.1164/ajrccm/139.2.407

45.	Schelegle ES, Adams WC, Giri SN, Siefkin AD. ACUTE OZONE EXPOSURE INCREASES PLASMA PROSTAGLANDIN-F-2-ALPHA IN OZONE-SENSITIVE HUMAN-SUBJECTS. *American Review of Respiratory Disease*. 1989;

46.	Schonfeld BR, Adams WC, Schelegle ES. Duration of enhanced responsiveness upon re-exposure to ozone. *Arch Environ Health*. 1989;doi:DOI:?10.1080/00039896.1989.9935888

47.	Drechsler-Parks DM, Horvath SM, Bedi JF. "The effective dose"" concept in older adults exposed to ozone""". *Exp Gerontol*. 1990;doi:10.1016/0531-5565(90)90041-y

48.	Horstman DH, Folinsbee LJ, Ives PJ, Abdul-Salaam S, McDonnell WF. Ozone concentration and pulmonary response relationships for 6.6-hour exposures with five hours of moderate exercise to 0.08, 0.10, and 0.12 ppm. *Am Rev Respir Dis*. 1990;doi: 10.1164/ajrccm/142.5.1158

49.	Koenig JQ, Covert DS, Hanley QS, Belle Gv, Pierson WE. Prior exposure to ozone potentiates subsequent response to sulfur dioxide in adolescent asthmatic subjects. *Am Rev Respir Dis*. 1990;doi:DOI:?10.1164/ajrccm/141.2.377

50.	Messineo TD, Adams WC. Ozone inhalation effects in females varying widely in lung size: comparison with males. *J Appl Physiol (1985)*. 1990;doi:10.1152/jappl.1990.69.1.96

51.	Aris R, Christian D, Sheppard D, Balmes JR. THE EFFECTS OF SEQUENTIAL EXPOSURE TO ACIDIC FOG AND OZONE ON PULMONARY-FUNCTION IN EXERCISING SUBJECTS. *American Review of Respiratory Disease*. 1991;

52.	Emmons K, Foster WM. Smoking cessation and acute airway response to ozone. *Arch Environ Health*. 1991;doi:DOI:?10.1080/00039896.1991.9934389

53.	Koren HS, Devlin RB, Becker S, Perez R, McDonnell WF. Time-dependent changes of markers associated with inflammation in the lungs of humans exposed to ambient levels of ozone. *Toxicol Pathol*. 1991;doi:10.1177/0192623391019004-109

54.	McDonnell WF, Kehrl HR, Abdul-Salaam S, et al. Respiratory response of humans exposed to low levels of ozone for 6.6 hours. *Arch Environ Health*. 1991;doi:10.1080/00039896.1991.9937441

55.	Schelegle ES, Siefkin AD, McDonald RJ. Time course of ozone-induced neutrophilia in normal humans. *Am Rev Respir Dis*. 1991;doi:DOI:?10.1164/ajrccm/143.6.1353

56.	Hazucha MJ, Folinsbee LJ, Seal E, Jr. Effects of steady-state and variable ozone concentration profiles on pulmonary function. *Am Rev Respir Dis*. Dec 1992;146(6):1487-93. doi:10.1164/ajrccm/146.6.1487

57.	Foster WM, Silver JA, Groth ML. Exposure to ozone alters regional function and particle dosimetry in the human lung. *J Appl Physiol (1985)*. 1993;doi:10.1152/jappl.1993.75.5.1938

58.	Fox SD, Adams WC, Brookes KA, Lasley BL. Enhanced response to ozone exposure during the follicular phase of the menstrual cycle. *Environ Health Perspect*. 1993;

59.	Seal E, Jr., McDonnell WF, House DE, et al. The pulmonary response of white and black adults to six concentrations of ozone. *Am Rev Respir Dis*. Apr 1993;147(4):804-10. doi:10.1164/ajrccm/147.4.804

60.	Basha MA, Gross KB, Gwizdala CJ, Haidar AH, Popovich J, Jr. Bronchoalveolar lavage neutrophilia in asthmatic and healthy volunteers after controlled exposure to ozone and filtered purified air. *Chest*. Dec 1994;106(6):1757-65. doi:10.1378/chest.106.6.1757

61.	Folinsbee LJ, Horstman DH, Kehrl HR, Harder S, Abdul-Salaam S, Ives PJ. Respiratory responses to repeated prolonged exposure to 0.12 ppm ozone. *Am J Respir Crit Care Med*. 1994;doi:10.1164/ajrccm.149.1.8111607

62.	Gerrity TR, McDonnell WF, House DE. The relationship between delivered ozone dose and functional responses in humans. *Toxicol Appl Pharmacol*. 1994;doi:10.1006/taap.1994.1033

63.	Koenig JQ, Covert DS, Pierson WE, et al. "Oxidant and acid aerosol exposure in healthy subjects and subjects with asthma. Part I: Effects of oxidants, combined with sulfuric or nitric acid, on the pulmonary function of adolescents with asthma". *Res Rep Health Eff Inst*. 1994;

64.	Weymer AR, Gong H, Jr., Lyness A, Linn WS. Pre-exposure to ozone does not enhance or produce exercise-induced asthma. *Am J Respir Crit Care Med*. Jun 1994;149(6):1413-9. doi:10.1164/ajrccm.149.6.8004292

65.	Drechsler-Parks DM. The dose-response relationship in older men exposed to ozone. *Exp Gerontol*. 1995;doi:10.1016/0531-5565(94)00051-4

66.	Frampton MW, Morrow PE, Cox C, et al. Sulfuric acid aerosol followed by ozone exposure in healthy and asthmatic subjects. *Environ Res*. Apr 1995;69(1):1-14. doi:10.1006/enrs.1995.1020

67.	Horstman DH, Ball BA, Brown J, Gerrity T, Folinsbee LJ. Comparison of pulmonary responses of asthmatic and nonasthmatic subjects performing light exercise while exposed to a low level of ozone. *Toxicol Ind Health*. 1995;doi:10.1177/074823379501100401

68.	Linn WS, Anderson KR, Shamoo DA, et al. Controlled exposures of young asthmatics to mixed oxidant gases and acid aerosol. *Am J Respir Crit Care Med*. Sep 1995;152(3):885-91. doi:10.1164/ajrccm.152.3.7663800

69.	McDonnell WF, Stewart PW, Andreoni S, Smith MV. Proportion of moderately exercising individuals responding to low-level, multi-hour ozone exposure. *Am J Respir Crit Care Med*. 1995;doi:10.1164/ajrccm.152.2.7633712

70.	McKittrick T, Adams WC. Pulmonary function response to equivalent doses of ozone consequent to intermittent and continuous exercise. *Arch Environ Health*. 1995;doi:10.1080/00039896.1995.9940892

71.	Weinmann GG, Weidenbach-Gerbase M, Foster WM, Zacur H, Frank R. Evidence for ozone-induced small-airway dysfunction: lack of menstrual-cycle and gender effects. *Am J Respir Crit Care Med*. 1995;doi: 10.1164/ajrccm.152.3.7663815

72.	Weinmann GG, Liu MC, Proud D, Weidenbachgerbase M, Hubbard W, Frank R. "OZONE EXPOSURE IN HUMANS - INFLAMMATORY, SMALL AND PERIPHERAL AIRWAY RESPONSES". *American Journal of Respiratory and Critical Care Medicine*. 1995;

73.	Balmes JR, Chen LL, Scannell C, et al. Ozone-induced decrements in FEV1 and FVC do not correlate with measures of inflammation. *Am J Respir Crit Care Med*. 1996;doi:10.1164/ajrccm.153.3.8630571

74.	Devlin RB, McDonnell WF, Becker S, et al. Time-dependent changes of inflammatory mediators in the lungs of humans exposed to 0.4 ppm ozone for 2 hr: a comparison of mediators found in bronchoalveolar lavage fluid 1 and 18 hr after exposure. *Toxicol Appl Pharmacol*. 1996;doi:10.1006/taap.1996.0111

75.	Foster WM, Jiang L, Stetkiewicz PT, Risby TH. Breath isoprene: temporal changes in respiratory output after exposure to ozone. *J Appl Physiol (1985)*. 1996;doi:<https://journals.physiology.org/doi/epdf/10.1152/jappl.1996.80.2.706>

76.	Foster WM, Stetkiewicz PT. Regional clearance of solute from the respiratory epithelia: 18-20 h postexposure to ozone. *J Appl Physiol (1985)*. 1996;doi:<https://journals.physiology.org/doi/epdf/10.1152/jappl.1996.81.3.1143>

77.	Scannell C, Chen L, Aris RM, et al. Greater ozone-induced inflammatory responses in subjects with asthma. *Am J Respir Crit Care Med*. 1996;doi:10.1164/ajrccm.154.1.8680687

78.	Balmes JR, Aris RM, Chen LL, et al. Effects of ozone on normal and potentially sensitive human subjects. Part I: Airway inflammation and responsiveness to ozone in normal and asthmatic subjects. *Res Rep Health Eff Inst*. Jun 1997;(78):1-37; discussion 81-99.

79.	Frampton MW, Morrow PE, Torres A, et al. Ozone responsiveness in smokers and nonsmokers. *American Journal of Respiratory and Critical Care Medicine*. 1997;

80.	Frampton MW, Morrow PE, Torres A, et al. Effects of ozone on normal and potentially sensitive human subjects. Part II: Airway inflammation and responsiveness to ozone in nonsmokers and smokers. *Res Rep Health Eff Inst*. 1997;

81.	Gong H, Jr., McManus MS, Linn WS. Attenuated response to repeated daily ozone exposures in asthmatic subjects. *Arch Environ Health*. Jan-Feb 1997;52(1):34-41. doi:10.1080/00039899709603797

82.	McDonnell WF, Stewart PW, Andreoni S, et al. Prediction of ozone-induced FEV1 changes. Effects of concentration, duration, and ventilation. *Am J Respir Crit Care Med*. Sep 1997;156(3 Pt 1):715-22. doi:10.1164/ajrccm.156.3.9611044

83.	Peden DB, Boehlecke B, Horstman D, Devlin R. Prolonged acute exposure to 0.16 ppm ozone induces eosinophilic airway inflammation in asthmatic subjects with allergies. *J Allergy Clin Immunol*. 1997;

84.	Christian DL, Chen LL, Scannell CH, Ferrando RE, Welch BS, Balmes JR. Ozone-induced inflammation is attenuated with multiday exposure. *Am J Respir Crit Care Med*. 1998;doi:10.1164/ajrccm.158.2.9709023

85.	Gong H, Wong R, Sarma RJ, et al. Cardiovascular effects of ozone exposure in human volunteers. *American Journal of Respiratory and Critical Care Medicine*. 1998;

86.	Gong H, Simmons MS, Linn WS, McDonnell WF. Relationship between acute ozone responsiveness and chronic loss of lung function in residents of a high-ozone community. *Archives of Environmental Health*. 1998;

87.	Frampton MW, Pryor WA, Cueto R, Cox C, Morrow PE, Utell MJ. Ozone exposure increases aldehydes in epithelial lining fluid in human lung. *American Journal of Respiratory and Critical Care Medicine*. 1999;

88.	Kehrl HR, Peden DB, Ball B, Folinsbee LJ, Horstman D. Increased specific airway reactivity of persons with mild allergic asthma after 7.6 hours of exposure to 0.16 ppm ozone. *J Allergy Clin Immunol*. 1999;doi:10.1016/s0091-6749(99)70013-8

89.	McDonnell WF, Stewart PW, Smith MV, Pan WK, Pan J. Ozone-induced respiratory symptoms: exposure-response models and association with lung function. *Eur Respir J*. 1999;doi:DOI:?10.1034/j.1399-3003.1999.14d21.x

90.	Adams WC. Feasibility study of prolonged ozone inhalation exposure via face mask. *Inhal Toxicol*. 2000;doi:10.1080/089583700196167

91.	Folinsbee LJ, Hazucha MJ. Time course of response to ozone exposure in healthy adult females. *Inhal Toxicol*. 2000;doi:10.1080/089583700196211

92.	Foster WM, Brown RH, Macri K, Mitchell CS. Bronchial reactivity of healthy subjects: 18-20 h postexposure to ozone. *J Appl Physiol (1985)*. 2000;doi:10.1152/jappl.2000.89.5.1804

93.	Altman MC, Kattan M, O'Connor GT, et al. Associations between outdoor air pollutants and non-viral asthma exacerbations and airway inflammatory responses in children and adolescents living in urban areas in the USA: a retrospective secondary analysis. *Lancet Planet Health*. Jan 2023;7(1):e33-e44. doi:10.1016/s2542-5196(22)00302-3

94.	Frank R, Liu MC, Spannhake EW, Mlynarek S, Macri K, Weinmann GG. Repetitive ozone exposure of young adults: evidence of persistent small airway dysfunction. *Am J Respir Crit Care Med*. 2001;doi:10.1164/ajrccm.164.7.2010043

95.	Hazucha MJ, Folinsbee LJ, Bromberg PA. Distribution and reproducibility of spirometric response to ozone by gender and age. *J Appl Physiol (1985)*. 2003;doi:10.1152/japplphysiol.00490.2003

96.	Chen LL, Tager IB, Peden DB, et al. Effect of ozone exposure on airway responses to inhaled allergen in asthmatic subjects. *Chest*. 2004;doi:10.1378/chest.125.6.2328

97.	Arjomandi M, Witten A, Abbritti E, et al. Repeated exposure to ozone increases alveolar macrophage recruitment into asthmatic airways. *Am J Respir Crit Care Med*. 2005;doi:10.1164/rccm.200502-272OC

98.	Brown JS, Bateson TF, McDonnell WF. Effects of exposure to 0.06 ppm ozone on FEV1 in humans: a secondary analysis of existing data. *Environ Health Perspect*. Aug 2008;116(8):1023-6. doi:10.1289/ehp.11396

99.	Schelegle ES, Morales CA, Walby WF, Marion S, Allen RP. 6.6-hour inhalation of ozone concentrations from 60 to 87 parts per billion in healthy humans. *Am J Respir Crit Care Med*. Aug 1 2009;180(3):265-72. doi:10.1164/rccm.200809-1484OC

100.	Madden MC, Stevens T, Case M, et al. Diesel exhaust modulates ozone-induced lung function decrements in healthy human volunteers. *Part Fibre Toxicol*. 2014;doi:10.1186/s12989-014-0037-5

101.	Kariisa M, Foraker R, Pennell M, et al. Short- and long-term effects of ambient ozone and fine particulate matter on the respiratory health of chronic obstructive pulmonary disease subjects. *Arch Environ Occup Health*. 2015;70(1):56-62. doi:10.1080/19338244.2014.932753

102.	Habre R, Zhou H, Eckel SP, et al. Short-term effects of airport-associated ultrafine particle exposure on lung function and inflammation in adults with asthma. *Environ Int*. 2018;doi:10.1016/j.envint.2018.05.031

103.	Rich DQ, Thurston SW, Balmes JR, et al. Do Ambient Ozone or Other Pollutants Modify Effects of Controlled Ozone Exposure on Pulmonary Function? *Ann Am Thorac Soc*. 2020;doi:10.1513/AnnalsATS.201908-597OC

104.	Wilgus ML, Edmonds A, Keller KP, et al. Greater ambient air pollution exposure is associated with worse respiratory symptoms in men and women with HIV and chronic lung disease: a cohort study. *Respir Res*. Dec 19 2025;26(1):349. doi:10.1186/s12931-025-03398-0

105.	Lebowitz MD, Holberg CJ, Boyer B, Hayes C. Respiratory symptoms and peak flow associated with indoor and outdoor air pollutants in the southwest. *J Air Pollut Control Assoc*. Nov 1985;35(11):1154-8. doi:10.1080/00022470.1985.10466016

106.	Lioy PJ, Vollmuth TA, Lippmann M. Persistence of peak flow decrement in children following ozone exposures exceeding the National Ambient Air Quality Standard. *J Air Pollut Control Assoc*. 1985;

107.	Lebowitz MD, Collins L, Holberg CJ. Time series analyses of respiratory responses to indoor and outdoor environmental phenomena. *Environ Res*. 1987;doi:10.1016/s0013-9351(87)80033-6

108.	Vedal S, Schenker MB, Munoz A, Samet JM, Batterman S, Speizer FE. Daily air pollution effects on children's respiratory symptoms and peak expiratory flow. *Am J Public Health*. 1987;doi:10.2105/ajph.77.6.694

109.	Spektor DM, Lippmann M, Thurston GD, et al. Effects of ambient ozone on respiratory function in healthy adults exercising outdoors. *Am Rev Respir Dis*. 1988;doi:DOI:?10.1164/ajrccm/138.4.821

110.	Dockery DW, Speizer FE, Stram DO, Ware JH, Spengler JD, B. G. Ferris J. Effects of inhalable particles on respiratory health of children. *Am Rev Respir Dis*. 1989;doi:10.1164/ajrccm/139.3.587

111.	Kinney PL, Ware JH, Spengler JD, Dockery DW, Speizer FE, B. G. Ferris J. Short-term pulmonary function change in association with ozone levels. *Am Rev Respir Dis*. 1989;doi:10.1164/ajrccm/139.1.56

112.	Berry M, Lioy PJ, Gelperin K, Buckler G, Klotz J. Accumulated exposure to ozone and measurement of health effects in children and counselors at two summer camps. *Environ Res*. Apr 1991;54(2):135-50. doi:10.1016/s0013-9351(05)80096-9

113.	Brunekreef B, Kinney PL, Ware JH, et al. Sensitive subgroups and normal variation in pulmonary function response to air pollution episodes. *Environ Health Perspect*. 1991;doi:10.1289/ehp.90-1519500

114.	Spektor DM, Thurston GD, Mao J, He D, Hayes C, Lippmann M. Effects of single- and multiday ozone exposures on respiratory function in active normal children. *Environ Res*. 1991;doi:10.1016/s0013-9351(05)80167-7

115.	Krzyzanowski M, Quackenboss JJ, Lebowitz MD. Relation of peak expiratory flow rates and symptoms to ambient ozone. *Arch Environ Health*. 1992;doi:10.1080/00039896.1992.10118763

116.	Neas LM, Dockery DW, Koutrakis P, Tollerud DJ, Speizer FE. The association of ambient air pollution with twice daily peak expiratory flow rate measurements in children. *Am J Epidemiol*. 1995;doi:DOI:?10.1093/oxfordjournals.aje.a117399

117.	Kinney PL, Nilsen DM, Lippmann M, et al. Biomarkers of lung inflammation in recreational joggers exposed to ozone. *American Journal of Respiratory and Critical Care Medicine*. 1996;

118.	Raizenne M, Neas LM, Damokosh AI, et al. Health effects of acid aerosols on North American children: pulmonary function. *Environ Health Perspect*. May 1996;104(5):506-14. doi:10.1289/ehp.96104506

119.	Kunzli N, Lurmann F, Segal M, Ngo L, Balmes J, Tager IB. Association between lifetime ambient ozone exposure and pulmonary function in college freshmen - Results of a pilot study. *Environmental Research*. 1997;

120.	Kronzer VL, Yang Y, Roul P, et al. Associations of Fire Smoke and Other Pollutants With Incident Rheumatoid Arthritis and Rheumatoid Arthritis-Associated Interstitial Lung Disease. *Arthritis & Rheumatology*. Jul 2025;77(7):808-816. doi:10.1002/art.43113

121.	Galizia A, Kinney PL. Long-term residence in areas of high ozone: Associations with respiratory health in a nationwide sample of nonsmoking young adults. *Environmental Health Perspectives*. 1999;

122.	Naeher LP, Holford TR, Beckett WS, et al. Healthy women's PEF variations with ambient summer concentrations of PM10, PM2.5, SO42-, H+, and O3. *American Journal of Respiratory and Critical Care Medicine*. 1999;doi:10.1164/ajrccm.160.1.9808153

123.	Peters JM, Avol E, Gauderman WJ, et al. A study of twelve southern California communities with differing levels and types of air pollution - II. Effects on pulmonary function. *American Journal of Respiratory and Critical Care Medicine*. 1999;

124.	Gauderman WJ, McConnell R, Gilliland F, et al. Association between air pollution and lung function growth in southern California children. *Am J Respir Crit Care Med*. Oct 2000;162(4 Pt 1):1383-90. doi:10.1164/ajrccm.162.4.9909096

125.	Kinney PL, Lippmann M. Respiratory effects of seasonal exposures to ozone and particles. *Arch Environ Health*. 2000;doi:10.1080/00039890009603408

126.	Mortimer KM, Tager IB, Dockery DW, Neas LM, Redline S. The effect of ozone on inner-city children with asthma: identification of susceptible subgroups. *Am J Respir Crit Care Med*. 2000;doi:10.1164/ajrccm.162.5.9908113

127.	Avol EL, Gauderman WJ, Tan SM, London SJ, Peters JM. Respiratory effects of relocating to areas of differing air pollution levels. *Am J Respir Crit Care Med*. Dec 1 2001;164(11):2067-72. doi:10.1164/ajrccm.164.11.2102005

128.	Mortimer KM, Neas LM, Dockery DW, Redline S, Tager IB. The effect of air pollution on inner-city children with asthma. *Eur Respir J*. Apr 2002;19(4):699-705. doi:10.1183/09031936.02.00247102

129.	Delfino RJ, H. Gong J, Linn WS, Pellizzari ED, Hu Y. Asthma symptoms in Hispanic children and daily ambient exposures to toxic and criteria air pollutants. *Environ Health Perspect*. 2003;doi:10.1289/ehp.5992

130.	Lewis TC, Robins TG, Dvonch JT, et al. Air pollution-associated changes in lung function among asthmatic children in Detroit. *Environ Health Perspect*. Aug 2005;113(8):1068-75. doi:10.1289/ehp.7533

131.	Qian Z, Liao D, Lin HM, Whitsel EA, Rose KM, Duan Y. Lung function and long-term exposure to air pollutants in middle-aged American adults. *Arch Environ Occup Health*. 2005;doi:10.3200/aeoh.60.3.156-163

132.	Tager IB, Balmes J, Lurmann F, Ngo L, Alcorn S, Künzli N. Chronic exposure to ambient ozone and lung function in young adults. *Epidemiology*. Nov 2005;16(6):751-9. doi:10.1097/01.ede.0000183166.68809.b0

133.	Girardot SP, Ryan PB, Smith SM, et al. Ozone and PM2.5 exposure and acute pulmonary health effects: a study of hikers in the Great Smoky Mountains National Park. *Environ Health Perspect*. 2006;doi:10.1289/ehp.8637

134.	Alexeeff SE, Litonjua AA, Suh H, Sparrow D, Vokonas PS, Schwartz J. Ozone exposure and lung function: effect modified by obesity and airways hyperresponsiveness in the VA normative aging study. *Chest*. Dec 2007;132(6):1890-7. doi:10.1378/chest.07-1126

135.	Gauderman WJ, Vora H, McConnell R, et al. Effect of exposure to traffic on lung development from 10 to 18 years of age: a cohort study. *The Lancet*. 2007/02/17/ 2007;369(9561):571-577. doi:10.1016/S0140-6736(07)60037-3

136.	Islam T, Gauderman WJ, Berhane K, et al. Relationship between air pollution, lung function and asthma in adolescents. *Thorax*. Nov 2007;62(11):957-63. doi:10.1136/thx.2007.078964

137.	Alexeeff SE, Litonjua AA, Wright RO, et al. "Ozone exposure, antioxidant genes, and lung function in an elderly cohort: VA normative aging study". *Occupational and Environmental Medicine*. 2008;doi:10.1136/oem.2007.035253

138.	Ostro BD, Lipsett MJ, Mann JK, Krupnick A, Harrington W. Air pollution and respiratory morbidity among adults in southern California. *Am J Epidemiol*. 1993;doi:DOI:?10.1093/oxfordjournals.aje.a116729

139.	Mortimer K, Neugebauer R, Lurmann F, Alcorn S, Balmes J, Tager I. Air pollution and pulmonary function in asthmatic children: effects of prenatal and lifetime exposures. *Epidemiology*. 2008;doi:10.1097/EDE.0b013e31816a9dcb

140.	Thaller EI, Petronella SA, Hochman D, Howard S, Chhikara RS, Brooks EG. Moderate increases in ambient PM2.5 and ozone are associated with lung function decreases in beach lifeguards. *J Occup Environ Med*. 2008;doi:10.1097/JOM.0b013e31816386b4

141.	Berhane K, Zhang Y, Linn WS, et al. The effect of ambient air pollution on exhaled nitric oxide in the Children's Health Study. *Eur Respir J*. 2011;doi:10.1183/09031936.00081410

142.	Eckel SP, Louis TA, Chaves PH, Fried LP, Margolis AH. Modification of the association between ambient air pollution and lung function by frailty status among older adults in the Cardiovascular Health Study. *Am J Epidemiol*. 2012;doi:10.1093/aje/kws001

143.	Rice MB, Ljungman PL, Wilker EH, et al. Short-Term Exposure to Air Pollution and Lung Function in the Framingham Heart Study. *American Journal of Respiratory and Critical Care Medicine*. 2013;doi:10.1164/rccm.201308-1414OC

144.	Berhane K, Zhang Y, Salam MT, et al. Longitudinal effects of air pollution on exhaled nitric oxide: the Children's Health Study. *Occup Environ Med*. 2014;doi:10.1136/oemed-2013-101874

145.	Urman R, McConnell R, Islam T, et al. Associations of children's lung function with ambient air pollution: joint effects of regional and near-roadway pollutants. *Thorax*. 2014;doi:10.1136/thoraxjnl-2012-203159

146.	Neophytou AM, White MJ, Oh SS, et al. "Air Pollution and Lung Function in Minority Youth with Asthma in the GALA II (Genes-Environments and Admixture in Latino Americans) and SAGE II (Study of African Americans, Asthma, Genes, and Environments) Studies". *Am J Respir Crit Care Med*. 2016;doi:10.1164/rccm.201508-1706OC

147.	Schachter EN, Moshier E, Habre R, et al. Outdoor air pollution and health effects in urban children with moderate to severe asthma. *Air Quality, Atmosphere & Health*. 2016/04/01 2016;9(3):251-263. doi:10.1007/s11869-015-0335-6

148.	Gilliland F, Avol E, McConnell R, et al. The Effects of Policy-Driven Air Quality Improvements on Children's Respiratory Health. *Res Rep Health Eff Inst*. 2017;

149.	Magzamen S, Oron AP, Locke ER, Fan VS. Association of ambient pollution with inhaler use among patients with COPD: a panel study. *Occup Environ Med*. 2018;doi:10.1136/oemed-2017-104808

150.	Aaron CP, Hoffman EA, Kawut SM, et al. Ambient air pollution and pulmonary vascular volume on computed tomography: the MESA Air Pollution and Lung cohort studies. *Eur Respir J*. 2019;doi:10.1183/13993003.02116-2018

151.	Benka-Coker WO, Loftus C, Karr C, Magzamen S. Characterizing the joint effects of pesticide exposure and criteria ambient air pollutants on pediatric asthma morbidity in an agricultural community. *Environ Epidemiol*. Jun 2019;3(3):e046. doi:10.1097/ee9.0000000000000046

152.	Benka-Coker W, Hoskovec L, Severson R, Balmes J, Wilson A, Magzamen S. The joint effect of ambient air pollution and agricultural pesticide exposures on lung function among children with asthma. *Environ Res*. Nov 2020;190:109903. doi:10.1016/j.envres.2020.109903

153.	Martenies SE, Schaeffer JW, Erlandson G, et al. Associations Between Bioaerosol Exposures and Lung Function Changes Among Dairy Workers in Colorado. *J Occup Environ Med*. Jun 2020;62(6):424-430. doi:10.1097/JOM.0000000000001856

154.	Marcus M. Pollution at schools and children's aerobic capacity. *Health Econ*. 2021;doi:10.1002/hec.4425

155.	Hao H, Eckel SP, Hosseini A, et al. Daily Associations of Air Pollution and Pediatric Asthma Risk Using the Biomedical REAI-Time Health Evaluation (BREATHE) Kit. *Int J Environ Res Public Health*. 2022;doi:10.3390/ijerph19063578

156.	Chen KLY, Aglan M, Purcell A, et al. Physical Activity, Air Pollution Exposure, and Lung Function Interactions Among Adults with COPD. *Chronic Obstructive Pulmonary Diseases-Journal of the Copd Foundation*. 2023;doi:10.15326/jcopdf.2022.0385

157.	Neophytou AM, Lutzker L, Good KM, et al. Associations between prenatal and early-life air pollution exposure and lung function in young children: Exploring influential windows of exposure on lung development. *Environ Res*. 2023;doi:10.1016/j.envres.2023.115415

158.	Hazlehurst MF, Dearborn LC, Sherris AR, et al. Long-term ozone exposure and lung function in middle childhood. *Environmental Research*. 2024;doi:10.1016/j.envres.2023.117632

159.	Dearborn LC, Hazlehurst MF, Melough MM, et al. Prenatal ozone exposure and child lung function: Exploring effect modification by oxidative balance score. *Int J Hyg Environ Health*. Mar 2025;264:114491. doi:10.1016/j.ijheh.2024.114491

160.	Sack C, Wojdyla DM, MacMurdo MG, et al. Long-Term Air Pollution Exposure and Severity of Idiopathic Pulmonary Fibrosis: Data from the Idiopathic Pulmonary Fibrosis Prospective Outcomes (IPF-PRO) Registry. *Ann Am Thorac Soc*. Mar 2025;22(3):378-386. doi:10.1513/AnnalsATS.202404-382OC

161.	Xue S, Broerman MJ, Goobie GC, et al. Gaseous Air Pollutants and Lung Function in Fibrotic Interstitial Lung Disease (fILD): Evaluation of Different Spatial Analysis Approaches. *Environ Sci Technol*. Apr 1 2025;59(12):5936-5945. doi:10.1021/acs.est.4c11275

162.	Perry GB, Chai H, Dickey DW, et al. Effects of particulate air pollution on asthmatics. *Am J Public Health*. Jan 1983;73(1):50-6. doi:10.2105/ajph.73.1.50

163.	Greer JR, Abbey DE, Burchette RJ. Asthma Related to Occupational and Ambient Air Pollutants in Nonsmokers. *Journal of Occupational and Environmental Medicine*. 1993;35(9):909-915. doi:10.1097/00043764-199309000-00014

164.	Abbey DE, Lebowitz MD, Mills PK, Petersen FF, Beeson WL, Burchette RJ. Long-Term Ambient Concentrations of Particulates and Oxidants and Development of Chronic Disease in a Cohort of Nonsmoking California Residents. *Inhalation Toxicology*. 1995/01/01 1995;7(1):19-34. doi:10.3109/08958379509014268

165.	Avol EL, Navidi WC, Rappaport EB, Peters JM. Acute effects of ambient ozone on asthmatic, wheezy, and healthy children. *Res Rep Health Eff Inst*. May 1998;(82):iii, 1-18; discussion 19-30.

166.	Delfino RJ, Zeiger RS, Seltzer JM, Street DH. Symptoms in pediatric asthmatics and air pollution: differences in effects by symptom severity, anti-inflammatory medication use and particulate averaging time. *Environ Health Perspect*. Nov 1998;106(11):751-61. doi:10.1289/ehp.98106751

167.	Ostro B, Lipsett M, Mann J, Braxton-Owens H, White M. Air pollution and exacerbation of asthma in African-American children in Los Angeles. *Epidemiology*. Mar 2001;12(2):200-8. doi:10.1097/00001648-200103000-00012

168.	Millstein J, Gilliland F, Berhane K, et al. Effects of ambient air pollutants on asthma medication use and wheezing among fourth-grade school children from 12 Southern California communities enrolled in The Children's Health Study. *Arch Environ Health*. Oct 2004;59(10):505-14. doi:10.1080/00039890409605166

169.	Rabinovitch N, Zhang L, Murphy JR, Vedal S, Dutton SJ, Gelfand EW. Effects of wintertime ambient air pollutants on asthma exacerbations in urban minority children with moderate to severe disease. *J Allergy Clin Immunol*. Nov 2004;114(5):1131-7. doi:10.1016/j.jaci.2004.08.026

170.	Schildcrout JS, Sheppard L, Lumley T, Slaughter JC, Koenig JQ, Shapiro GG. Ambient air pollution and asthma exacerbations in children: an eight-city analysis. *Am J Epidemiol*. Sep 15 2006;164(6):505-17. doi:10.1093/aje/kwj225

171.	O'Connor GT, Neas L, Vaughn B, et al. Acute respiratory health effects of air pollution on children with asthma in US inner cities. *J Allergy Clin Immunol*. May 2008;121(5):1133-1139.e1. doi:10.1016/j.jaci.2008.02.020

172.	Mann JK, Balmes JR, Bruckner TA, et al. Short-term effects of air pollution on wheeze in asthmatic children in Fresno, California. *Environ Health Perspect*. Oct 2010;118(10):1497-502. doi:10.1289/ehp.0901292

173.	McConnell R, Islam T, Shankardass K, et al. Childhood incident asthma and traffic-related air pollution at home and school. *Environ Health Perspect*. Jul 2010;118(7):1021-6. doi:10.1289/ehp.0901232

174.	Nishimura KK, Galanter JM, Roth LA, et al. Early-life air pollution and asthma risk in minority children. The GALA II and SAGE II studies. *Am J Respir Crit Care Med*. Aug 1 2013;188(3):309-18. doi:10.1164/rccm.201302-0264OC

175.	Li T, Lin G. Examining the role of location-specific associations between ambient air pollutants and adult asthma in the United States. *Health Place*. Jan 2014;25:26-33. doi:10.1016/j.healthplace.2013.10.007

176.	Wendt JK, Symanski E, Stock TH, Chan W, Du XL. Association of short-term increases in ambient air pollution and timing of initial asthma diagnosis among Medicaid-enrolled children in a metropolitan area. *Environ Res*. May 2014;131:50-8. doi:10.1016/j.envres.2014.02.013

177.	Brewer M, Kimbro RT, Denney JT, Osiecki KM, Moffett B, Lopez K. Does neighborhood social and environmental context impact race/ethnic disparities in childhood asthma? *Health Place*. Mar 2017;44:86-93. doi:10.1016/j.healthplace.2017.01.006

178.	Cox LAT, Jr. Socioeconomic and air pollution correlates of adult asthma, heart attack, and stroke risks in the United States, 2010-2013. *Environ Res*. May 2017;155:92-107. doi:10.1016/j.envres.2017.01.003

179.	Li Z, Xu X, Thompson LA, et al. Longitudinal Effect of Ambient Air Pollution and Pollen Exposure on Asthma Control: The Patient-Reported Outcomes Measurement Information System (PROMIS) Pediatric Asthma Study. *Acad Pediatr*. Aug 2019;19(6):615-623. doi:10.1016/j.acap.2019.03.010

180.	Huang W, Schinasi LH, Kenyon CC, et al. Effects of ambient air pollution on childhood asthma exacerbation in the Philadelphia metropolitan Region, 2011-2014. *Environ Res*. Jun 2021;197:110955. doi:10.1016/j.envres.2021.110955

181.	Hurbain P, Liu Y, Strickland MJ, Li D. A cross-sectional analysis of associations between environmental indices and asthma in U.S. counties from 2003 to 2012. *J Expo Sci Environ Epidemiol*. Mar 2022;32(2):320-332. doi:10.1038/s41370-021-00326-4

182.	Su JG, Barrett MA, Combs V, et al. Identifying impacts of air pollution on subacute asthma symptoms using digital medication sensors. *Int J Epidemiol*. Feb 18 2022;51(1):213-224. doi:10.1093/ije/dyab187

183.	Mirabelli MC, Flanders WD, Vaidyanathan A, Beavers DP, Gower WA. Ambient Air Quality and Fatal Asthma Exacerbations among Children in North Carolina. *Epidemiology*. Nov 1 2023;34(6):888-891. doi:10.1097/ede.0000000000001648

184.	Robles TF, Bai S, Meng YY. Ozone Pollution, Perceived Support at Home, and Asthma Symptom Severity in the Adolescent Sample of the California Health Interview Survey. *Int J Behav Med*. Jun 2023;30(3):398-408. doi:10.1007/s12529-022-10103-8

185.	Hazlehurst MF, Carroll KN, Moore PE, et al. Associations of prenatal ambient air pollution exposures with asthma in middle childhood. *Int J Hyg Environ Health*. May 2024;258:114333. doi:10.1016/j.ijheh.2024.114333

186.	Kelchtermans J, Mentch F, Hakonarson H. Ambient air pollution sensitivity and severity of pediatric asthma. *J Expo Sci Environ Epidemiol*. Sep 2024;34(5):853-860. doi:10.1038/s41370-023-00573-7

187.	Amialchuk A, Sapci O. The Effect of Long-Term Exposure to O(3) and PM(2.5) on Allergies and Asthma in Adolescents and Young Adults. *Int J Environ Res Public Health*. Aug 12 2025;22(8)doi:10.3390/ijerph22081262

188.	Byrwa-Hill BM, Morphew T, O'Neill J, Gentile D. Air Quality Index as a Predictor of Respiratory Morbidity in At-Risk Populations. *Int J Environ Res Public Health*. Sep 27 2025;22(10)doi:10.3390/ijerph22101493

189.	Dearborn LC, Hazlehurst MF, Sherris AR, et al. Early-Life Ozone Exposure and Asthma and Wheeze in Children. *JAMA Netw Open*. Apr 1 2025;8(4):e254121. doi:10.1001/jamanetworkopen.2025.4121

190.	Lu W, Eisen EA, Lutzker L, et al. Mixture analysis identifies ozone as driving factor for child asthma symptoms. *J Allergy Clin Immunol Glob*. Aug 2025;4(3):100513. doi:10.1016/j.jacig.2025.100513

191.	Po J, Morrison J, Marian B, Chen Z, James Gauderman W, Garcia E. Gene-Air Pollution Interaction and Diversity of Genetic Sampling: The Southern California Children's Health Study. *Genet Epidemiol*. Jan 2025;49(1):e70000. doi:10.1002/gepi.70000

192.	Wang VA, Habre R, Ryan PH, et al. Disparities in the association of ambient air pollution with childhood asthma incidence in the ECHO consortium: A US-wide multi-cohort study. *Environ Epidemiol*. Aug 2025;9(4):e398. doi:10.1097/ee9.0000000000000398

193.	Wang JG, Li W, Liu B, et al. Long-Term Air Pollution Exposure, Plant-based Diet, and Asthma Exacerbations in the Nurses' Health Study II. *Ann Am Thorac Soc*. Jan 1 2026;23(1):47-55. doi:10.1513/AnnalsATS.202501-054OC

194.	Cody RP, Weisel CP, Birnbaum G, Lioy PJ. The effect of ozone associated with summertime photochemical smog on the frequency of asthma visits to hospital emergency departments. *Environ Res*. Aug 1992;58(2):184-94. doi:10.1016/s0013-9351(05)80214-2

195.	White MC, Etzel RA, Wilcox WD, Lloyd C. Exacerbations of childhood asthma and ozone pollution in Atlanta. *Environ Res*. Apr 1994;65(1):56-68. doi:10.1006/enrs.1994.1021

196.	Weisel CP, Cody RP, Lioy PJ. Relationship between summertime ambient ozone levels and emergency department visits for asthma in central New Jersey. *Environ Health Perspect*. Mar 1995;103 Suppl 2(Suppl 2):97-102. doi:10.1289/ehp.95103s297

197.	Carlin BP, Xia H. Assessing environmental justice using Bayesian hierarchical models: two case studies. *Journal of Exposure Analysis and Environmental Epidemiology*. 1999;9:66-78.

198.	Cassino C, Ito K, Bader I, Ciotoli C, Thurston G, Reibman J. Cigarette smoking and ozone-associated emergency department use for asthma by adults in New York City. *Am J Respir Crit Care Med*. Jun 1999;159(6):1773-9. doi:10.1164/ajrccm.159.6.9809042

199.	Sheppard L, Levy D, Norris G, Larson TV, Koenig JQ. Effects of ambient air pollution on nonelderly asthma hospital admissions in Seattle, Washington, 1987-1994. *Epidemiology*. Jan 1999;10(1):23-30.

200.	Tolbert PE, Mulholland JA, MacIntosh DL, et al. Air quality and pediatric emergency room visits for asthma in Atlanta, Georgia, USA. *Am J Epidemiol*. Apr 15 2000;151(8):798-810. doi:10.1093/oxfordjournals.aje.a010280

201.	Tolbert PE, Klein M, Metzger KB, et al. Interim results of the study of particulates and health in Atlanta (SOPHIA). *J Expo Anal Environ Epidemiol*. Sep-Oct 2000;10(5):446-60. doi:10.1038/sj.jea.7500106

202.	Friedman MS, Powell KE, Hutwagner L, Graham LM, Teague WG. Impact of changes in transportation and commuting behaviors during the 1996 Summer Olympic Games in Atlanta on air quality and childhood asthma. *Jama*. Feb 21 2001;285(7):897-905. doi:10.1001/jama.285.7.897

203.	Weisel CP, Cody RP, Georgopoulos PG, et al. Concepts in developing health-based indicators for ozone. *Int Arch Occup Environ Health*. Aug 2002;75(6):415-22. doi:10.1007/s00420-002-0318-4

204.	Jaffe DH, Singer ME, Rimm AA. Air pollution and emergency department visits for asthma among Ohio Medicaid recipients, 1991-1996. *Environ Res*. Jan 2003;91(1):21-8. doi:10.1016/s0013-9351(02)00004-x

205.	Peel JL, Tolbert PE, Klein M, et al. Ambient air pollution and respiratory emergency department visits. *Epidemiology*. Mar 2005;16(2):164-74. doi:10.1097/01.ede.0000152905.42113.db

206.	Wilson AM, Wake CP, Kelly T, Salloway JC. Air pollution, weather, and respiratory emergency room visits in two northern New England cities: an ecological time-series study. *Environ Res*. Mar 2005;97(3):312-21. doi:10.1016/j.envres.2004.07.010

207.	Tolbert PE, Klein M, Peel JL, Sarnat SE, Sarnat JA. Multipollutant modeling issues in a study of ambient air quality and emergency department visits in Atlanta. *J Expo Sci Environ Epidemiol*. Dec 2007;17 Suppl 2:S29-35. doi:10.1038/sj.jes.7500625

208.	Babin S, Burkom H, Holtry R, Tabernero N, Davies-Cole J. Air Quality Effects on Health-Indicator Data in Disease Outbreak Surveillance. *Johns Hopkins APL Technical Digest (Applied Physics Laboratory)*. 01/01 2008;27:393-402.

209.	Babin S, Burkom H, Holtry R, et al. Medicaid patient asthma-related acute care visits and their associations with ozone and particulates in Washington, DC, from 1994-2005. *Int J Environ Health Res*. Jun 2008;18(3):209-21. doi:10.1080/09603120701694091

210.	Lin S, Liu X, Le LH, Hwang SA. Chronic exposure to ambient ozone and asthma hospital admissions among children. *Environ Health Perspect*. Dec 2008;116(12):1725-30. doi:10.1289/ehp.11184

211.	Moore K, Neugebauer R, Lurmann F, et al. Ambient ozone concentrations cause increased hospitalizations for asthma in children: an 18-year study in Southern California. *Environ Health Perspect*. Aug 2008;116(8):1063-70. doi:10.1289/ehp.10497

212.	Paulu C, Smith AE. Tracking associations between ambient ozone and asthma-related emergency department visits using case-crossover analysis. *J Public Health Manag Pract*. Nov-Dec 2008;14(6):581-91. doi:10.1097/01.PHH.0000338371.53242.0e

213.	Neidell M, Kinney PL. Estimates of the association between ozone and asthma hospitalizations that account for behavioral responses to air quality information. *Environmental Science & Policy*. 2010/04/01/ 2010;13(2):97-103. doi:<https://doi.org/10.1016/j.envsci.2009.12.006>

214.	Peel JL, Klein M, Flanders WD, Mulholland JA, Tolbert PE. Impact of improved air quality during the 1996 Summer Olympic Games in Atlanta on multiple cardiovascular and respiratory outcomes. *Res Rep Health Eff Inst*. Apr 2010;(148):3-23; discussion 25-33.

215.	Silverman RA, Ito K. Age-related association of fine particles and ozone with severe acute asthma in New York City. *J Allergy Clin Immunol*. Feb 2010;125(2):367-373.e5. doi:10.1016/j.jaci.2009.10.061

216.	Sinclair AH, Edgerton ES, Wyzga R, Tolsma D. A two-time-period comparison of the effects of ambient air pollution on outpatient visits for acute respiratory illnesses. *J Air Waste Manag Assoc*. Feb 2010;60(2):163-75. doi:10.3155/1047-3289.60.2.163

217.	Strickland MJ, Darrow LA, Klein M, et al. Short-term associations between ambient air pollutants and pediatric asthma emergency department visits. *Am J Respir Crit Care Med*. Aug 1 2010;182(3):307-16. doi:10.1164/rccm.200908-1201OC

218.	Hanna AF, Yeatts KB, Xiu A, et al. Associations between ozone and morbidity using the Spatial Synoptic Classification system. *Environ Health*. May 24 2011;10:49. doi:10.1186/1476-069x-10-49

219.	Strickland MJ, Darrow LA, Mulholland JA, et al. Implications of different approaches for characterizing ambient air pollutant concentrations within the urban airshed for time-series studies and health benefits analyses. *Environmental Health*. 2011/05/11 2011;10(1):36. doi:10.1186/1476-069X-10-36

220.	Delamater PL, Finley AO, Banerjee S. An analysis of asthma hospitalizations, air pollution, and weather conditions in Los Angeles County, California. *Sci Total Environ*. May 15 2012;425:110-8. doi:10.1016/j.scitotenv.2012.02.015

221.	Glad JA, Brink LL, Talbott EO, et al. The relationship of ambient ozone and PM(2.5) levels and asthma emergency department visits: possible influence of gender and ethnicity. *Arch Environ Occup Health*. 2012;67(2):103-8. doi:10.1080/19338244.2011.598888

222.	Winquist A, Klein M, Tolbert P, Flanders WD, Hess J, Sarnat SE. Comparison of emergency department and hospital admissions data for air pollution time-series studies. *Environ Health*. Sep 21 2012;11:70. doi:10.1186/1476-069x-11-70

223.	Anderko L, Davies-Cole J, Strunk A. Identifying populations at risk: interdisciplinary environmental climate change tracking. *Public Health Nurs*. Nov-Dec 2014;31(6):484-91. doi:10.1111/phn.12164

224.	Delfino RJ, Wu J, Tjoa T, Gullesserian SK, Nickerson B, Gillen DL. Asthma Morbidity and Ambient Air Pollution: Effect Modification by Residential Traffic-Related Air Pollution. *Epidemiology*. 2014;25(1):48-57. doi:10.1097/ede.0000000000000016

225.	Gass K, Klein M, Chang HH, Flanders WD, Strickland MJ. Classification and regression trees for epidemiologic research: an air pollution example. *Environ Health*. Mar 13 2014;13(1):17. doi:10.1186/1476-069x-13-17

226.	Raun LH, Ensor KB, Persse D. Using community level strategies to reduce asthma attacks triggered by outdoor air pollution: a case crossover analysis. *Environ Health*. Jul 11 2014;13:58. doi:10.1186/1476-069x-13-58

227.	Sacks JD, Rappold AG, Davis JA, Jr., Richardson DB, Waller AE, Luben TJ. Influence of urbanicity and county characteristics on the association between ozone and asthma emergency department visits in North Carolina. *Environ Health Perspect*. May 2014;122(5):506-12. doi:10.1289/ehp.1306940

228.	Strickland MJ, Klein M, Flanders WD, et al. Modification of the effect of ambient air pollution on pediatric asthma emergency visits: susceptible subpopulations. *Epidemiology*. Nov 2014;25(6):843-50. doi:10.1097/ede.0000000000000170

229.	Winquist A, Kirrane E, Klein M, et al. Joint effects of ambient air pollutants on pediatric asthma emergency department visits in Atlanta, 1998-2004. *Epidemiology*. Sep 2014;25(5):666-73. doi:10.1097/ede.0000000000000146

230.	Gass K, Klein M, Sarnat SE, et al. Associations between ambient air pollutant mixtures and pediatric asthma emergency department visits in three cities: a classification and regression tree approach. *Environ Health*. Jun 27 2015;14:58. doi:10.1186/s12940-015-0044-5

231.	Gleason JA, Fagliano JA. Associations of daily pediatric asthma emergency department visits with air pollution in Newark, NJ: utilizing time-series and case-crossover study designs. *Journal of Asthma*. 2015/09/14 2015;52(8):815-822. doi:10.3109/02770903.2015.1033726

232.	Pearce JL, Waller LA, Mulholland JA, et al. Exploring associations between multipollutant day types and asthma morbidity: epidemiologic applications of self-organizing map ambient air quality classifications. *Environ Health*. Jun 23 2015;14:55. doi:10.1186/s12940-015-0041-8

233.	Rodopoulou S, Samoli E, Chalbot MG, Kavouras IG. Air pollution and cardiovascular and respiratory emergency visits in Central Arkansas: A time-series analysis. *Sci Total Environ*. Dec 1 2015;536:872-879. doi:10.1016/j.scitotenv.2015.06.056

234.	Sheffield PE, Zhou J, Shmool JL, Clougherty JE. Ambient ozone exposure and children's acute asthma in New York City: a case-crossover analysis. *Environ Health*. Mar 18 2015;14:25. doi:10.1186/s12940-015-0010-2

235.	Malig BJ, Pearson DL, Chang YB, et al. A Time-Stratified Case-Crossover Study of Ambient Ozone Exposure and Emergency Department Visits for Specific Respiratory Diagnoses in California (2005-2008). *Environ Health Perspect*. Jun 2016;124(6):745-53. doi:10.1289/ehp.1409495

236.	Shmool JL, Kinnee E, Sheffield PE, Clougherty JE. Spatio-temporal ozone variation in a case-crossover analysis of childhood asthma hospital visits in New York City. *Environ Res*. May 2016;147:108-14. doi:10.1016/j.envres.2016.01.020

237.	Xiao Q, Liu Y, Mulholland JA, et al. Pediatric emergency department visits and ambient Air pollution in the U.S. State of Georgia: a case-crossover study. *Environ Health*. Nov 25 2016;15(1):115. doi:10.1186/s12940-016-0196-y

238.	Goodman JE, Loftus CT, Liu X, Zu K. Impact of respiratory infections, outdoor pollen, and socioeconomic status on associations between air pollutants and pediatric asthma hospital admissions. *PLoS One*. 2017;12(7):e0180522. doi:10.1371/journal.pone.0180522

239.	CR OL, Chang HH, Kramer MR, et al. Ozone and childhood respiratory disease in three US cities: evaluation of effect measure modification by neighborhood socioeconomic status using a Bayesian hierarchical approach. *Environ Health*. Apr 5 2017;16(1):36. doi:10.1186/s12940-017-0244-2

240.	Zu K, Liu X, Shi L, et al. Concentration-response of short-term ozone exposure and hospital admissions for asthma in Texas. *Environ Int*. Jul 2017;104:139-145. doi:10.1016/j.envint.2017.04.006

241.	Castner J, Guo L, Yin Y. Ambient air pollution and emergency department visits for asthma in Erie County, New York 2007-2012. *Int Arch Occup Environ Health*. Feb 2018;91(2):205-214. doi:10.1007/s00420-017-1270-7

242.	Krall JR, Chang HH, Waller LA, et al. A multicity study of air pollution and cardiorespiratory emergency department visits: Comparing approaches for combining estimates across cities. *Environ Int*. Nov 2018;120:312-320. doi:10.1016/j.envint.2018.07.033

243.	Barry V, Klein M, Winquist A, et al. Characterization of the concentration-response curve for ambient ozone and acute respiratory morbidity in 5 US cities. *J Expo Sci Environ Epidemiol*. Mar 2019;29(2):267-277. doi:10.1038/s41370-018-0048-7

244.	Gharibi H, Entwistle MR, Ha S, et al. Ozone pollution and asthma emergency department visits in the Central Valley, California, USA, during June to September of 2015: a time-stratified case-crossover analysis. *J Asthma*. Oct 2019;56(10):1037-1048. doi:10.1080/02770903.2018.1523930

245.	Lovinsky-Desir S, Acosta LM, Rundle AG, et al. Air pollution, urgent asthma medical visits and the modifying effect of neighborhood asthma prevalence. *Pediatr Res*. Jan 2019;85(1):36-42. doi:10.1038/s41390-018-0189-3

246.	Mendy A, Wilkerson J, Salo PM, et al. Synergistic Association of House Endotoxin Exposure and Ambient Air Pollution with Asthma Outcomes. *Am J Respir Crit Care Med*. Sep 15 2019;200(6):712-720. doi:10.1164/rccm.201809-1733OC

247.	Reid CE, Considine EM, Watson GL, Telesca D, Pfister GG, Jerrett M. Associations between respiratory health and ozone and fine particulate matter during a wildfire event. *Environ Int*. 2019;doi:10.1016/j.envint.2019.04.033

248.	Witonsky J, Abraham R, Toh J, et al. The association of environmental, meteorological, and pollen count variables with asthma-related emergency department visits and hospitalizations in the Bronx. *J Asthma*. Sep 2019;56(9):927-937. doi:10.1080/02770903.2018.1514627

249.	Baek J, Kash BA, Xu X, Benden M, Roberts J, Carrillo G. Association between Ambient Air Pollution and Hospital Length of Stay among Children with Asthma in South Texas. *Int J Environ Res Public Health*. May 27 2020;17(11)doi:10.3390/ijerph17113812

250.	Baek J, Kash BA, Xu X, Benden M, Roberts J, Carrillo G. Effect of Ambient Air Pollution on Hospital Readmissions among the Pediatric Asthma Patient Population in South Texas: A Case-Crossover Study. *Int J Environ Res Public Health*. Jul 6 2020;17(13)doi:10.3390/ijerph17134846

251.	Byrwa-Hill BM, Venkat A, Presto AA, Rager JR, Gentile D, Talbott E. Lagged Association of Ambient Outdoor Air Pollutants with Asthma-Related Emergency Department Visits within the Pittsburgh Region. *Int J Environ Res Public Health*. Nov 20 2020;17(22)doi:10.3390/ijerph17228619

252.	Rosenquist NA, Metcalf WJ, Ryu SY, et al. Acute associations between PM(2.5) and ozone concentrations and asthma exacerbations among patients with and without allergic comorbidities. *J Expo Sci Environ Epidemiol*. Sep 2020;30(5):795-804. doi:10.1038/s41370-020-0213-7

253.	Baek J, Kash BA, Xu X, Benden M, Roberts J, Carrillo G. Pediatric asthma hospitalization: individual and environmental characteristics of high utilizers in South Texas. *J Asthma*. Jan 2022;59(1):94-104. doi:10.1080/02770903.2020.1827424

254.	Fecho K, Ahalt SC, Appold S, et al. Development and Application of an Open Tool for Sharing and Analyzing Integrated Clinical and Environmental Exposures Data: Asthma Use Case. *JMIR Form Res*. Apr 1 2022;6(4):e32357. doi:10.2196/32357

255.	Park J-H, Lee E, Fechter-Leggett E, et al. Associations of Emergency Department Visits for Asthma with Precipitation and Temperature on Thunderstorm Days: A Time-Series Analysis of Data from Louisiana, USA, 2010–2012. *Environmental Health Perspectives*. 08/01 2022;130doi:10.1289/EHP10440

256.	Rosser F, Han YY, Rothenberger SD, Forno E, Mair C, Celedón JC. Air Quality Index and Emergency Department Visits and Hospitalizations for Childhood Asthma. *Ann Am Thorac Soc*. Jul 2022;19(7):1139-1148. doi:10.1513/AnnalsATS.202105-539OC

257.	Bi J, D'Souza RR, Moss S, et al. Acute Effects of Ambient Air Pollution on Asthma Emergency Department Visits in Ten U.S. States. *Environ Health Perspect*. Apr 2023;131(4):47003. doi:10.1289/ehp11661

258.	Sharma R, Humphrey JL, Frueh L, Kinnee EJ, Sheffield PE, Clougherty JE. Neighborhood violence and socioeconomic deprivation influence associations between acute air pollution and temperature on childhood asthma in New York city. *Environ Res*. Aug 15 2023;231(Pt 3):116235. doi:10.1016/j.envres.2023.116235

259.	Horne BD, Johnson MM, Blagev DP, et al. Association of Short-Term Increases in Ambient Fine Particulate Matter With Hospitalization for Asthma or COPD During Wildfire Season and Other Time Periods. *CHEST Pulm*. Jun 2024;2(2)doi:10.1016/j.chpulm.2024.100053

260.	Mills PK, Abbey D, Beeson WL, Petersen F. Ambient air pollution and cancer in California Seventh-day Adventists. *Arch Environ Health*. 1991;doi:10.1080/00039896.1991.9934387

261.	Samet JM, Dominici F, Curriero FC, Coursac I, Zeger SL. Fine particulate air pollution and mortality in 20 U.S. cities, 1987-1994. *N Engl J Med*. 2000;doi:10.1056/nejm200012143432401

262.	Woodruff TJ, Darrow LA, Parker JD. "Air pollution and postneonatal infant mortality in the United States, 1999-2002". *Environ Health Perspect*. 2008;doi:10.1289/ehp.10370

263.	Zanobetti A, Schwartz J. Mortality displacement in the association of ozone with mortality - An analysis of 48 cities in the United States. *American Journal of Respiratory and Critical Care Medicine*. 2008;doi:10.1164/rccm.200706-823OC

264.	Zanobetti A, Schwartz J. Ozone and Survival in Four Cohorts with Potentially Predisposing Diseases. *American Journal of Respiratory and Critical Care Medicine*. 2011;doi:10.1164/rccm.201102-0227OC

265.	Peng RD, Samoli E, Pham L, et al. Acute effects of ambient ozone on mortality in Europe and North America: results from the APHENA study. *Air Qual Atmos Health*. 2013;doi:10.1007/s11869-012-0180-9

266.	Xu X, Ha S, Kan H, Hu H, Curbow BA, Lissaker CT. Health effects of air pollution on length of respiratory cancer survival. *BMC Public Health*. 2013;doi:10.1186/1471-2458-13-800

267.	Kravchenko J, Akushevich I, Abernethy AP, Holman S, W. G. Ross J, Lyerly HK. Long-term dynamics of death rates of emphysema, asthma, and pneumonia and improving air quality. *Int J Chron Obstruct Pulmon Dis*. 2014;doi:10.2147/copd.S59995

268.	Hao Y, Balluz L, Strosnider H, Wen XJ, Li C, Qualters JR. "Ozone, Fine Particulate Matter, and Chronic Lower Respiratory Disease Mortality in the United States". *Am J Respir Crit Care Med*. 2015;doi:10.1164/rccm.201410-1852OC

269.	Turner MC, Jerrett M, Pope CA, 3rd, et al. Long-Term Ozone Exposure and Mortality in a Large Prospective Study. *Am J Respir Crit Care Med*. May 15 2016;193(10):1134-42. doi:10.1164/rccm.201508-1633OC

270.	Rush B, McDermid RC, Celi LA, Walley KR, Russell JA, Boyd JH. Association between chronic exposure to air pollution and mortality in the acute respiratory distress syndrome. *Environ Pollut*. 2017;doi:10.1016/j.envpol.2017.02.014

271.	Yang L-S, Kleeman MJ, Cushing LJ, et al. Risks of source and species-specific air pollution for COVID-19 incidence and mortality in Los Angeles. *Environmental Research: Health*. Dec 1 2024;2(4)041001. doi:10.1088/2752-5309/ad67fb

272.	Azan A, Kannoth S, Zhang C, et al. Neighborhood environmental vulnerability factors strongly drove COVID-19 fatality and excess all-cause mortality in New York City, while long-term air pollutant associations were weak and varied. *The Science of the total environment*. 2025-Aug-10 2025;989:179874. doi:10.1016/j.scitotenv.2025.179874

273.	Kleeman M, Nau C, Su J, et al. Ambient Air Pollution and COVID-19 in California. *Res Rep Health Eff Inst*. Feb 2026;2026(238):1-97.

274.	Luben TJ, Krajewski AK, Wilkie AA, Foley K, Rappazzo KM. Matched case-control study of air pollution and infant mortality in North Carolina. *Environ Res*. May 15 2026;297:124117. doi:10.1016/j.envres.2026.124117

275.	Abbey DE, Mills PK, Petersen FF, Beeson WL. Long-term ambient concentrations of total suspended particulates and oxidants as related to incidence of chronic disease in California Seventh-Day Adventists. *Environ Health Perspect*. 1991;doi:10.1289/ehp.94-1567944

276.	Abbey DE, Petersen F, Mills PK, Beeson WL. "Long-term ambient concentrations of total suspended particulates, ozone, and sulfur dioxide and respiratory symptoms in a nonsmoking population". *Arch Environ Health*. 1993;doi:10.1080/00039896.1993.9938391

277.	Schwartz J. Air pollution and hospital admissions for the elderly in Birmingham, Alabama. *Am J Epidemiol*. Mar 15 1994;139(6):589-98. doi:10.1093/oxfordjournals.aje.a117048

278.	Schwartz J, Dockery DW, Neas LM, et al. Acute effects of summer air pollution on respiratory symptom reporting in children. *Am J Respir Crit Care Med*. 1994;doi:10.1164/ajrccm.150.5.7952546

279.	Jones GN, Sletten C, Mandry C, Brantley PJ. Ozone level effect on respiratory illness: an investigation of emergency department visits. *South Med J*. Oct 1995;88(10):1049-56. doi:10.1097/00007611-199510000-00010

280.	Schwartz J. Air pollution and hospital admissions for respiratory disease. *Epidemiology*. Jan 1996;7(1):20-8. doi:10.1097/00001648-199601000-00005

281.	Moolgavkar SH, Luebeck EG, Anderson EL. Air pollution and hospital admissions for respiratory causes in Minneapolis-St. Paul and Birmingham. *Epidemiology*. Jul 1997;8(4):364-70. doi:10.1097/00001648-199707000-00003

282.	Beeson WL, Abbey DE, Knutsen SF. Long-term concentrations of ambient air pollutants and incident lung cancer in California adults: Results from the AHSMOG study. *Environmental Health Perspectives*. 1998;

283.	McConnell R, Berhane K, Gilliland F, et al. Air pollution and bronchitic symptoms in Southern California children with asthma. *Environ Health Perspect*. 1999;doi:10.1289/ehp.99107757

284.	Peters JM, Avol E, Navidi W, et al. A study of twelve southern California communities with differing levels and types of air pollution - I. Prevalence of respiratory morbidity. *American Journal of Respiratory and Critical Care Medicine*. 1999;

285.	Linn WS, Szlachcic Y, Gong H, Jr., Kinney PL, Berhane KT. Air pollution and daily hospital admissions in metropolitan Los Angeles. *Environ Health Perspect*. May 2000;108(5):427-34. doi:10.1289/ehp.00108427

286.	Moolgavkar SH. Air pollution and hospital admissions for chronic obstructive pulmonary disease in three metropolitan areas in the United States. *Inhal Toxicol*. 2000;12 Suppl 4:75-90. doi:10.1080/089583700750019512

287.	Moolgavkar SH, Hazelton W, Luebeck G, Levy D, Sheppard L. Air Pollution, Pollens, and Admissions for Chronic Respiratory Disease in King County, Washington. *Inhalation Toxicology*. 2000/01/01 2000;12(sup1):157-171. doi:10.1080/0895-8378.1987.11463190

288.	Gwynn RC, Thurston GD. The burden of air pollution: impacts among racial minorities. *Environ Health Perspect*. Aug 2001;109 Suppl 4(Suppl 4):501-6. doi:10.1289/ehp.01109s4501

289.	Ross MA, Persky VW, Scheff PA, et al. Effect of ozone and aeroallergens on the respiratory health of asthmatics. *Arch Environ Health*. Nov-Dec 2002;57(6):568-78. doi:10.1080/00039890209602090

290.	McConnell R, Berhane K, Gilliland F, et al. Prospective study of air pollution and bronchitic symptoms in children with asthma. *Am J Respir Crit Care Med*. 2003;doi:10.1164/rccm.200304-466OC

291.	Medina-Ramón M, Zanobetti A, Schwartz J. The effect of ozone and PM10 on hospital admissions for pneumonia and chronic obstructive pulmonary disease: a national multicity study. *Am J Epidemiol*. Mar 15 2006;163(6):579-88. doi:10.1093/aje/kwj078

292.	Triche EW, Gent JF, Holford TR, et al. Low-level ozone exposure and respiratory symptoms in infants. *Environ Health Perspect*. 2006;doi:10.1289/ehp.8559

293.	Karr C, Lumley T, Schreuder A, et al. Effects of subchronic and chronic exposure to ambient air pollutants on infant bronchiolitis. *Am J Epidemiol*. 2007;doi:10.1093/aje/kwk032

294.	Lin S, Bell EM, Liu W, Walker RJ, Kim NK, Hwang SA. Ambient ozone concentration and hospital admissions due to childhood respiratory diseases in New York State, 1991-2001. *Environ Res*. Sep 2008;108(1):42-7. doi:10.1016/j.envres.2008.06.007

295.	Parker JD, Akinbami LJ, Woodruff TJ. Air pollution and childhood respiratory allergies in the United States. *Environ Health Perspect*. 2009;doi:10.1289/ehp.11497

296.	Choi M, Curriero FC, Johantgen M, Mills ME, Sattler B, Lipscomb J. Association between ozone and emergency department visits: an ecological study. *Int J Environ Health Res*. Jun 2011;21(3):201-21. doi:10.1080/09603123.2010.533366

297.	Darrow LA, Klein M, Sarnat JA, et al. The use of alternative pollutant metrics in time-series studies of ambient air pollution and respiratory emergency department visits. *J Expo Sci Environ Epidemiol*. Jan-Feb 2011;21(1):10-9. doi:10.1038/jes.2009.49

298.	Peel JL, Klein M, Flanders WD, Mulholland JA, Freed G, Tolbert PE. Ambient air pollution and apnea and bradycardia in high-risk infants on home monitors. *Environ Health Perspect*. 2011;doi:10.1289/ehp.1002739

299.	Berhane K, Chang CC, McConnell R, et al. Association of Changes in Air Quality With Bronchitic Symptoms in Children in California, 1993-2012. *Jama*. 2016;doi:10.1001/jama.2016.3444

300.	Ware LB, Zhao Z, Koyama T, et al. Long-Term Ozone Exposure Increases the Risk of Developing the Acute Respiratory Distress Syndrome. *Am J Respir Crit Care Med*. 2016;doi:10.1164/rccm.201507-1418OC

301.	Sack C, Vedal S, Sheppard L, et al. Air pollution and subclinical interstitial lung disease: the Multi-Ethnic Study of Atherosclerosis (MESA) air-lung study. *Eur Respir J*. 2017;doi:10.1183/13993003.00559-2017

302.	Pearce JL, Neelon B, Bozigar M, Hunt KJ, Commodore A, Vena J. "Associations between multipollutant day types and select cardiorespiratory outcomes in Columbia, South Carolina, 2002 to 2013". *Environ Epidemiol*. 2018;doi:10.1097/ee9.0000000000000030

303.	Pirozzi CS, Jones BE, VanDerslice JA, Zhang Y, Paine R, 3rd, Dean NC. Short-Term Air Pollution and Incident Pneumonia. A Case-Crossover Study. *Ann Am Thorac Soc*. Apr 2018;15(4):449-459. doi:10.1513/AnnalsATS.201706-495OC

304.	Seeni I, Ha S, Nobles C, Liu D, Sherman S, Mendola P. Air pollution exposure during pregnancy: maternal asthma and neonatal respiratory outcomes. *Ann Epidemiol*. 2018;doi:10.1016/j.annepidem.2018.06.003

305.	Reilly JP, Zhao Z, Shashaty MGS, et al. Exposure to ambient air pollutants and acute respiratory distress syndrome risk in sepsis. *Intensive Care Med*. 2023;doi:10.1007/s00134-023-07148-y

306.	Glick AF, Tomopoulos S, Fierman AH, Elixhauser A, Trasande L. Association Between Outdoor Air Pollution Levels and Inpatient Outcomes in Pediatric Pneumonia Hospitalizations, 2007 to 2008. *Acad Pediatr*. May-Jun 2019;19(4):414-420. doi:10.1016/j.acap.2018.12.001

307.	Reilly JP, Zhao Z, Shashaty MGS, et al. Low to Moderate Air Pollutant Exposure and Acute Respiratory Distress Syndrome after Severe Trauma. *Am J Respir Crit Care Med*. 2019;doi:10.1164/rccm.201803-0435OC

308.	Danesh Yazdi M, Wang Y, Di Q, Zanobetti A, Schwartz J. Long-term exposure to PM(2.5) and ozone and hospital admissions of Medicare participants in the Southeast USA. *Environ Int*. Sep 2019;130:104879. doi:10.1016/j.envint.2019.05.073

309.	Cromar KR, Ghazipura M, Gladson LA, Perlmutt L. Evaluating the U.S. Air Quality Index as a risk communication tool: Comparing associations of index values with respiratory morbidity among adults in California. *PLoS One*. 2020;15(11):e0242031. doi:10.1371/journal.pone.0242031

310.	Fuller CH, Jones JW, Roblin DW. Evaluating changes in ambient ozone and respiratory-related healthcare utilization in the Washington, DC metropolitan area. *Environ Res*. Jul 2020;186:109603. doi:10.1016/j.envres.2020.109603

311.	Steurer MA, Costello J, Baer RJ, et al. Cohort study of respiratory hospital admissions, air quality and sociodemographic factors in preterm infants born in California. *Paediatr Perinat Epidemiol*. Mar 2020;34(2):130-138. doi:10.1111/ppe.12652

312.	Yazdi MD, Wang Y, Di Q, et al. Long-Term Association of Air Pollution and Hospital Admissions Among Medicare Participants Using a Doubly Robust Additive Model. *Circulation*. 2021;doi:10.1161/circulationaha.120.050252

313.	Belz DC, Woo H, Putcha N, et al. Ambient ozone effects on respiratory outcomes among smokers modified by neighborhood poverty: An analysis of SPIROMICS AIR. *Sci Total Environ*. 2022;doi:10.1016/j.scitotenv.2022.154694

314.	Danesh Yazdi M, Wei Y, Di Q, et al. The effect of long-term exposure to air pollution and seasonal temperature on hospital admissions with cardiovascular and respiratory disease in the United States: A difference-in-differences analysis. *Sci Total Environ*. Oct 15 2022;843:156855. doi:10.1016/j.scitotenv.2022.156855

315.	Aglan A, Synn AJ, Nurhussien L, et al. Personal and community-level exposure to air pollution and daily changes in respiratory symptoms and oxygen saturation among adults with COPD. *Hyg Environ Health Adv*. 2023;doi:10.1016/j.heha.2023.100052

316.	Elam T, Raiculescu S, Biswal S, Orestes M, Ramanathan M, Zhang ZY. Air Pollution Exposure and the Development of Chronic Rhinosinusitis in the Active Duty Population. *Military Medicine*. 2023;doi:10.1093/milmed/usab535

317.	Garcia E, Birnhak ZH, West S, et al. Childhood Air Pollution Exposure Associated with Self-reported Bronchitic Symptoms in Adulthood. *Am J Respir Crit Care Med*. 2024;doi:10.1164/rccm.202308-1484OC

318.	Aguilera R, Letellier N, Basu R, et al. Effects of Multiple Wildfire Smoke Pollutants (PM(2.5), PM(10), and Ozone) on Respiratory and Cardiovascular Hospitalizations in California (2006-2019). *Geohealth*. Dec 2025;9(12):e2025GH001510. doi:10.1029/2025gh001510

319.	Ferschl MB, Chen S, Collis S, Jacobson A, Kolodzie K. Association between preoperative tropospheric ozone exposure and adverse perioperative respiratory events in paediatric patients: a single-centre retrospective cohort study. *Br J Anaesth*. Apr 2026;136(4):1393-1400. doi:10.1016/j.bja.2025.12.008

320.	Kannoth S, Zhang C, Shafiq M, et al. The relationship between chronic air pollution exposure, neighborhood environmental vulnerability, and adverse COVID-19 morbidities among hospitalized New York City residents. *Environ Int*. Aug 2025;202:109660. doi:10.1016/j.envint.2025.109660

321.	Park SL, Lichtensztajn D, Yang J, et al. Ambient Air Pollution and Chronic Obstructive Pulmonary Disease: The Multiethnic Cohort Study. *Annals of the American Thoracic Society*. May 2025;22(5):698-706. doi:10.1513/AnnalsATS.202404-387OC

322.	Stingone JA, Lovinsky-Desir S, Kannoth S, et al. REACH-OUT: Race, Ethnicity, and Air Pollution in COVID-19 Hospitalization OUTcomes. *Res Rep Health Eff Inst*. Oct 2025;2025(230):1-55.

323.	Chen F, Niu Z, Mohazzab-Hosseinian S, et al. Susceptible windows of long-term childhood exposure to air pollution on adult self-reported bronchitic symptoms. *International Journal of Epidemiology*. Feb 2026;55(1)dyaf205. doi:10.1093/ije/dyaf205

324.	Ou JY, Ramsay JM, Kaddas HK, et al. Air Pollution and Cardiovascular Risks in Testicular Cancer Survivors: An Exploratory Case-Crossover Study. *J Adolesc Young Adult Oncol*. Feb 2026;15(1):58-68. doi:10.1089/jayao.2024.0144

325.	Ramsay JM, Kaddas HK, VanDerslice JA, et al. Associations between Ambient Air Pollution and Cardiovascular and Respiratory Healthcare Visits in Adolescent and Young Adult Cancer Survivors. *Cancer Epidemiol Biomarkers Prev*. Apr 1 2026;35(4):628-636. doi:10.1158/1055-9965.Epi-25-1075

326.	Zhang D, Ebelt ST, Scovronick NC, Chang HH. Modeling Time-varying Dispersion to Improve Estimation of the Short-term Health Effect of Environmental Exposure in a Time-series Design. *Epidemiology*. Jul 1 2025;36(4):450-457. doi:10.1097/ede.0000000000001856

327.	Zhao X, Melix B, Powell E, Harville EW, Uejio CK. Combined effect of heat and outdoor O3 pollution on emergency department visits in Florida. *Environmental Research*. Feb 15 2026;291123561. doi:10.1016/j.envres.2025.123561
